# Mobile Critical Care Recovery Program for Survivors of Acute Respiratory Failure: A Randomized Clinical Trial

**DOI:** 10.1001/jamanetworkopen.2023.53158

**Published:** 2024-01-30

**Authors:** Babar A. Khan, Anthony J. Perkins, Sikandar Hayat Khan, Frederick W. Unverzagt, Sue Lasiter, Sujuan Gao, Sophia Wang, Ben L. Zarzaur, Omar Rahman, Ahmed Eltarras, Hadi Qureshi, Malaz A. Boustani

**Affiliations:** 1 Department of Medicine, Indiana University School of Medicine, Indianapolis; 2 Indiana University Center for Aging Research, Indianapolis; 3 Regenstrief Institute Inc, Indianapolis, Indiana; 4 Indiana University Center for Health Innovation and Implementation Science, Indiana Clinical and Translational Sciences Institute, Indianapolis; 5 Department of Biostatistics, Indiana University School of Medicine, Indianapolis; 6 Department of Psychiatry, Indiana University School of Medicine, Indianapolis; 7 School of Nursing and Health Sciences, University of Missouri, Kansas City; 8 Department of Surgery, University of Wisconsin School of Medicine and Public Health, Madison

## Abstract

**Importance:**

Over 50% of Acute Respiratory Failure (ARF) survivors experience cognitive, physical, and psychological impairments that negatively impact their quality of life (QOL).

**Objective:**

To evaluate the efficacy of a post–intensive care unit (ICU) program, the Mobile Critical Care Recovery Program (m-CCRP) consisting of a nurse care coordinator supported by an interdisciplinary team, in improving the QOL of ARF survivors.

**Design, Setting, and Participants:**

This randomized clinical trial with concealed outcome assessments among ARF survivors was conducted from March 1, 2017, to April 30, 2022, with a 12-month follow-up. Patients were admitted to the ICU services of 4 Indiana hospitals (1 community, 1 county, 2 academic), affiliated with the Indiana University School of Medicine.

**Intervention:**

A 12-month nurse-led collaborative care intervention (m-CCRP) supported by an interdisciplinary group of clinicians (2 intensivists, 1 geriatrician, 1 ICU nurse, and 1 neuropsychologist) was compared with a telephone-based control. The intervention comprised longitudinal symptom monitoring coupled with nurse-delivered care protocols targeting cognition, physical function, personal care, mobility, sleep disturbances, pain, depression, anxiety, agitation or aggression, delusions or hallucinations, stress and physical health, legal and financial needs, and medication adherence.

**Main Outcomes and Measures:**

The primary outcome was QOL as measured by the 36-item Medical Outcomes Study Short Form Health Survey (SF-36) physical component summary (PCS) and mental component summary (MCS), with scores on each component ranging from 0-100, and higher scores indicating better health status.

**Results:**

In an intention-to-treat analysis among 466 ARF survivors (mean [SD] age, 56.1 [14.4] years; 250 [53.6%] female; 233 assigned to each group), the m-CCRP intervention for 12 months did not significantly improve the QOL compared with the control group (estimated difference in change from baseline between m-CCRP and control group: 1.61 [95% CI, −1.06 to 4.29] for SF-36 PCS; −2.50 [95% CI, −5.29 to 0.30] for SF-36 MCS. Compared with the control group, the rates of hospitalization were higher in the m-CCRP group (117 [50.2%] vs 95 [40.8%]; *P* = .04), whereas the 12-month mortality rates were not statistically significantly lower (24 [10.3%] vs 38 [16.3%]; *P* = .05).

**Conclusions and Relevance:**

Findings from this randomized clinical trial indicated that a nurse-led 12-month comprehensive interdisciplinary care intervention did not significantly improve the QOL of ARF survivors after ICU hospitalization. These results suggest that further research is needed to identify specific patient groups who could benefit from tailored post-ICU interventions.

**Trial Registration:**

ClinicalTrials.gov Identifier: NCT03053245

## Key Points

**Question:**

Does an intensive care unit (ICU) recovery program comprising a nurse care coordinator supported by an interdisciplinary team improve quality of life among acute respiratory failure (ARF) survivors?

**Findings:**

In this randomized clinical trial of 466 ARF survivors, a 12-month collaborative care ICU recovery intervention delivered through a nurse care coordinator and supported by an interdisciplinary team of clinicians (intensivists, geriatrician, nursing, and neuropsychologist) did not significantly improve the quality of life of ARF survivors.

**Meaning:**

These results suggest that further research is needed to identify specific patient groups who could derive benefit from more intensive in-home interventions to allow for improved recovery in survivors of critical illness.

## Teaser

This randomized clinical trial assesses whether a program comprising a nurse care coordinator supported by an interdisciplinary team improves the quality of life after discharge from the intensive care unit among survivors of acute respiratory failure.

## Introduction

Acute respiratory failure (ARF) accounts for 2 million admissions to intensive care units (ICU) annually in the US.^1^ Over 50% of ARF survivors experience long-term functional disability,^2,3^ cognitive impairment,^4,5^ depression,^5,6,7^ and anxiety,^8^ complications that are negatively associated with quality of life (QOL).^2,9^ This constellation of ICU sequelae has been designated as post–intensive care syndrome.^10^ There are community resources and rehabilitation services available to ARF survivors, but the fragmented nature of the health care system in the US is unable to integrate and coordinate care to provide meaningful recovery.^11,12,13^

The Indiana University Center for Aging Research has previously developed interdisciplinary interventions using care coordinators from nursing and social work disciplines that have improved care of patients living with dementia, depression, and frailty.^14,15,16^ Clinics focused on ICU recovery have been developed in the US^17^; however, traditional outpatient clinics offer limited access, leading to delayed evaluation and travel burden on ARF survivors.^18^ We developed a post-ICU intervention, the Mobile Critical Care Recovery Program (m-CCRP), consisting of a nurse care coordinator bringing the intervention to ARF survivors’ place of residence, supported by an interdisciplinary team. The scope of the m-CCRP intervention included ongoing longitudinal monitoring of post-ICU symptoms coupled with coordination of health care appointments and use of nonpharmacological protocols to optimize the unique health care needs of ICU survivors.

We tested the efficacy of the m-CCRP on health outcomes of ARF survivors after hospital discharge through a randomized clinical trial. We hypothesized that m-CCRP intervention would improve QOL, depression and anxiety symptoms, and cognition for ARF survivors and decrease acute health care utilization rates during 12 months of follow-up.

## Methods

The Indiana University institutional review board approved this randomized clinical trial, and patients provided written informed consent. Informal caregivers were provided with the option to enroll. A detailed description of the trial protocol has been published previously.^19^ and is provided in Supplement 1. We followed the Consolidated Standards of Reporting Trials (CONSORT) reporting guideline for randomized clinical trials.^20^

### Study Setting and Eligibility

We enrolled patients from March 1, 2017, to April 30, 2022, from 4 Indiana hospitals’ ICUs (1 community, 1 county, and 2 academic). Participant inclusion criteria were (1) at least 18 years of age; (2) ICU admission; (3) ARF requiring 24 hours or longer of mechanical ventilation, noninvasive positive pressure ventilation, or heated humidified high flow nasal canula; (4) English speaking; (5) able to give consent or have a legally authorized representative; and (6) telephone access. Participant exclusion criteria were (1) receipt of a cancer diagnosis with less than 1 year life expectancy, (2) acute neurological injury (ischemic or hemorrhagic stroke, traumatic brain injury, or neurosurgery), (3) neurodegenerative illness, (4) hearing loss, (5) legal blindness, (6) pregnant or nursing, (7) alcohol or substance use disorder, or (8) incarcerated.

### Randomization

Research staff obtained patient informed consent and baseline measures within 48 hours of hospital discharge and up to 6 weeks after discharge. Participants were randomly assigned in a 1:1 manner via a computer-generated randomization scheme to either the m-CCRP or control group stratified by hospital and discharge location (home or facility).

### m-CCRP Intervention

The m-CCRP intervention was led by a nurse care coordinator who conducted patient visits at home and health care facilities, collaborated weekly with the m-CCRP interdisciplinary team (critical care physicians, geriatrician, ICU nurse, and neuropsychologist), worked with patients’ physicians, coordinated health care appointments, implemented care plans, and monitored recovery (eMethods in Supplement 2). Within 1 week of randomization, the care coordinator assessed patients at their place of residence using the Healthy Aging Brain Care Monitor (HABC-M; scores ranged from 0 to 81, with higher scores indicating higher cognitive, functional, and behavioral symptom burden),^21,22,23^ Mini Mental State Examination (MMSE; scores ranged from 0 to 30 with higher scores indicating improved cognition),^24^ Timed Up and Go test (scores ≤10 seconds were considered normal, and scores ≥14 seconds suggested high risk of falls),^25^ activities of daily living (Katz scale; scores range from 0 to 6, with higher scores indicating higher independence),^26^ instrumental activities of daily living (Lawton Scale; scores ranged from 0 to 8, with higher scores indicating higher independence),^27^ Hospital Anxiety and Depression Scale (HADS; scores ranged from 0 to 21, with higher scores indicating higher anxiety and depression),^28^ Pain screening tool (Pain, Enjoyment of Life and General Activity [PEG]; scores ranged from 0 to 10, with higher scores indicating higher pain),^29^ and the Patient-Reported Outcomes Measurement Information System (PROMIS) Sleep Disturbance short form 4a (scores ranged from 4 to 20, with higher scores indicating poor sleep).^30^ The care coordinator, with input from the m-CCRP team, prepared and delivered the care plan to the patient within 2 weeks of the initial assessment. The care coordinator used recovery protocols targeting cognition, physical function, personal care, mobility, sleep disturbances, pain, depression, anxiety, agitation or aggression, delusions or hallucinations, stress and physical health, legal and financial needs, and medication adherence (eBox, eMethods in Supplement 2). The 12-month intervention period was designed to include ongoing coordinator contacts (every 2 weeks for the first 6 months and once per month for the last 6 months) with patients in-person or by telephone, and longitudinal symptom monitoring. During the COVID-19 pandemic, study protocols were modified with all interactions conducted via the telephone (eMethods in Supplement 2).

### Attention Control

The control group received a guide for ICU survivors containing information on community resources and caregiver education (eMethods in Supplement 2). The control participants were scheduled to receive scripted telephone calls by a care coordinator assistant every 2 weeks for the first 6 months and once a month for the last 6 months of study duration.

### Process Measures

We collected the number of nurse contacts and the number of protocols initiated per patient in the m-CCRP group and number of telephone calls in the control group. Consults relevant to post-ICU care, orders for medication prescriptions, and services potentially rendered due to care coordination were captured through electronic medical records.

### Outcome Measures

Trained research assistants performed blinded baseline and outcome assessments at 0, 3, 6, and 12 months. The primary outcome was patient’s health-related QOL assessed through the Medical Outcomes Study 36-Item Short Form Health Survey (SF-36) at 12 months.^31^ The 8 components (physical functioning, role-physical, bodily pain, general health, vitality, social functioning, role-emotional, and mental health) were aggregated into a Physical Component Summary (PCS) and a Mental Component Summary (MCS), with scores on each subscale ranging from 0-100, higher scores indicating better health status.^31^ A difference of more than 2 points is considered clinically meaningful for both the PCS and MCS subscales of the SF-36.^31^ Secondary outcomes included depression and anxiety symptoms assessed through the Patient Health Questionnaire-9 (PHQ-9; scores ranged from 0 to 27, with higher scores indicating greater severity of depression)^32^ and Generalized Anxiety Disorder Scale (GAD-7; scores ranged from 0 to 21, with higher scores indicating greater anxiety),^33^ and cognition assessed via the Repeatable Battery for the Assessment of Neuropsychological Status (RBANS; scores ranged from 40-160, with higher scores indicating better cognition).^34^ Differences of at least 3 points for the PHQ-9 and GAD-7 and of 1.96 points for the RBANS index score are considered clinically meaningful.^35,36^ Initially, physical performance was assessed by the Short Physical Performance Battery,^37^ but during the COVID-19 pandemic, visitation restrictions prohibited physical assessments, and cognitive assessments were collected by telephone. Indiana Health Information Exchange provided data for emergency department (ED) visits and rehospitalizations.

### Other Data Collection

We collected demographics (age, sex, race and ethnicity, educational level), illness severity (Acute Physiology and Chronic Health Evaluation [APACHE II; scores ranged from 0 to 71, with higher scores indicating higher illness severity]),^38^ comorbidities (Charlson Comorbidity Index; scores ranged from 0 to 37, with higher scores indicating higher illness comorbidities),^39^ medications, cognition prior to admission (Informant Questionnaire on Cognitive Decline in the Elderly [IQCODE]; scores ranged from 1 to 5, with higher scores indicating cognitive impairment),^40^ activities of daily living (Katz scale; scores ranged from 0 to 6, with higher scores indicating higher independence) and instrumental activities of daily living (Lawton scale; scores ranged from 0 to 8, with higher scores indicating higher independence),^26,27^ duration of mechanical ventilation, and ICU and hospital lengths of stay. Self-reported race and ethnicity categories included African American, White, and other (including Asian, American Indian or Alaska Native, and multiple races).

### Statistical Analysis

For the sample size and power estimation, the trial planned to randomize 620 participants (400 completing the 12-month evaluation) with 80% power to detect an effect size of 0.28 SD in primary outcome measures of PCS or MCS at the end of the 12-month follow-up. With trial interruption due to the COVID-19 pandemic, a revised power estimation was conducted and approved by the Data and Safety Monitoring Board. The current enrollment of 466 patients would be able to detect an effect size of 0.32 SD with 80% power.

Intention-to-treat analysis was used to compare outcome measures between the 2 groups. Mixed-effects models were used with longitudinal SF-36 PCS, MCS, PHQ-9, GAD-7, and RBANS scores collected at baseline, 3, 6, and 12 months as the outcome measures; group, time, and interactions between group and time as independent variables were assessed while adjusting for hospital, discharge location (stratification variables), and APACHE II scores that were significantly different between the 2 groups. An unstructured variance-covariance matrix was used in the mixed-effects models to adjust for potential correlations among outcome measures obtained from the same individual over time. A significant interaction between group and time would indicate differences in changes of outcome measures over time between the 2 groups. Post hoc comparisons using linear contrasts were generated to compare changes in outcome measures from baseline between the 2 groups at each postrandomization time point. Rates of ED visit, rehospitalization, and mortality were compared using the χ^2^ test.

We compared baseline characteristics of participants with complete 12-month follow-up data, incomplete follow-up data, or who died, by randomization status using an analysis of variance for continuous variables and the χ^2^ test for categorical variables. Since mixed-effects models provide unbiased estimates when predictors associated with missingness are included in the models under the missing at random assumption,^41^ we conducted additional mixed-effects models for longitudinal outcome measures, adjusting for baseline variables that were different between participants who completed the 12-month follow-up and participants with missing 12-month data.

We used time-to-event models to analyze time from enrollment to ED visits, hospital readmissions, and death, with group as the independent variable while adjusting for site, stratification variables, age, APACHE II score, Charlson Comorbidity Index, and ICU length of stay. Patients followed up to 12 months without experiencing an event had their event time censored at 12 months; patients who died or were unavailable for follow-up had their time to ED visit or rehospitalization censored at the time of death or date of last contact. Recurring ED visits or rehospitalizations were modeled using the Andersen-Gill model for multiple events using elapsed times with robust variance. Additional analyses are included in the eMethods in Supplement 2. All analyses were conducted using SAS, version 9.4 software (SAS Institute Inc). A 2-sided value of *P* < .05 was considered statistically significant.

## Results

### Participant Characteristics

**Figure 1.**
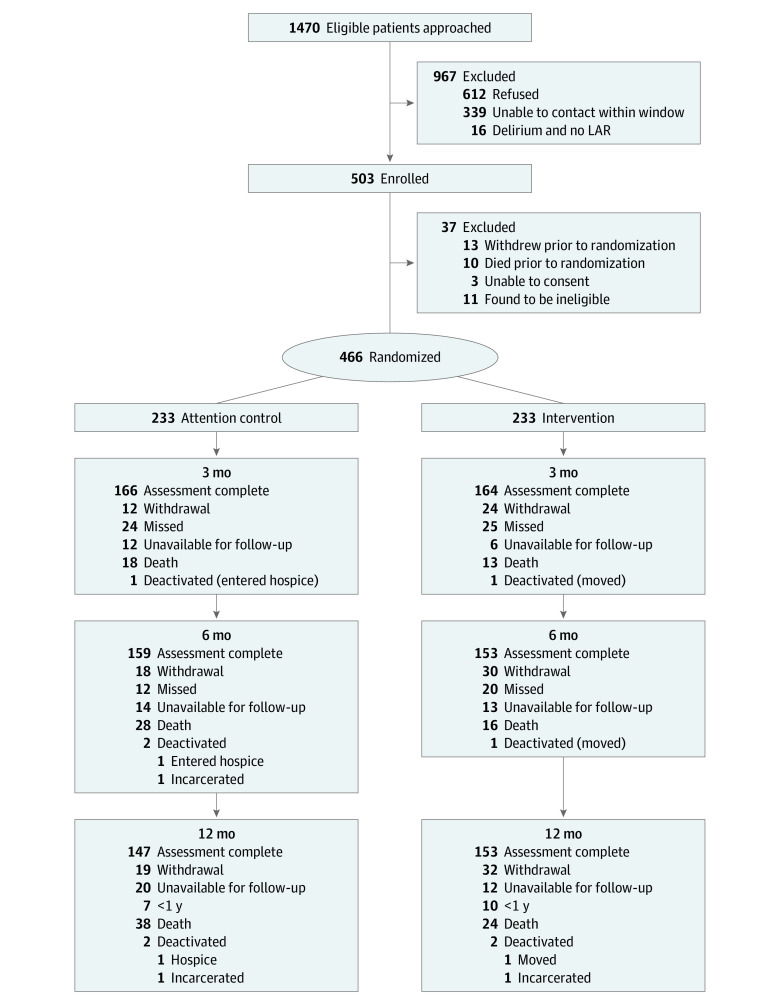
The m-CCRP CONSORT Flow Diagram LAR represents legally authorized representative; m-CCRP, Mobile Critical Care Recovery Program.

From 2017 to 2022, 1470 ARF survivors were approached, 503 were enrolled, 466 completed baseline assessments, and 233 were randomly assigned to m-CCRP and 233 to control (Figure 1). In total, 27 informal caregivers provided consent to be enrolled in the study, although 182 ARF survivors (78.1%) had caregiver support in the intervention group. Sixty-two patients died, 51 patients withdrew consent, 32 patients were unavailable for follow-up, and 17 patients received 6 months of follow-up toward the end of study due to funding limitation. The mean (SD) age of the enrolled study participants was 56.1 (14.4) years, 250 (53.6%) were female, 216 (46.4%) were male, 172 (36.9%) were African American, and 280 (60.1%) were White, 7 were categorized as other (individual numbers are not provided to prevent identification), and the race and ethnicity of 13 patients was unknown. Patients had a median (IQR) APACHE-II score of 25 (19-31) and a median (IQR) Charlson Comorbidity index of 2 (1-3) (Table 1).

**Table 1.** Patients’ Baseline Characteristics by Randomization Groups

Characteristic	Patients, No. (%)	
	m-CCRP (n = 233)	Control (n = 233)
Age, mean (SD), y	55.2 (14.3)	56.9 (14.4)
Sex		
Female	125 (53.6)	125 (53.6)
Male	108 (46.4)	108 (46.4)
Race		
African American	94 (40.3)	78 (33.5)
White	134 (57.5)	146 (62.7)
Other^a^	3 (1.3)	4 (1.7)
Unknown	2 (0.9)	5 (2.2)
Ethnicity		
Hispanic	0	5 (2.2)
Educational level		
0-11 y	33 (14.7)	26 (11.5)
12 y	87 (38.8)	87 (38.5)
Vocational school	4 (1.8)	8 (3.5)
Some college	35 (15.6)	45 (19.9)
Associate’s degree	26 (11.6)	17 (7.5)
Bachelor’s degree	24 (10.7)	23 (10.2)
Master’s or doctorate degree	15 (6.7)	20 (8.8)
APACHE-II score^b^	24 (18-31)	25 (20-31)
IQCODE score^c^	3.1 (0.4)	3.1 (0.5)
Functional status prior to illness		
Activities of daily living (Katz Scale)^d^	6 (6-6)	6 (6-6)
Instrumental activities of daily living (Lawton Scale)^e^	8 (6-8)	8 (6-8)
Comorbidities		
Charlson Comorbidity Index^f^	2 (1-3)	2 (1-3)
Depression^g^	82 (35.2)	86 (36.9)
Anxiety^g^	69 (29.6)	65 (27.9)
Cognitive impairment^g^	4 (1.7)	8 (3.4)
Insurance		
Private	61 (26.8)	53 (23.1)
Medicaid or Medicare	43 (18.9)	44 (19.2)
Medicaid	34 (14.9)	34 (14.8)
Medicare or private	30 (13.2)	29 (12.7)
Medicare	24 (10.5)	38 (16.6)
Other	27 (11.8)	19 (8.3)
None	9 (4.0)	12 (5.2)
Reason for intensive care unit admission^h^		
Respiratory	233 (100)	233 (100)
Acute kidney failure	84 (36.1)	81 (34.8)
Surgical or trauma^i^	55 (23.6)	46 (19.7)
Cardiovascular	39 (16.7)	46 (19.7)
Sepsis or septic shock	24 (10.3)	28 (12.0)
Neurological	10 (4.3)	15 (6.4)
Gastrointestinal	8 (3.4)	10 (4.3)
Other^j^	9 (3.9)	4 (1.7)
Etiology of acute respiratory failure^h^		
Postoperative^i^	83 (35.6)	72 (30.9)
Pneumonia	37 (15.9)	36 (15.5)
Cardiovascular	33 (14.2)	34 (14.6)
Sepsis or septic shock	31 (13.3)	26 (11.2)
Chronic obstructive pulmonary disease or asthma	15 (6.4)	22 (9.4)
Neurological	14 (6.0)	20 (8.6)
COVID-19	11 (4.7)	12 (5.1)
Aspiration	3 (1.3)	2 (0.9)
Other^k^	16 (6.9)	15 (6.4)
Shock requiring vasopressor	176 (75.5)	178 (76.4)
Respiratory clinical characteristic		
Pao_2_/Fio_2_ ratio, median (IQR)	148.3 (90.8-213.8)	158.0 (99.0-253.0)
ARDS with Pao_2_:Fio_2_ <200 mm Hg	109 of 156 (69.9)	111 of 171 (64.9)
Type of ventilatory support		
Invasive mechanical ventilation	224 (96.1)	225 (96.6)
Noninvasive positive pressure ventilation or heated humidified high flow nasal cannula	9 (3.9)	8 (3.4)
Service		
Medical ICU	136 (58.4)	148 (63.5)
Surgical ICU	97 (41.6)	85 (36.5)
Duration of mechanical ventilation, median (IQR), h	68.8 (40.0-134.9)	79.1 (40.7-129.8)
Length of ICU stay, median (IQR), d	9.0 (5.0-14.0)	9.0 (5.0-13.0)
Length of hospital stay, median (IQR), d	15.0 (10.0-24.0)	15.0 (10.0-20.0)
Discharge location		
Home	141 (60.5)	135 (57.9)
Inpatient rehabilitation	52 (22.3)	50 (21.5)
Acute rehabilitation	22 (9.4)	23 (9.9)
Long-term acute care	11 (4.7)	12 (5.2)
Skilled nursing facility	6 (2.6)	8 (3.4)
Other	1 (0.4)	5 (2.2)

Abbreviations: APACHE, Acute Physiology and Chronic Health evaluation; ARDS, acute respiratory distress syndrome; IQCODE, Informant Questionnaire on Cognitive Decline in the Elderly; ICU, intensive care unit; m-CCRP, Mobile Critical Care Recovery Program; Pao_2_/Fio_2_, arterial partial pressure of oxygen to fraction in inspired oxygen ratio.

^a^ Other race includes Asian, American Indian or Alaska Native, and multiple races.

^b^ APACHE-II scores range from 0 to 71, with higher scores indicating higher illness severity.

^c^ IQCODE scores range from 1 to 5, with higher scores indicating cognitive impairment.

^d^ Katz scale scores range from 0 to 6, with higher scores indicating higher independence.

^e^ Lawton scale scores range from 0 to 8 with higher scores indicating higher independence.

^f^ Charlson Comorbidity Index scores range from 0 to 37, with higher scores indicating higher illness comorbidities.

^g^ Diagnoses based on prehospitalization *International Statistical Classification of Diseases and Related Health Problems, Tenth Revision,* diagnoses.

^h^ Reasons are not mutually exclusive.

^i^ Includes general surgery, trauma, transplant, and acute burn.

^j^ Includes airway obstruction, metabolic, or endocrine causes.

^k^ Other includes angioedema, pulmonary hemorrhage, pneumothorax, and upper airway obstruction.

### Patient Outcomes

**Figure 2.**
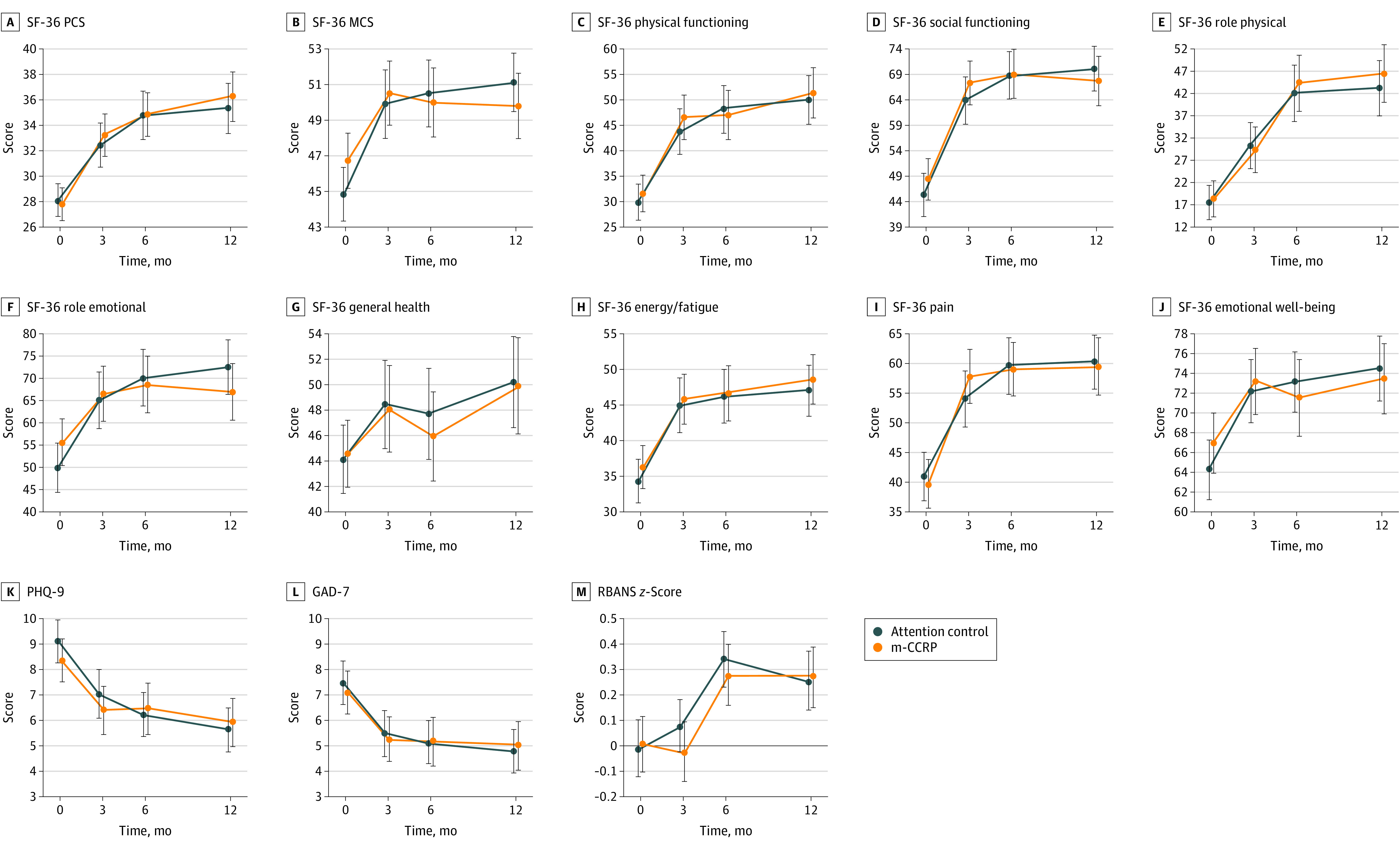
Quality of Life, Depression, Anxiety, and Cognitive Outcomes from Baseline to 12 Months GAD-7 indicates 7-Item Generalized Anxiety Disorder (range, 0-21; higher scores indicate greater anxiety); MCS, Mental Component Summary of SF-36; PCS, Physical Component Summary of SF-36; PHQ-9 Patient Health Questionnaire-9 (range, 0-27; higher scores indicate greater severity of depression); RBANS, Repeatable Battery for the Assessment of Neuropsychological Status (range, 40-160, higher scores indicate better cognition); and SF-36, 36-Item Short-Form Health Survey (each component score range is 0-100; higher scores indicate better health status). Data points represent mean scores; error bars, 95% CIs.

Patients showed significant disability at baseline as evidenced by their mean (SD) SF-36 PCS (m-CCRP, 27.78 [9.82]; control, 28.01 [9.89]) and MCS (m-CCRP, 46.74 [11.83]; control, 44.85 [11.43]) scores. During the 12-month intervention, no significant differences were observed in SF-36 PCS scores between the m-CCRP and control groups (mean difference in change from baseline: 1.61 [95% CI, −1.06 to 4.29]; *P* = .63]) or in MCS scores (mean difference in change: −2.50 [95% CI, −5.29 to 0.30]; *P* = .32)] (Figure 2; Table 2). The scores improved in both groups but indicated significant disability. There were no significant differences in the SF-36 subscale scores (Figure 2; eTable 1 in Supplement 2). A subgroup analysis based on race, sex, at least 65 years of age, APACHE II scores of 25 or higher, duration of mechanical ventilation of 80 hours or more, and ICU length of stay of at least 9 days did not show any differences in SF-36 PCS scores (eFigure 1 in Supplement 2). Control participants younger than 65 years and patients with duration of mechanical ventilation fewer than 80 hours had better MCS scores at 12 months (eFigure 1 in Supplement 2).

**Table 2.** Comparison of Patient Outcomes by Study Group Over Time

Outcome	Control		m-CCRP intervention		Estimated difference in change from baseline (95% CI)	*P* value
	No.	Mean (SD)	No.	Mean (SD)		
SF-36 Physical Component Summary^a^						.63
Baseline	222	28.01 (9.89)	221	27.78 (9.82)	NA	NA
3 mo	164	32.40 (11.28)	164	33.14 (1.93)	1.39 (−0.92 to 3.69)	.24
6 mo	159	34.73 (12.32)	151	34.78 (11.24)	1.40 (−1.18 to 3.97)	.29
12 mo	146	35.29 (12.20)	153	36.22 (12.55)	1.61 (−1.06 to 4.29)	.24
SF-36 Mental Component Summary^a^						.32
Baseline	222	44.85 (11.43)	221	46.74 (11.83)	NA	NA
3 mo	164	49.90 (12.46)	164	5.49 (11.85)	−0.90 (−3.63 to 1.83)	.52
6 mo	159	50.50 (12.05)	151	5.00 (12.13)	−1.46 (−4.46 to 1.54)	.34
12 mo	146	51.12 (10.23)	153	49.81 (11.75)	−2.50 (−5.29 to 0.30)	.08
Patient Health Questionnaire-9^b^						.61
Baseline	202	9.10 (6.19)	205	8.33 (6.22)	NA	NA
3 mo	164	7.02 (6.38)	161	6.41 (6.17)	−0.06 (−1.37 to 1.25)	.93
6 mo	157	6.22 (5.68)	150	6.47 (6.41)	0.45 (−0.96 to 1.86)	.53
12 mo	147	5.66 (5.37)	153	5.94 (6.13)	0.53 (−0.80 to 1.86)	.43
Generalized Anxiety Disorder-7^c^						.93
Baseline	203	7.47 (6.24)	207	7.11 (6.18)	NA	NA
3 mo	162	5.50 (5.85)	161	5.29 (5.71)	0.07 (−1.16 to 1.31)	.91
6 mo	157	5.15 (5.36)	150	5.19 (5.85)	0.13 (−1.22 to 1.48)	.85
12 mo	147	4.82 (5.25)	153	5.04 (6.08)	0.37 (−0.94 to 1.67)	.58
RBANS 7-Item, *z* score^d^						.64
Baseline	163	−0.01 (0.73)	176	0.01 (0.74)	NA	NA
3 mo	146	0.08 (0.66)	141	−0.02 (0.71)	−0.05 (−0.18 to 0.08)	.49
6 mo	142	0.34 (0.68)	135	0.28 (0.71)	−0.03 (−0.16 to 0.11)	.69
12 mo	133	0.26 (0.68)	129	0.27 (0.70)	0.02 (−0.11 to 0.16)	.73

Abbreviations: RBANS, Repeatable Battery for the Assessment of Neuropsychological Status; m-CCRP, Mobile Critical Care Recovery Program; SF-36, 36-Item Short-Form Health Survey.

^a^ The SF-36 consists of 8 domains that yield 2 summary scores: physical component score and mental component score, each of which range from 0 to 100, with higher scores indicating better health status, and a difference of 2 or more points is considered clinically meaningful for both subscales.

^b^ Scores range from 0 to 27, with higher scores indicating greater severity of depression.

^c^ Scores range from 0 to 21, with higher scores greater anxiety.

^d^ The overall RBANS *z* score is the mean of the 7-item (RBANS subscale) *z* scores; scores range from 40 to 160, with higher scores indicating better cognition.

Baseline mean (SD) PHQ-9 scores showed mild depression (m-CCRP, 8.33 [6.22]; control, 9.10 [6.19]). The mean difference in change in PHQ-9 scores did not differ between intervention and control groups at 3 months (−0.06 [95% CI, −1.37 to 1.25]), 6 months (0.45 [95% CI, −0.96 to 1.86]), and 12 months (0.53 [95% CI, −0.80 to 1.86]) (*P* = .61) (Table 2; Figure 2) The PHQ-9 scores at 12 months remained in the mild depression range (m-CCRP, 5.94 [6.13]; control, 5.66 [5.37]). At baseline, the mean (SD) GAD-7 scores showed mild anxiety in both the m-CCRP (7.11 [6.18]) and control (7.47 [6.24]) groups. At 12 months, the mean difference in change in GAD-7 scores did not vary between groups (0.37 [95% CI, −0.94 to 1.67]; *P* = .93), with raw scores showing mild or minimal anxiety (Table 2; Figure 2).

The baseline RBANS total index scores representing global cognition were 1.5 SD below the population age-adjusted mean (SD) (100 [15]) in both groups (m-CCRP, 73.96 [15.05]; control, 75.44 [14.52]). The scores improved by 12 months but were still 1 SD below the population means (m-CCRP, 82.55 [15.83]; control 84.74 [12.18]). There were no differences in the mean change in RBANS index *z* scores for 12 months (0.02 [95% CI, −0.11 to 0.16]; *P* = .73 (Table 2; Figure 2). Individual RBANS domains scores did not differ between m-CCRP and control groups during the 12-month intervention period (eTable 2 in Supplement 2).

### Acute Care Use and Mortality

There were no differences in the number of ED visits between the 2 groups (m-CCRP, 93 [39.9%]; control, 81 [34.8%]; *P* = .25). The rates of rehospitalization were higher in the m-CCRP group (117 [50.2%]) compared with control (95 [40.8%]; *P* = .04), whereas the 12-month mortality rates were lower although not significantly (m-CCRP, 24 [10.3%]; control, 38 [16.3%]; *P* = .05). There were no significant differences in time to ED visits or death between the 2 groups (eTable 3A in Supplement 2). The time to rehospitalization did not differ by discharge location (eTable 3B in Supplement 2), nor did the rates of rehospitalizations and ED visits (eTable 3C in Supplement 2). The reasons for ED visits and rehospitalizations were not different between the m-CCRP and control groups (eTable 4 in Supplement 2).

### Processes of Care

The patients who received the intervention had a mean (SD) of 7.4 (7.2) nurse contacts per patient. Before the COVID-19 pandemic, of 1076 contacts, 754 (70.1%) were in-home and 322 (29.9%) were telephone-based, with 110 of 114 (96.5%) in-person first contact. During the pandemic, all contacts were telephone-based. The care coordinator initiated 1232 care protocols, with a mean (SD) of 5.3 (4.1) protocols per patient (Table 3). Exercise (63.1%), physical health (62.4%), mobility (59.6%), and sleep (65.3%) protocols were frequently initiated at home (eTable 5 in Supplement 2). The median (IQR) time to protocol initiation was shorter for exercise (27 [19-53] days), physical health (28 [18-66] days), mobility (28 [18-59] days), and sleep (27 [19-49] days) protocols (eTable 6 in Supplement 2). The HABC-M, MMSE, HADS, PEG, and sleep scores, used for longitudinal symptom monitoring among m-CCRP participants, showed improvements (eFigure 2 in Supplement 2). In the control group, a mean (SD) of 9.3 (5.8) telephone calls were made per patient. The number of referrals and rehabilitation consults did not differ between groups except for higher social work and mental care referrals and slightly but not significantly more anxiolytic prescriptions in the m-CCRP group (eTable 7 in Supplement 2). Mapping of outpatient services to m-CCRP protocols showed only stress and legal protocols resulting in more services (eTable 8 in Supplement 2).

**Table 3.** Process Measures for the Intervention and Attention Control Groups

Measure	No. (%)
**m-CCRP protocol (n = 233)**	
Protocols initiated	
Sleep disturbances	151 (64.8)
Exercise	149 (64.0)
Physical health	147 (63.1)
Mobility	139 (59.7)
Pain	123 (52.8)
Depression	103 (44.2)
Anxiety	103 (44.2)
Behavioral care	98 (42.1)
Stress	91 (39.1)
Cognition	67 (28.8)
Personal care	29 (12.4)
Medication adherence	13 (5.6)
Acute care reduction or delirium	11 (4.7)
Legal and financial	4 (1.7)
Communication	4 (1.7)
Care coordinator contacts, mean (SD), No.	
Total	7.4 (7.2)
Telephone	4.1 (5.8)
Face-to-face	3.2 (4.6)
**Attention control (n = 233)**	
No. of attention control calls, mean (SD)	9.3 (5.8)

Abbreviation: m-CCRP, Mobile Critical Care Recovery Program.

### Supplementary Analyses

Analyses of participants 65 years or older, participants with PHQ-9 scores of 10 or higher, and a dose-response based on the number of care coordinator contacts did not show any differences in outcomes between groups (eTables 9, 10, and 11 in Supplement 2). eTable 12 in Supplement 2 indicates that participants in the m-CCRP group with more than 25% reduction in HABC-M scores had better outcomes in some measures compared with controls. eTable 13 in Supplement 2 gives the results before and during the COVID-19 pandemic, with the m-CCRP group before the COVID-19 pandemic having worse SF-36 MCS scores than controls at 12 months. eTable 14 in Supplement 2 indicates no differences in intervention results by discharge location. eTable 15 in Supplement 2 indicates that study participants who died had longer durations of mechanical ventilation and longer ICU stays. Adjusting for duration of mechanical ventilation and ICU length of stay in mixed-effects models did not change the study results (eTable 16 in Supplement 2). Hence, accounting for missing data is unlikely to change the results for longitudinal outcomes. In a per-protocol analysis (patients receiving at least 50% of the m-CCRP dose), there were no significant differences in outcomes between the 2 groups (eTable 17 in Supplement 2).

## Discussion

To our knowledge, the m-CCRP represents the first large randomized clinical trial in the US testing an interdisciplinary nurse-led, clinician-supported intervention to improve health-related outcomes in ARF survivors. The 12-month m-CCRP intervention did not significantly improve the ARF survivors’ QOL. The mental and physical components assessed through the SF-36 improved in both the intervention and control groups, as did the individual SF-36 subscales. Those findings are in contrast with a prior geriatric care program by members of our team in which an advanced practice nurse and social worker supported by an interdisciplinary geriatrics team significantly improved the QOL of low-income older adults.^16^ We cannot exclude the possibility of a clinically meaningful effect as the 95% CI for the difference in the change between the m-CCRP and control groups included clinically meaningful differences of at least 2 points for the SF-36 PCS and MCS scores. The difference in change was 1.61 (95% CI, −1.06 to 4.29) for the PCS, demonstrating a potential benefit of our intervention on physical QOL, whereas the difference in change for the SF-36 MCS was −2.50 (95% CI, −5.29 to 0.30), indicating potential worsening. Our findings are similar to nurse-led ICU follow-up programs in the United Kingdom and Denmark, which did not improve QOL over a year after ICU discharge.^42,43^ Similarly, a comprehensive program of primary care providers and patients’ training, care coordination by nurses, and decision support for primary care providers by consulting physicians did not improve the QOL of sepsis survivors.^44^

We expected the m-CCRP to be efficacious for mood symptoms, given the aggressive delivery of care coordinator-led problem-solving therapy, antidepressant recommendations, and use of the HABC-M to tailor our intervention. Additionally, the m-CCRP was modeled on our prior programs for late life depression in the primary care setting, which showed better than 50% reduction in depressive symptoms at 12 months compared with usual care, results not replicated in the present trial.^15^ The lack of efficacy here could be due to the differences in populations (ie, primary care patients and ICU survivors). The 95% CI of estimated difference in change between groups did not include the clinically meaningful change score of 3 for the PHQ-9 and GAD-7, excluding benefit or harm. The absence of change in mood symptoms contrasts with the clinical worsening of SF-36 MCS scores, potentially showing that mood is not the primary mediator. The reasons behind the worsening of MCS scores in the m-CCRP group are unclear, although the control group started with lower baseline MCS scores with a higher potential for change. Determining the reasons will require exploration in future studies, preferentially through a qualitative approach. Our negative results are similar to a coping skills training program focused on mechanically ventilated patients and family members’ psychological distress, in which 6 weekly telephone-based coping sessions did not improve anxiety and depressive symptoms at 3 and 6 months.^45^ Similarly, a nurse-led program did not improve anxiety and depression symptoms in ICU survivors at 12 months.^43^ Although the m-CCRP group did not show improvements in cognition and physical function, these results should be considered in light of the COVID-19 pandemic. The cognition assessments and m-CCRP intervention were conducted via the telephone during the pandemic, resulting in a shorter cognitive battery, potentially increasing measurement errors and decreasing validity, and a lower in-person intervention dose. The physical function could not be assessed due to visitation restrictions, and outcomes were patient reported. Future trials should focus on delivering cognitive and physical interventions with high fidelity and in-person cognition and performance based functional measures.

The prior collaborative care models in geriatric populations with chronic medical conditions used by members of our team showed improvements in QOL,^16^ behavioral symptoms,^14^ and depression.^15^ The m-CCRP could not improve similar outcomes, despite having a similar care coordinator–based intervention, although with notable differences. In all prior programs the coordinator was integrated within the primary care practice. The m-CCRP coordinator worked with the primary care providers but was not embedded in the office. All prior trials^14,15,16^ included geriatric adults with chronic comorbidities, with different disease trajectories compared with acute illness survivors. All prior trials^14,15,16^ compared the intervention with usual care, whereas we compared m-CCRP with an attention control, which could have diluted our findings. Those previous trials achieved a higher number of nurse visits, although our dose response analysis did not identify a relationship between visits and outcomes. Finally, the m-CCRP care coordination did not result in a meaningful increase in referrals to relevant rehabilitation services or an increase in prescriptions for antidepressants, anxiolytics, and analgesics.

The m-CCRP targeted a patient population similar to other studies assessing acute respiratory distress syndrome^46,47,48,49^ with high severity of illness, multiple comorbidities,^46,47,49^ similar percentage (67%) of moderate to severe illness as defined by the arterial partial pressure of oxygen to fraction in inspired oxygen ratio lower than 200,^48^ but with a shorter duration of mechanical ventilation and ICU length of stay and lower sepsis diagnoses.^46,47,48,49^ Compared with participants in an observational study of acute respiratory distress syndrome survivors,^2^ m-CCRP participants in the present study had lower physical functioning, pain, general health, and social functioning scores, but higher vitality and role emotional scores and similar emotional well-being scores during 12 months. Patients in the m-CCRP group experienced higher rehospitalizations in contrast with a nurse-based 30-day transitional care program for sepsis survivors.^50^ That program consisting of medication review, symptom and comorbidity monitoring, and palliative care consults was able to reduce the composite outcome of rehospitalizations and mortality at 12 months.^50^ The discrepancy between their findings and the present trial could be due to the smaller number of sepsis survivors in the present cohort and a lower intervention dose within 30 days after discharge. We also included all ARF survivors without assessing rehospitalization or mortality risk. Future ICU recovery models will benefit from in-hospital risk stratification to identify patients at high risk for post-ICU complications and by implementing early postdischarge transitional care, incorporating both in-person and telehealth elements with input from relevant subspecialty disciplines.

### Limitations and Strengths

This study has limitations. We could not achieve our projected sample size due to the COVID-19 pandemic, had high attrition resulting in missing data, had abridged telephone collection for the cognition battery, and relied on patient reports for the physical function data. The strengths of this study were that we recruited a diverse sample (>50% female, 36.9% Black, and 60.1% White), tested a comprehensive intervention focused on multiple facets of ICU recovery, compared the intervention with a attention control recommended for behavior-based studies, and measured a broad range of validated, patient-oriented outcomes relevant to critical illness survivors in a concealed manner.

## Conclusions

This randomized clinical trial found that a care coordinator–led ICU recovery program supported by an interdisciplinary group of clinicians did not improve health outcomes during 12 months for ARF survivors. However, potential clinically important effects could not be ruled out, as the 95% CI of difference in the change between the m-CCRP and control groups included the estimate of meaningful clinical difference. Further research to identify specific patient groups who could derive benefit from more intensive in-home interventions may allow for improved recovery in survivors of critical illness.

## Notes

Supplement 1.
Trial Protocol

Supplement 2. **eMethods.** Methods, Intervention, Effect of COVID-19 on Study Operations, m-CCRP Care Protocols and Handouts, and Attention Control ICU Survivor Guide **eBox.** m-CCRP Care Coordinator Services **eTable 1.** m-CCRP Intervention Effects on SF-36 Subscales **eTable 2.** m-CCRP Intervention Effects on Repeatable Battery for the Assessment of Neuropsychological Status (RBANS) Subscales **eTable 3A.** Time to Emergency Department Visit, Re-Hospitalization, and Death Between m-CCRP Intervention and Control groups. **eTable 3B.** Time to Inpatient Hospitalization by Discharge Location Between m-CCRP Intervention and Control groups. **eTable 3C.** Comparison of Emergency Department Visits and Re-hospitalizations Between Patients Discharged to Home Versus Facility in Both Control and m-CCRP Groups **eTable 4A.** Emergency Department Diagnoses and Reasons at the Encounter Level **eTable 4B.** Hospital Readmission Diagnoses and Reasons at the Encounter Level **eTable 5.** Initiation of m-CCRP Protocols by Discharge Status **eTable 6.** Days From Discharge to Implementation of m-CCRP Protocols **eTable 7.** Comparison of Outpatient Orders and Medication Orders Between m-CCRP and Control **eTable 8.** Comparison of Outpatient Care Related to Initiated Protocols Between m-CCRP and Control **eTable 9.** Comparison of Study Outcomes Between Study Groups in Participants ≥65 Years of Age **eTable 10.** Comparison of Study Outcomes Between Study Groups in Participants With Baseline PHQ-9 ≥10 **eTable 11.** Comparison of Study Outcomes by Number of m-CCRP Care Coordinator-Patient Contacts **eTable 12.** Comparison of Study Outcomes When the m-CCRP Group is Divided into Those With >25% Reduction in Healthy Aging Brain Care Monitor (HABC-M Self Report) Symptom Scores and Those With No Reduction. **eTable 13.** Effect of m-CCRP Intervention on Patient Outcomes Pre and During COVID-19 Pandemic **eTable 14.** Effect of m-CCRP Intervention on Patient Outcomes by Discharge Status **eTable 15.** Comparison of Patient Baseline Characteristics by Completion Status at 12 Months **eTable 16.** Comparison of Patient Outcomes by Study Groups Over Time Including Adjustment for ICU Length of Stay and Mechanical Ventilation Duration **eTable 17.** Comparison of Patient Outcomes by Study Groups Over Time for m-CCRP Patients With at Least 9 Contacts (Per Protocol Analysis) **eFigure 1.** m-CCRP Intervention Results Based on Selected Sub-Group Analyses **eFigure 2.** Changes in Intervention Measures in the m-CCRP Group

Supplement 3.
Data Sharing Statement
