# Supplementary material for: Mobile Critical Care Recovery Program for Survivors of Acute Respiratory Failure: A Randomized Clinical Trial
Source: JAMA Netw Open. 2024 Jan 30;7(1):e2353158. doi: 10.1001/jamanetworkopen.2023.53158 (PMC10828910; doi:10.1001/jamanetworkopen.2023.53158)
Supplement: Supplement 1. — Trial Protocol [file jamanetwopen-e2353158-s001.pdf]

NHLBI Health Information Center
P.O. Box 30105
Bethesda, MD 20824-0105

Mobile Critical Care Recovery Program (m-CCRP) Protocol

Babar Khan, MD, MS
Research Scientist, IU Center for Aging Research
Assistant Professor of Medicine, IU School of Medicine
Division of Pulmonary/Critical Care

Table of Contents:

1.0 Background
2.0 Rationale and Specific Aims
3.0 Inclusion/Exclusion Criteria
4.0 Enrollment/Randomization
5.0 Study Procedures
6.0 Reporting of Adverse Events or Unanticipated Problems involving Risk to Participants or Others
7.0 Study Withdrawal/Discontinuation
8.0 Statistical Considerations
9.0 Privacy/Confidentiality Issues
10.0 Follow-up and Record Retention
11.0 References

1. Background

Acute Respiratory Failure (ARF) and Post-intensive Care Syndrome (PICS):
As the population ages and as advances in health care technology continue;[49] the US now has millions of ARF survivors.[50] The ARF survivors by virtue of their illness severity, physiologic derangements, procedural and pharmacologic interventions, mechanical ventilation, and delirium among others are exposed to extreme stressors. These stressors predispose survivors to physical, cognitive and psychological morbidity with a negative impact on the overall quality of life (QOL).[11-19,51-54] Recently these impairments are characterized under a unifying diagnosis of "post-intensive care syndrome (PICS)".[28] *PICS is defined as a cluster of debilitating symptoms characterized by physical impairment, cognitive impairment, and symptoms of depression and anxiety. It is experienced by greater than 50% of ARF survivors, impairs recovery after hospital discharge and could persist for years.*[12,28,36]

After discharge from the ICU, PICS patients are referred back to their primary care providers who are ill prepared and inadequately resourced to care for them. The ensuing inadequate care affects PICS patients' health related QOL, leads to long-term disability, and impedes return to previous roles (e.g. < 50% of survivors return to work).[55-60] At present, there are few care models to guide healthcare providers to deliver excellent longitudinal care to ARF survivors. *A model to provide specialized and coordinated recovery care for ARF survivors with physical, psychological and cognitive impairments beginning at the time of discharge could fill the current gap and increase the likelihood of full functional recovery.*

Current state of literature on recovery services for ARF survivors:
There are guidelines for post-ICU care,[23,61] but there is little supporting evidence to recommend their implementation on a wide spread scale. A limited number of randomized trials have been conducted among ICU survivors with conflicting results. One randomized controlled trial evaluating the effects of a self-directed manual of exercises, psychological advice, and smoking cessation demonstrated improved physical function-related QOL, higher smoking cessation rates, and a trend towards decreased depression.[62] A small trial (n: 21) testing a multicomponent program of in-home cognitive and physical rehabilitation showed improvements in cognitive and physical outcomes at 3 months.[63] However, another randomized trial of home-based rehabilitation for 8 weeks demonstrated no improvement in physical function-related QOL.[64] Similarly, the PRaCTICal trial of a nurse-led outpatient follow-up program for 3 months after an ICU stay showed no improvements in QOL, anxiety, or depression.[65] The recently published RECOVER randomized trial testing a mobility, exercise and nutrition intervention lasting from ICU discharge to hospital discharge failed to show improvement in mobility and QOL at 3 months. The QOL scores remained static from 3 to 12 months.[66] The studies at present do not delineate a clear path to manage the multiple comorbidities of PICS. A number of these studies were performed outside the US, had small sample sizes, and failed to address the multi-component nature of PICS. Studies with adequate sample sizes tested interventions for short durations thereby limiting the exposure to the intervention.[64-66] Similar gaps were identified in proceedings from the Society of Critical Care Medicine stakeholders' conference.[28] *We plan to overcome these limitations by designing an*

interdisciplinary team-based intervention led by a care coordinator for a duration of 12 months. This approach is based on our prior successful experiences in using collaborative care model to manage the complex biopsychosocial needs of vulnerable older adults suffering from psychological, cognitive, and functional impairments.[35,67,68]

Making a Case for a Collaborative Critical Care Model to enhance Recovery of ARF Survivors: The Institute of Medicine has prioritized the development and implementation of new care coordination programs to improve patient-centered and interdisciplinary health care delivery.[32] Although there are community resources, rehabilitation centers, and in-home services available to ARF survivors, these services are accessed at the discretion of multiple clinical disciplines.[29,31] The fragmented nature of our current health care system does not integrate the multitude of services to promote rapid recovery of ARF survivors.[29,31] The disjointed process of recovery support programs and healthcare system complexity places unnecessary burdens on vulnerable ARF survivors.[69] The ARF survivors suffer from complex and multiple health related problems that require care coordination. *Access to a care coordinator empowered with the tools and support to integrate and connect essential resources has the potential to enhance the recovery of ARF survivors.*

In response to the Institute of Medicine call, and utilizing IUCAR's expertise in collaborative, care-coordinator based care, we have developed the *Mobile Critical Care Recovery Program (m-CCRP)* to provide comprehensive, collaborative and specialized care for ARF survivors with physical, psychological and cognitive impairment. The proposed randomized controlled trial will evaluate the efficacy of such a collaborative critical care recovery program in accomplishing the Institute of Healthcare Improvement's triple aims of better health, better care, at lower cost.[70]

2. Rationale and Specific Aims

Five million Americans require admission to intensive care units (ICU) annually due to life-threatening illnesses,[1-3] and this number is expected to rise.[4] Two million of these ICU admissions are secondary to Acute Respiratory Failure (ARF) with more than half requiring mechanical ventilation.[5,6] Greater than 50% of the patients with ARF who survive their ICU stay suffer from long-term morbidity in the form of functional disability,[7-11] cognitive disability,[10-14] major or minor depression,[13-17] and anxiety.[14,17-19] These complications negatively impact the quality of life (QOL) of ARF survivors, interfere with their physical and emotional recovery, and lead to long-term disability with less than half of ARF survivors returning to the work force.[7,20-26] Ongoing care for ARF survivors has been estimated to cost $3.5 million per ARF survivor at 1 year.[27] This constellation of ICU sequelae with attendant morbidity has been designated as the *post-intensive care syndrome (PICS).*[28] There are community resources and rehabilitation services available to ARF survivors, but the current fragmented nature of our healthcare system is unable to integrate and coordinate care in order to provide the most meaningful recovery.[29-31]

The Institute of Medicine recommends the development of care coordination programs to deliver patient-centered and interdisciplinary-based medical care.[32] Indiana University Center for Aging Research (IUCAR) has over 20 years of experience in delivering interdisciplinary,

collaborative care through pragmatic interventions utilizing care coordinators.[33-36] Care coordinator delivery models have improved care of patients with dementia, depression, functional decline and PICS.[33-36] Our PICS clinic, the Critical Care Recovery Center (CCRC) was developed in 2011 to enhance cognitive, physical, and psychological recovery of ICU survivors. Since then, CCRC has provided care to over 200 survivors with a high burden of PICS (88% had cognitive impairment; 60% had depression).[36] CCRC showed feasibility and initial efficacy in managing PICS.[36] However given its traditional outpatient clinic structure, CCRC has limited physical access to ARF survivors, leading to delayed evaluation and an added travel burden on ARF survivors. *Building from the experience of the CCRC and our other successful care coordinator based programs; we now propose a mobile model of post-ICU collaborative care with greater access to enhance the recovery of ARF survivors.*

The overarching aim of the proposed program is to improve the QOL of patients who survived an episode of ARF by maximizing their cognitive, physical, and psychological recovery utilizing a mobile care coordinator. The care coordinator will bring the intervention to the patient irrespective of the patient's physical location and will be supported by an interdisciplinary team of a critical care physician, a health services scientist, an ICU nurse, and a psychologist, with input from other consultants as needed. In addition, dynamic feedback through process measurement tools and care coordination support software will inform the recovery process.[37-39] We propose to evaluate the efficacy of our collaborative care intervention termed the "*Mobile Critical Care Recovery Program (m-CCRP)*" through a randomized clinical trial among survivors of ARF by pursuing the following specific aims:

Primary Specific Aim:

To evaluate the efficacy of m-CCRP in improving the health related quality of life of ARF survivors compared to attention control at twelve months post hospital discharge.

Hypothesis: ARF survivors randomized to twelve months of m-CCRP will have higher scores on Physical Component Summary (PCS) and Mental Component Summary (MCS) of the Medical Outcomes Study 36-item short form (SF-36),[40-42] compared to those randomized to attention control at twelve months post discharge.

Secondary Specific Aim 1:

To evaluate the efficacy of m-CCRP in improving the cognitive, physical, and psychological function of ARF survivors compared to attention control at twelve months post hospital discharge.

Hypotheses: ARF survivors randomized into twelve months of m-CCRP will have higher composite cognitive scores as assessed by the Repeatable Battery for the Assessment of Neuropsychological Status (RBANS);[43] higher physical performance as measured by the Short Physical Performance Battery (SPPB);[44] and lower depression and anxiety symptoms as measured by the Patient Health Questionnaire–9 (PHQ-9),[45,46] and the Generalized Anxiety Disorder (GAD-7) scale[47,48] than those randomized to attention control at twelve months post hospital discharge.

Secondary Specific Aim 2:
To evaluate the efficacy of m-CCRP in reducing health-care utilization by ARF survivors as compared to attention control at twelve months post hospital discharge.

Hypothesis: ARF survivors randomized to m-CCRP will have lower health care utilization as measured by emergency department visits and re-hospitalizations, compared to those randomized to attention control.

3. Inclusion/Exclusion Criteria

Inclusion Criteria
- At least 18 years of age
- Hospitalized in the ICU
- Diagnosed with Acute Respiratory Failure (ARF) requiring greater than or equal to 24 hours of mechanical ventilation or noninvasive positive pressure ventilation or high flow nasal canula.
- Discharged to home, skilled nursing facility, sub-acute rehabilitation care, or long-term acute care
- English speaking
- Able to consent either in-person or through legally authorized representative
- Have access to a telephone

Exclusion Criteria
- Hospitalized to a regular non-ICU ward
- Diagnosis of cancer with life expectancy less than 1 year
- Admitted with ischemic or hemorrhagic cerebrovascular accident, traumatic brain injury, or undergoing neurosurgery
- History of dementing illnesses and other neurodegenerative disease such as Alzheimer’s disease, Parkinson’s disease, or vascular dementia (confirmed by EMR),or current prescription of anti-dementia medication, or ruled out by Functional Activities Questionnaire (FAQ) score defining dementia
- Unable to complete study questionnaire due to severe hearing loss
- Legally blind
- Pregnant (assessed by urine pregnancy test) or nursing
- Living outside the greater Indianapolis area
- Recent history of alcohol abuse > 5 drinks per day or substance abuse consistently over the last 3 months
- Status post tracheostomy and not eligible for a speaking valve
- Incarcerated at the time of study enrollment
- Schizophrenia or bipolar disorder (confirmed by EMR)
- Homelessness
- Illiterate

4. Enrollment/Randomization

Recruitment Targets:
ARF survivors will be recruited from three local tertiary care hospitals: 1) Indiana University Health Methodist, 2) University Hospital, and 3) Eskenazi Health. Recruitment from three hospitals with different demographics will increase the generalizability of our program in addition to achieving the target recruitment of enrolling 620 ARF survivors in our study.

Recruitment Procedures:
Research staff will check the electronic medical records of ICU patients daily for eligibility. Eligible individuals (those who meet inclusion criteria and do not meet any exclusion criteria) will be screened twice per day for delirium until ICU discharge using the CAM-ICU-7. Eligible patients' primary clinical teams will be approached to confirm the eligibility. Within 48 hours of anticipated discharge, patients and/or their legally authorized representatives will be approached for study participation and enrollment.

Enrollment:
Research assistants will obtain informed consent and authorization for release of the patient's medical records, and complete the Initial Evaluation around the time of discharge. This Initial Evaluation includes quality of life (SF-36), cognitive (RBANS, MOCA), physical function (SPPB), depression (PHQ-9), anxiety (GAD-7), and sleep (ISI-7) assessments. To estimate the treatment effect in key domains, a "step-down" battery, consisting of a subset of outcome measures, will be administered when a participant is not able to tolerate the full outcome assessments. This "step-down" battery may also be administered via phone if subject is unable to complete an in-person assessment.

Randomization:
Following the Initial Evaluation, ARF survivors will then be randomized in a 1:1 manner via a computer-generated randomization scheme into m-CCRP intervention or attention control group stratified by hospital

Dual Enrollment (OPTIONAL): Patients who are eligible for and enroll in Examining Neuropsychiatric Disorders in Survivors of COVID-19 Related Critical Illness (EN-COV; IRB #11444) may be dually enrolled in this project. EN-COV is a minimal risk study. These studies contain the same study procedures at hospital discharge and 6-month time points; therefore, data will be shared across these studies for any patient who dually enrolls to ensure study visits/assessments are not duplicated. The EN-COV study will be considered the "main study" in these instances, and will have all original source documents on file for dually enrolled subjects.

5. Study Procedures

Main Components of the Mobile Critical Care Recovery Program:
m-CCRP Care Coordinator: The m-CCRP revolves around the central figure of a care coordinator who will organize and align recovery resources. The care coordinator is a registered nurse whose scope of practice includes health coaching, case managing, community organizing, and nursing care provider. She has received additional education in the following areas: a)

recognizing the symptoms of PICS; monitoring the biopsychosocial needs and outcomes of ICU survivors; c) monitoring negative effects of medications; d) monitoring caregiver burden; e) delivering cognitive, physical, and psychological recovery protocols; f) enhancing the self-management capacity of patients and their informal caregivers; g) communicating and coordinating care with primary and specialty healthcare providers; h) coordinating community resources; and assisting patients and caregivers in developing symptom management skills.

Box 1: m-CCRP Care Coordinator Services

- Self-Management Skills Enhancement
- Support groups
- Continuous access to care coordinator via telephone support
- Medications Reconciliation
- Cognitive Protocol (Paper and computer based cognitive training focused on memory, attention, executive function)[84]
- Physical protocol (Multimodal exercises focused on seated aerobic and progressive resistance training)[85]
- Psychological protocol (Problem solving therapy, cognitive behavioral therapy, SSRIs per PCP)[67]
- Monitoring caregiver's emotional health
- Managing transitional care
- Managing acute care needs
- Root-Cause analysis of re-hospitalization or emergency room visits

The care coordinator will be responsible for conducting the home and facility visits; collaborating with the m-CCRP interdisciplinary team (see below); maintaining communication with the patient's primary care provider and specialists; conducting root-cause analysis for any preventable hospitalization or emergency department visit; implementing the individualized care plans; and monitoring the effectiveness of these care plans (Box 1). The care coordinator will be responsible for a new patient panel of 70 patients at any given time-point and will be supervised by the m-CCRP team.

m-CCRP interdisciplinary team:

The m-CCRP support team will consist of a critical care physician (Dr. Khan), a geriatrician with expertise in collaborative care (Dr. Boustani), an ICU collaborative care nurse (Dr. Lasiter), and a psychologist (Dr. Unverzagt). This team will meet weekly with the care coordinator. Drs. Khan and Boustani will be accessible to the coordinator through phone or pager at all times.

m-CCRP Tools:

m-CCRP Care Protocols: Care protocols have been developed by our interdisciplinary team and successfully utilized in the CCRC and m-CCRP pilot. These protocols address cognition, physical function, personal care, mobility, sleep disturbances, depression, anxiety, agitation or aggression, delusions or hallucinations, the caregiver's stress and physical health, driving safety, nutrition, and medication adherence (Box 1).

m-CCRP mobile access to patients: m-CCRP care coordinator will interact with patients in a variety of settings including primary care clinics; the patient's home; specialty clinics; hospitals; or rehabilitation centers. In contrast to our ICU survivor clinic, m-CCRP will physically go to patient rather than patient coming to m-CCRP.

m-CCRP Process Measures: The Care Coordination Support Software (EMR-ABC): We will utilize a modified version of the software that was developed for the ABC-Med Home[39,72] to meet the needs of the ARF survivors. This web-based care-coordination software includes the following functions: a) flexible and secure access to the platform from multiple locations and by various users; b) manual and web-based solutions to capture patient-centered cognitive, physical, and psychological symptoms; c) decision support to deliver personalized pharmacological and non-pharmacological care recovery protocols; d) tracking process of care coordination delivered by

the care coordination team; e) monitoring patients' and informal caregivers' responses to care recovery protocols; f) monitoring population-based outcomes to guide the overall program performance; g) integration capacity with other informatics tools such as the local electronic medical record and regional health information exchange; and h) an easy interface to move data from the software to analyzable datasets.

The Healthy Aging Brain Care (HABC) Monitor: The HABC Monitor[37,38] will be utilized to monitor the cognitive, physical, and psychological symptoms of patients and caregiver stress. The HABC monitor has 32 items tapping the previous four constructs (see appendix for instrument). While the total HABC Monitor score is helpful to measure change over time, each question also indicates a specific care area where help or coping strategies might be indicated. The HABC Monitor also includes questions on dangerous behaviors such as falls, home safety and automobile driving. When these cognitive, physical, or psychological symptoms will be identified, the m-CCRP team will work with the primary care clinician and other providers to begin initial pharmacological and non-pharmacological management. Hence the HABC-M will serve as a longitudinal monitoring tool that will guide the personalization of the m-CCRP care protocols to the needs of the patient at any point in time during recovery. The HABC-M will also provide dynamic feedback to allow for adaptation and customization of the intervention in real time by the care coordinator.

Study Flow:

Initial Evaluation: After enrolling subjects, the research assistants will perform blinded baseline quality of life (SF-36), cognitive (RBANS, MOCA) (Trails A and B), physical function (SPPB) 2 minute step test, post-traumatic stress syndrome questionnaire (PTSS-10), delirium experience questionnaire, depression (PHQ-9), anxiety (GAD-7), and sleep (ISI-7) assessments around the time of hospital discharge. After the randomization step, the care coordinator will contact the patients randomized into the intervention arm to start the m-CCRP intervention. Care coordinator will review hospital discharge and rehabilitation plan, identify the primary care physician, obtain the approval of the primary care to co-manage post ICU care of the eligible patients, and schedule a face-to-face visit with the patients at their place of discharge (home, sub-acute rehabilitation facility/skilled nursing facility or long-term acute care). This in-hospital step is important for relationship building and answering any concerns or queries.

The 12-month m-CCRP Intervention:

The First Visit: The care coordinator will conduct a face-to-face encounter at the patient's residence *within a week* post randomization. At the selected location, the coordinator will assess the patient's cognitive, physical, and psychological status using the Mini Mental State Examination (MMSE)[86]and/or the Montreal Cognitive Assessment (MoCA) Timed up and Go test,[87] Activities of daily living (Katz scale),[88] Instrumental activities of daily living (Lawton Scale),[89] Hospital Anxiety and Depression scale (HADS),[90] and the Healthy Aging Brain Care Monitors (HABC-M).[37,38] Caregiver stress will be noted with the HABC-M.[3]

The m-CCRP intervention instruments are different from the outcome assessments to ensure outcomes concealment from investigators. The coordinator will perform a community needs assessment for both the patient and the caregiver and will reconcile all prescribed and over the counter medications. The coordinator will make note of all scheduled and recommended appointments made at discharge with specialists and therapists. Initial and follow-up visits will be documented in the care coordination support software, the EMR-ABC.[39] (See above) Patients

and caregivers will be inquired of their personal recovery goals to be incorporated in the care plan (see below)

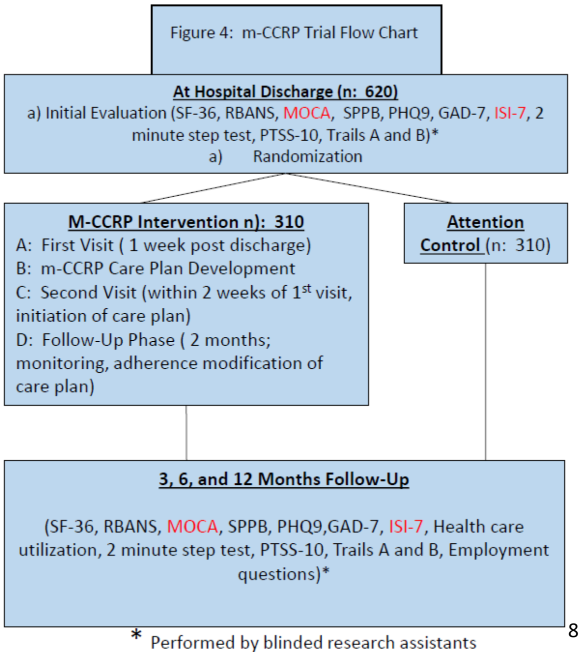

<u>Creation of an Individualized Care Plan:</u> Using the assessments and the information provided by the patient and the informal caregiver on the first visit, the coordinator will collaborate with the remainder of the m-CCRP team consisting of Drs. Khan, Boustani, Lasiter, and Unverzagt along with the primary care to finalize the individualized care plan. Patients that may benefit from specialty care will be recommended for specialty evaluation and co-management. If necessary, the patient will be referred for a more extensive cognitive and psychological evaluation at the local mental health practices. This decision would be jointly reached by the care coordinator, the m-CCRP team members, the patient, their caregiver, and the patient's primary care provider. Finally, a second face-to-face visit *within 2 weeks* of the first visit will be arranged.

<u>The Second Visit:</u> During the second visit, the coordinator will review the individualized recovery care plan with both the patient and the informal caregiver. This process will include a) understanding the diagnoses; b) process of monitoring the patient's recovery; c) implementation of the appropriate care recovery protocols; d) distribution and explanation of the corresponding educational recovery handouts (patient and informal caregiver); and e) connection to in-home services and community resources.

<u>The 12-month Interaction Period:</u> Follow-up includes a 12-month interaction period between the care coordinator, the patients and the informal caregivers via face-to-face home or clinic visit, phone contact, email, fax or mail. The minimum amount of contact during this time will be every two weeks for first 6 months and then once per month for last 6 months. During these interactions, the coordinator will answer any questions generated from previous visits; collect patient and caregiver's feedback; review and reconcile medications and discuss adherence; review specialists and therapists

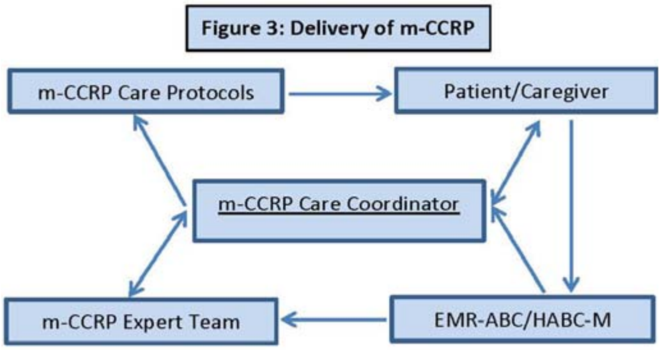

appointments and adherence to care plans; have the patient and the caregiver complete the HABC Monitor to trigger or modify the use of specific care recovery protocols; and facilitate the caregiver's access to appropriate community resources (Figure 3). Throughout the duration of the follow-up phase, the m-CCRP coordinator will continue to work with the patients, their caregivers, m-CCRP team, and the patient's primary care and specialists to monitor, implement, and revise as necessary the individualized recovery care plan based on the prior set recovery goals. The m-CCRP team and care coordinator will meet once per week to discuss new patients and monitoring patients' progress. If a patient develops an acute illness that requires hospitalization, the team activates the acute care transition phase where the coordinator contacts the hospital team and provides them with relevant information about the patient's cognitive, physical, and psychological symptoms as well as the patient's most updated medication list. Following a hospital discharge, the coordinator will conduct a home visit within 72 hours to reconcile medications and coordinate post discharge care plan. At the end of 12 months, every patient will be transitioned to receive full care by his or her primary care provider. Figure 3 demonstrates the central role of the care coordinator with feedback from patient and caregiver, the m-CCRP team and the m-CCRP tools of HABC-M and EMR-ABC (directions of arrows show information and care plan flow).

Outcomes Assessment at 3, 6, and 12 months*:*
Research assistants will perform blinded in-person outcomes assessments similar to the baseline assessment using the same instruments (SF-36, RBANS MOCA, Trails A and B, SPPB, 2 minute step test, PTSS-10, employment questions, PHQ-9, GAD-7, and ISI-7) at 3, 6, and 12 . Figure 4 summarizes the study flow. We will employ multiple techniques to ensure concealment of outcomes assessment.[91,92] Our research assistants will be trained not to inquire about study assignments. They will be conducting structured assessments that do not provide room for qualitative interviewing that should prevent unblinding. They will not be involved in study assignments and treatment administrations. Subjects will be instructed not to discuss their therapy with the research assistants. Loss of information could occur if subjects are unable to complete outcome assessments. To estimate the treatment effect in key domains, a "step-down" battery, consisting of a subset of outcome measures, will be administered when a participant is not able to tolerate the full outcome assessments. This "step-down" battery may also be administered via phone if subject is unable to complete an in-person assessment for the 3, 6 or 12 month outcomes.

Attention Control:
The study research coordinator will identify the primary care providers of patients in the attention control group and ensures appointment at the time of hospital discharge. The patients will receive a guide for ICU survivors containing phone numbers for relevant community resources, education on caregiver coping skills, and legal and financial advice. In addition, the attention control group will receive scripted phone calls by an independent care coordinator assistant not involved in any outcomes assessments. The calls frequency will be similar to the visit frequency of intervention arm (within one week post randomization) within two weeks post first phone call, afterwards twice per month for first six months, and once per month for the next six months). The phone calls will follow a script designed to ask the patients regarding their physical, cognitive, emotional and general wellbeing (see ICU survivors guide). Patients will be directed to the phone guide provided at the time of discharge. For those

without access to the guide, the relevant phone numbers will be provided on the call and a new guide mailed within 24 hours of the phone call. We have decided upon a phone based attention control after team discussions, literature review and by our prior clinical experience.[93-95] An in-home attention control was avoided due to the risk of diluting a care-coordinator home based intervention.

6. Reporting of Adverse Events or Unanticipated Problems

If a participant indicates suicide ideation on the PHQ-9, the patient's care team will be notified. Suicide ideation will also be reported to Dr. Khan and Dr. Sophia Wang (clinical psychiatrist working at IU Center for Aging Research) for additional follow up. Unanticipated problems will be reported according to Indiana University policy.

7. Study Withdrawal/Discontinuation

Participants who wish to withdraw from the study will notify the study team verbally during study interactions or via letter as outlined in the HIPAA authorization document.

8. Statistical Considerations

We will compare randomization results to pre-planned randomization schedule to ensure randomization integrity. To verify the comparability of the randomized groups, patients' baseline characteristics between the intervention and the usual care group will be compared using *t-tests* or Wilcoxon rank-sum tests for continuous variables and Chi-squared tests for categorical variables. All analyses will be conducted using SAS 9.4 software.

Self-reported quality of life outcomes:
Patient's health–related quality of life will be assessed using the Medical Outcome Study Short Form (SF-36). This scale has eight components (physical functioning, role-physical, bodily pain, general health, vitality, social functioning, role-emotional, and mental health) that are aggregated into a Physical Component Summary (PCS) and a Mental Component Summary (MCS).[40-42] Changes that differ between groups by 2 or more points on a scale of 0 to 100 have shown to be clinically meaningful.[42]

Cognitive Assessment:
We will use the Repeatable Battery for the Assessment of Neuropsychological Status (RBANS).[43] The RBANS was developed for the dual purposes of identifying and characterizing abnormal cognitive decline and a neuropsychological screening battery for adults aged 12-90 years. The entire battery takes 30 minutes to administer, and yields scaled scores for five cognitive domains (Immediate Memory, Visuospatial Construction, Language, Attention, and Delayed Memory). The RBANS has been tested and validated in neurologic, psychiatric, and healthy samples. RBANS has two versions and we will counterbalance for forms across the occasion of measurement to control for minor differences attributable to version.

Physical Performance:

Physical recovery will be assessed via the Short Physical Performance Battery (SPPB), a validated objective assessment.[44] The SPPB yields a performance score of 0-12 (0-4 poor, 5-7 intermediate, 8-12 good). A difference of 1 point indicates significant change in function.

Depression and Anxiety symptoms:

We will use the Patient Health Questionnaire–9 (PHQ-9)[45,46] and Generalized Anxiety Disorder Scale (GAD-7)[47,48] to determine the impact of the intervention on ARF survivors' mood and anxiety. The PHQ-9 is a nine-item depression scale with a total score from 0 to 27 and the GAD-7 is a seven-item anxiety scale with a total score from 0 to 21. Both of these scales are derived from the Patient Health Questionnaire, have good internal consistency, and test–retest reliability as well as convergent, construct, criterion, procedural and factorial validity for the diagnosis of major depression and general anxiety disorder.[45-48]

Acute Health Care Utilization:

In addition to patient reported emergency department and hospital admission data, we will use the local data-warehouse to capture all of the data needed to determine utilization. Furthermore, we will also use the data from the Indiana Network for Patient Care (INPC) to complement any data use outside of our health system. INPC is the primary health information exchange in the state of Indiana and it provides data for acute care services from all of the health care systems within the state of Indiana. We will determine the number of emergency department visits and the number of re-hospitalizations during follow-up as well as the diagnoses associated with each utilization episode.

Other Data Measures:

Table 7 below shows other data that will be collected.

Data Collection:

Trained staff using Research Electronic Data Capture (REDCap) will collect all data.

Table 7: Other Data Measures

Variables	Method of Collection
Patient demographics(age, gender, race , education, Body Mass Index)	Electronic Medical Records
Comorbidity-level & Chronic co-morbidities	Charlson comorbidity index score [96]
Prior history of depression & anxiety	Electronic Medical records
Medications	Electronic Medical records/Patient/caregiver
Severity of illness	APACHE-II[97] through medical records at ICU admission
Cognitive status prior to admission	LAR interview using the IQCODE [98]
Functional status prior to admission	ADL, IADL (Katz, Lawton scales)[88,89]
Reason for ICU admission and hospitalization	Electronic Medical records
Duration of mechanical ventilation, *noninvasive positive pressure ventilation or high flow nasal canal.* ***delirium, length of ICU and hospital stay***	Electronic Medical records, CAM-ICU 7

IQCODE: *Informant Questionnaire on Cognitive Decline in Elderly;* ***APACHE:*** *Acute Physiology and Chronic Health Evaluation;* ***ADL:*** *Activities of daily living;* ***IADL:*** *Instrumental activities of daily living.* ***LAR***: *Legally Authorized representative*

9. Privacy/Confidentiality Issues

Loss of confidentiality is a risk in this type of data collection. Our data management and quality assurance techniques have proven effective in past trials in maintaining confidentiality and all study personnel have completed training in Human Subjects Research and HIPAA standards.

10. Follow-up and Record Retention

This is a five year study and study documents will be destroyed seven years after the end date of the study.

11. References:

1. Angus, D.C., Kelly, M.A., Schmitz, R.J., White, A., Popovich, J., Committee on Manpower for Pulmonary and Critical Care Societies. "Current and Projected Workforce. Requirements for Care of the Critically Ill and Patients with Pulmonary Disease: Can We Meet the Requirements of an Aging Population?" *JAMA: Journal of the American Medical Association* 284, no. 21 (2000):2762-2770.
2. Joint Commission Resources. Improving Care in the ICU, 1st Edition. Oakbrook Terrace, Illinois: *Joint Commission Resources*, 2004.
3. Society of Critical Care Medicine. Critical Care Units: A Descriptive Analysis, 1st Edition. Des Plaines, Illinois: *Society of Critical Care Medicine*, 2005.
4. Needham DM, Bronskill SE, Calinawan JR, et al. Projected incidence of mechanical ventilation in Ontario to 2026: Preparing for the aging baby boomers. *Crit Care Med 2005*; 33: 574-579.
5. Heathcare Cost and Utilization Project (HCUP). [Accessed December, 6, 2011] Overview of the Nationwide Inpatient Sample. http://www.hcup-us.ahrq.gov/nisoverviewjsp.
6. Stefan MS, Shiesh MS, Pekow PS et al. Epidemiology and Outcomes of Acute Respiratory Failure in United States 2001-2009. A National Survey. *JHM* 2013 (8) 76-82.
7. Herridge MS, Cheung AM, Tansey CM et al. One-year outcomes in survivors of the acute respiratory distress syndrome. *New England Journal of Medicine*. 2003; 348:683-93.
8. Iwashyna TJ, Ely EW, Smith DM, Langa KM. Long-term cognitive impairment and functional disability among survivors of severe sepsis. *JAMA*. 2010; 304:1787-1794.

9. Hough C, Steinberg K, Taylor Thompson B, Rubenfeld G, Hudson L. Intensive care unit-acquired neuromyopathy and corticosteroids in survivors of persistent ARDS. *Intensive Care Medicine.* 2009 Jan 1; 35(1):63-68.
10. McCusker J, Cole M, Dendukuri N, *et al.* Delirium in older medical patients and subsequent cognitive and functional status: A prospective study. *Can Med Assoc J.* 2001; **165**:575–583.
11. Witlox J, Eurelings LS, de Jonghe JF, Kalisvaart KJ, Eikelenboom P, van Gool WA. Delirium in elderly patients and the risk of postdischarge mortality, institutionalization and dementia: a meta- analysis. *JAMA*. 2010 Jul 28;304(4):443-5.1
12. Pandharipande PP, Girard TD, Jackson JC et al. Long Term Cognitive Impairment after Critical Illness. *NEJM* 2013 369(14):1306-16
13. Jackson JC, Girard TD, Gordon SM, et al. Long-term cognitive and psychological outcomes in the awakening and breathing controlled trial. *Am J Respir Crit Care Med*. 2010; 182(2):183-91.
14. Ringdal M, Plos K, Lundberg D, Johansson L, Bergbom I. Outcome after injury: memories, health related quality of life, anxiety, and symptoms of depression after intensive care. *Journal of Trauma*. 2009 Apr 1; 66(4):1226-1233.
15. Adhikari N, McAndrews M, Tansey C, et al. Self-reported symptoms of depression and memory dysfunction in survivors of ARDS. *CHEST*. 2009 Mar 1; 135(3):678-687.
16. Davydow DS, Gifford JM, Desai SV, Bienvenu OJ, Needham DM. Depression in general intensive care unit survivors: a systematic review. *Intensive Care Medicine.* 2009; 35(5):796-809.
17. Davydow DS, Desai SV, Needham DM, Bienvenu OJ. Psychiatric morbidity in survivors of the acute respiratory distress syndrome: a systematic review. *Psychosomatic Medicine*. 2008; 70(4):512-9 (b).
18. Girard TD, Shintani AK, Jackson JC, et al. Risk factors for post-traumatic stress disorder symptoms following critical illness requiring mechanical ventilation: a prospective cohort study. *Critical Care.* 2007; 11:R28. (doi:10.1186/cc5708.
19. Davydow DS, Gifford JM, Desai SV, Needham DM, Bienvenu OJ. Posttraumatic stress disorder in general intensive care unit survivors: a systematic review. *General Hospital Psychiatry*. 2008; 30(5):421-34 (a).
20. McCusker J, Cole M, Abrahamowicz M. *et al.* Delirium predicts 12-month mortality. *Arch Intern Med.* 2002; **162:457**–463.
21. Cuthbertson BH, Roughton S, Jenkinson D, MacLennan G, Vale L. Quality of life in the five years after intensive care: a cohort study. *Crit Care*. 2010; 14(1):R6.
22. Milbrandt EB. Eldadah B. Nayfield S. Hadley E. Angus DC. Toward an integrated research agenda for critical illness in aging. *American Journal of Respiratory & Critical Care Medicine*. 2010;182(8):995-1003.
23. Tan T, Brett SJ, Stokes T. Guideline Development Group. Rehabilitation after critical illness: summary of NICE guidance. *BMJ*. 2009 Mar 25; 338:b822. doi: 10.1136/bmj.b822.
24. Gibson V. Update on NICE guidance CG83 rehabilitation after critical illness. *Nurs Crit Care*. 2010 Jul-Aug; 15(4):222-3.
25. Treacher D, Gibson V, Griffiths J, Kishen R, Morris C, Parker R. 2010 Annual Evidence Update on Critical Illness Rehabilitation. *NHS Evidence*. 2010 March 15. (http://hdl.handle.net/10145/123127).
26. Jones C, Griffiths RD. Identifying post intensive care patients who may need physical rehabilitation. *Clinical Intensive Care*. 2000; 11:35-8.

27. Unroe M, Kahn JM, Carson SS et al. One year trajectories of care and resource utilization for recipients of prolonged mechanical ventilation: a cohort study. *Ann Intern Med*. 2010 Aug 3; 153(3): 167–175.
28. Needham DM, et al. Improving long-term outcomes after discharge from intensive care unit: report from a stakeholders' conference. *Crit Care Med* 2012; 40(2):502-9.
29. Medicine, Institute of. Crossing the Quality Chasm. Washington, DC: Institute of Medicine Press; 2001. Institute of Medicine Press.
30. McDaniel RR, Jr., Jordan ME, Fleeman BF. Surprise, Surprise, Surprise! A complexity science view of the unexpected. *Health Care Manage Rev*. 2003;28(3):266-78.
31. Stroebel CK, McDaniel RR, Jr., Crabtree BF, Miller WL, Nutting PA, Stange KC. How complexity science can inform a reflective process for improvement in primary care practices. *Jt Comm J Qual Patient Saf*. 2005; 31(8):438-46.
32. Committee on the Future Health Care Workforce for Older Americans, Institute of Medicine. Retooling for an Aging America: Building the Health Care Workforce. Washington, DC: Institute of Medicine Press; 2008
33. Callahan CM, Boustani MA, Weiner M et al. Implementing dementia care models in primary care settings: The Aging Brain Care Medical Home. Aging and Mental Health 2011; 15 (1): 5-12.
34. Boustani MA, et al. Implementing innovative models of dementia care: The Healthy Aging Brain Center. *Aging Ment Health* 2011; 15(1):13-22.
35. Counsell SR, Callahan CM, Clark DO, et al. Geriatric care management for low-income seniors: A randomized controlled trial. *JAMA.* 2007; 298(22):2623–2633.
36. Khan BA, Lasiter SA, Boustani ML. Critical Care Recovery Center: Making the Case for an Innovative Collaborative Care Model for ICU Survivors. *American Journal of Nursing*. 2015: March 2015. 115 (3); 24-31.
37. Monahan PO, Alder CA, Khan BA, Stump T, Boustani MA. The Healthy Aging Brain Care (HABC) Monitor: validation of the Patient Self-Report Version of the clinical tool designed to measure and monitor cognitive, functional, and psychological health. *Clinical interventions in aging.* 2014 Dec; 9:2123.
38. Monahan PO, Boustani MA, Alder C et al. Practical clinical tool to monitor dementia symptoms: the HABC-Monitor. *Clin Interv* Aging. 2012; 7:143-57.
39. Frame A, LaMantia M, Reddy Bynagari BA, et al. Development and Implementation of an Electronic Decision Support to Manage the Health of a High-Risk Population: The enhanced Electronic Medical Record Aging Brain Care Software (eMR-ABC). *eGEM 2013;*1: Article 8
40. Ware JE, Sherbourne CD> The MOS 36 item Short-Form Health Survey. *Med Care*. 1992; 30(6): 473-481.
41. Ware JE, Kosinski M, Keller SD. SF-36 Physical and Mental Health Summary Scales: A User's Manual. Boston MA: The Health Institute: New England Medical Center; 1994.
42. Ware JE, Bayliss MS, Rogers WH et al. Differences in 4–year health outcomes for elderly and poor, chronically ill patients treated in HMO and fee-for-service systems; results from the medical outcomes study. *JAMA*, 1996; 276(13): 1039-1047
43. Randolph C, Tierney MC, Mohr E, et al. The Repeatable Battery for the Assessment of Neuropsychological Status (RBANS): Preliminary Clinical Validity. *J Clin Exp Neuropsyc.* 1998;Vol. 20, No. 3:310-319.
44. Guralnik JM, Ferrucci L, Pieper CF, et al. Lower extremity function and subsequent disability:

Consistency across studies, predictive models, and value of gait speed alone compared with the short physical performance battery. *The Journals of Gerontology. Series A, Biological Sciences and Medical Sciences.* Apr 2000; 55(4):M221-231
45. Kroenke K, Spitzer RL, Williams JB. The PHQ-9: validity of a brief depression severity measure. *Journal of general internal medicine.* Sep 2001; 16(9):606-613, PMCID: PMC1495268.
46. Lowe B, Unutzer J, Callahan CM, Perkins AJ, Kroenke K. Monitoring depression treatment outcomes with the patient health questionnaire-9. *Medical care.* Dec 2004; 42(12):1194-1201.
47. Kroenke K, Spitzer RL, Williams JB, Monahan PO, Lowe B. Anxiety disorders in primary care: prevalence, impairment, comorbidity, and detection. *Annals of internal medicine.* Mar 6 2007; 146(5):317-325.
48. Spitzer RL, Kroenke K, Williams JB, Lowe B. A brief measure for assessing generalized anxiety disorder: the GAD-7. *Archives of internal medicine.* May 22 2006; 166(10):1092-1097.
49. Esperson K, Antonsen K, Jensen H. Capacity problems in Danish Intensive Care Units. 2007. *Ugeskrift for Laeger*;169:710-712
50. Iwashyna TJ 2010. Survivorship will be the defining challenge of critical care in the 21st century. *Annals of Internal Medicine* 153:204-205
51. Desai SV, Law TJ, Needham DM et al. Long-term complications of critical care. *Crit Care Med* 2011; 39: 371-379.
52. Steven RD, Dowdy DW, Michaels RK et al. Neuromuscular dysfunction acquired in critical illness: a systematic review. *Intensive Care Med* 2007; 33: 1876-1891
53. Girard TD, Jackson JC, Pandharipande PP, et al. Delirium as a predictor of long-term cognitive impairment in survivors of critical illness. *Crit Care Med* 2010; 38: 1513-1520
54. Dowdy DW, Eid MP, Dennison CR et al. Quality of Life after Acute Respiratory Distress Syndrome: A meta-analysis. *Intensive Care Med* (2006) 32:1115–1124
55. Herridge MS, Cheung AM, Tansey CM et al. One-year outcomes in survivors of the acute respiratory distress syndrome. *New England Journal of Medicine*. 2003;348:683-93.
56. Ringdal M, Plos K, Lundberg D, Johansson L, Bergbom I. Outcome after injury: memories, health related quality of life, anxiety, and symptoms of depression after intensive care. *Journal of Trauma*. 2009 Apr 1; 66(4):1226-1233
57. Cuthbertson BH, Roughton S, Jenkinson D, MacLennan G, Vale L. Quality of life in the five years after intensive care: a cohort study. *Crit Care*. 2010;14(1):R6
58. Milbrandt EB. Eldadah B. Nayfield S. Hadley E. Angus DC. Toward an integrated research agenda for critical illness in aging. *American Journal of Respiratory & Critical Care Medicine*. 2010;182(8):995-1003.
59. Gibson V. Update on NICE guidance CG83 rehabilitation after critical illness. *Nurs Crit Care*. 2010 Jul-Aug;15(4):222-3.
60. Jones C, Griffiths RD. Identifying post intensive care patients who may need physical rehabilitation. *Clinical Intensive Care*. 2000;11:35-8.
61. Volk B, Grassi F. Treatment of the post-ICU patient in an outpatient setting. *Am Fam Physician* 2009;79:459–464
62. Jones C, Skirrow P, Griffiths RD, et al: Rehabilitation after critical illness: A randomized, controlled trial. *Crit Care Med.* 2003; 31:2456–2461.

63. Jackson J, Ely EW, Morey MC, et al. Cognitive and Physical Rehabilitation of intensive care unit survivors: Results of the RETURN randomized controlled pilot investigation. *Crit Care Med* 2012; 40(4):1088-97
64. Elliott D, McKinley S, Alison J, et al: Health related quality of life and physical recovery after a critical illness: A multi-centre randomized controlled trial of a home-based physical rehabilitation program. *Crit Care* 2011;15:R142
65. Cuthbertson BH, Rattray J, Campbell MK, et al: The PRaCTICaL study of nurse led, intensive care followup programmes for improving long term outcomes from critical illness: A pragmatic randomised controlled trial. *BMJ* 2009; 339:b3723
66. Walsh TS, Salisbury LG, Merriweather JL et al. Increased hospital-based physical rehabilitiaion and information provision after intensive care unit discharge. The RECOVER Randomized Clinical Trial. *JAMA Internal Medicine* 2015. Apr 13. doi: 10.1001/jamainternmed.2015.0822. [Epub ahead of print]
67. Unutzer J, Katon W, Callahan CM, et al. Collaborative Care Management of Late-Life Depression in the Primary Care Setting. A Randomized Controlled Trial. *JAMA* 2002; 288:2836-2845.
68. Callahan CM, Boustani MA, Unverzagt FW, et al. Effectiveness of collaborative care for older adults with Alzheimer disease in primary care: a randomized controlled trial. *JAMA.* May 10 2006;295(18):2148-2157.
69. Prentice JC, Pizer SD. Delayed Access to Health Care and Mortality. *Health Services Research.* 2007; 42(20): 644–662.
70. Berwick DM, Nolan TW, Whittington J. The Triple AIM; Care, Health and Cost. *Health Aff* **2008; 27 (3) 759-769.**
71. Sprenkle MD, Niewoehner DE, Nelson DB et al. The Veterans Short Form 36 Questionnaire is predictive of mortality and health-care utilization in a population of veterans with a self-reported diagnosis of asthma or COPD. *CHEST* 2004; 126:81-89.
72. Lamantia MA, Alder CA, Callahan CM et al. The Aging Brain Care Medical Home: Preliminary Data. *JAGS* 2015;63(6):1209-1213
73. Campbell N, Khan B, Farber M, et al. Improving the care of delirium in the ICU: Design of a pragmatic study. *Trials.* 2011 Jun 6; 12:139.
74. Khan BA, Guzman O, Campbell NL et al. Comparison and agreement between two sedation scales; Richmond Agitation Sedation Scale and Sedation Agitation Scale in evaluating patients' eligibility for Delirium assessment in the Intensive Care Unit. *CHEST.* 2012 Jul; 142(1):48-54.
75. Khan BA, Farber MO, Campbell NL et al. S-100 calcium binding protein as a biomarker for delirium duration in the intensive care unit. An exploratory analysis. *IJGM.* 2013:2 (6) 855-861.
76. Khan BA, Calvo-Ayala E, Campbell NL, et al. Clinical Decision Support System and Incident Delirium among Cognitively Impaired Hospitalized Older Adults Transferred to the Intensive Care Unit. *Am J Crit Care.* 2013 May; 22(3):257-62.
77. Khan BA, Fadel WF, Tricker JL, et al. Effectiveness of Implementing a Wake Up and Breathe Program on Sedation and Delirium in the ICU. *Critical care medicine.* 2014 Dec;42(12):e791-e795.
78. Khan BA, Perkins A, Hi SL, et al. Relationship between African-American Race and Delirium in the Intensive Care Unit. *CCMED* 2015. In press.

79. Wang S, Lasiter S, Zarzaur B, Campbell T, Boustani M, Khan B. Critical Care Recovery Center (CCRC): Can a Geriatric Model of Care Guide Recovery of ICU Survivors? *Best Practices in Mental Health*. 2016; 12(2) (In press)
80. Sidani S, Braden CJ. Design, Evaluation, and Translation of Nursing Interventions, First Edition. 2011 John Wiley and Sons, Inc
81. Roussos C, Koutsoukou A. Respiratory Failure. Eur Respir J 2003:22 (S47): 3s-14s
82. Conigrave KM, Hall WD, Saunders JB. The AUDIT questionnaire: choosing a cut-off score. *Addiction* 1995. 90, 1349-1356
83. Skinner H. The Drug Abuse Screening Test. *Addict Behav* 1982. 7: 363-371
84. Oles SK, Stephens N, Bubp CA, Shepard P, Campbell T, Boustani MA, Khan BA. Development and Implementation of a Novel Cognitive Rehabilitation Protocol for Intensive Care Unit Survivors. *Am J Respir Crit Care Med* 191;2015:A4504
85. Clark DO, Chrysler L, Perkins A, Keith NR, Willis DR, Abernathy G, Smith F. Screening, referral, and participation in a weight management program implemented in five CHCs. *Journal of Health Care for the Poor and Underserved*. 2010; 21(2), 617-628. PMID: 20453361
86. Folstein MF, Folstein SE, McHugh PR. Mini-mental state". A practical method for grading the cognitive state of patients for the clinician". *Journal of Psychiatric Research* 1975;12 (3): 189–98.
87. Podsiadlo D, Richardson S. The Timed Up and Go: A Test of Basic Functional Mobility for Frail Elderly Persons. *JAGS*. 1991;39:142–148.
88. Katz S, Ford AB, Moskowitz RW, Jackson BA, Jaffe MW. Studies of illness in the aged. The index of Adl: A standardized measure of biological and psychosocial function. *J Am Med Assoc.* 1963;185:914-919.
89. Lawton MP, Brody EM. Assessment of older people: self-maintaining and instrumental activities of daily living. *Gerontologist.* 1969; 9:179-186.
90. Zigmond AS, Snaith RP. The hospital anxiety and depression scale. *Acta Psychiatr Scan*. 1983:67(6):361-370.
91. Minns Lowe CJ, Wilson MS, Sackley CM, Barker KL. Blind outcome assessment: the development and use of procedures to maintain and describe blinding in a pragmatic physiotherapy rehabilitation trial. *Clin Rehabili*. 2011. 25(3):264-74
92. Boutron IS, Guittet L, Estellat C et al. Reporting methods of blinding in randomized trials assessing nonpharmacological treatments. *PLoS Med* 2007. 4(2): e61
93. Pagoto S, McDermott MM, Reed G et al. Can attention control conditions have detrimental effects in behavioral medicine randomized trials? *Psychosomatic Medicine*. 2013. 75(2): 137-143
94. Freedland KE. Demanding Attention: Reconsidering the Role of Attention Control groups in Behavioral Intervention research. *Psychosomatic Medicine*. 2013 75:100-102
95. Pahor M, Guralnik JM, Ambrosius WT et al. Effect of structured physical activity on prevention of major mobility disability in older adults. The LIFE study randomized clinical trial. *JAMA*. 2014. 311(23): 2387-2396
96. Charlson ME, Sax FL, MacKenzie CR, Fields SD, Braham RL, et al. Resuscitation: how do we decide? A prospective study of physicians' preferences and the clinical course of hospitalized patients. *J Am Med Assoc.* 1986; 255:1316-1322.
97. Knaus WA, Draper EA, Wagner DP, Zimmerman JE. APACHE II: a severity of disease classification system. *Crit Care Med*. 1985; 13:818-829.

98. Jorm AF, Jacomb PA. The Informant Questionnaire on Cognitive Decline in the Elderly (IQCODE): socio-demographic correlates, reliability, validity and some norms. *Psychol Med* 1989; 19:1015-1022.
99. Little, R.J.A. and Rubin, D.B. (2002). *Statistical Analysis with Missing Data*, 2nd edition, New York: John Wiley.
100. T.M. Therneau, P.M. Grambsch Modeling Survival Data: Extending the Cox Model Springer-Verlag, New York, NY (2000), pp. 169–229.
101. Sukantarat K, Greer S, Brett S, et al: Physical and psychological sequelae of critical illness. *Br J Health Psychol* 2007; 12:65–74.
102. Kernan WN, Viscoli CM, Makuch RW, Brass LM, Horwitz RI. Stratified Randomization for Clinical Trials. *Journal of Clinical Epidemiology.* 1// 1999;52(1):19-26
