# Supplementary material for: Mobile Critical Care Recovery Program for Survivors of Acute Respiratory Failure: A Randomized Clinical Trial
Source: JAMA Netw Open. 2024 Jan 30;7(1):e2353158. doi: 10.1001/jamanetworkopen.2023.53158 (PMC10828910; doi:10.1001/jamanetworkopen.2023.53158)
Supplement: Supplement 2. — eMethods. Methods, Intervention, Effect of COVID-19 on Study Operations, m-CCRP Care Protocols and Handouts, and Attention Control ICU Survivor Guide eBox. m-CCRP Care Coordinator Services eTable 1. m-CCRP Intervention Effects on SF-36 Subscales eTable 2. m-CCRP Intervention Effects on Repeatable Battery for the Assessment of Neuropsychological Status (RBANS) Subscales eTable 3A. Time to Emergency Department Visit, Re-Hospitalization, and Death Between m-CCRP Intervention and Control groups. eTable 3B. Time to Inpatient Hospitalization by Discharge Location Between m-CCRP Intervention and Control groups. eTable 3C. Comparison of Emergency Department Visits and Re-hospitalizations Between Patients Discharged to Home Versus Facility in Both Control and m-CCRP Groups eTable 4A. Emergency Department Diagnoses and Reasons at the Encounter Level eTable 4B. Hospital Readmission Diagnoses and Reasons at the Encounter Level eTable 5. Initiation of m-CCRP Protocols by Discharge Status eTable 6. Days From Discharge to Implementation of m-CCRP Protocols eTable 7. Comparison of Outpatient Orders and Medication Orders Between m-CCRP and Control eTable 8. Comparison of Outpatient Care Related to Initiated Protocols Between m-CCRP and Control eTable 9. Comparison of Study Outcomes Between Study Groups in Participants ≥65 Years of Age eTable 10. Comparison of Study Outcomes Between Study Groups in Participants With Baseline PHQ-9 ≥10 eTable 11. Comparison of Study Outcomes by Number of m-CCRP Care Coordinator-Patient Contacts eTable 12. Comparison of Study Outcomes When the m-CCRP Group is Divided into Those With >25% Reduction in Healthy Aging Brain Care Monitor (HABC-M Self Report) Symptom Scores and Those With No Reduction. eTable 13. Effect of m-CCRP Intervention on Patient Outcomes Pre and During COVID-19 Pandemic eTable 14. Effect of m-CCRP Intervention on Patient Outcomes by Discharge Status eTable 15. Comparison of Patient Baseline Characteristics by Completion Status at 12 [file jamanetwopen-e2353158-s002.pdf]

Supplementary Online Content

Khan BA, Perkins AJ, Khan S, et al. Mobile Critical Care Recovery Program for survivors of acute respiratory failure: a randomized clinical trial. *JAMA Netw Open*. 2024;7(1):e2353158. doi:10.1001/jamanetworkopen.2023.53158

eMethods. Methods, Intervention, Effect of COVID-19 on Study Operations, m-CCRP Care Protocols and Handouts, and Attention Control ICU Survivor Guide

eReferences

eBox. m-CCRP Care Coordinator Services

eTable 1. m-CCRP Intervention Effects on SF-36 Subscales

eTable 2. m-CCRP Intervention Effects on Repeatable Battery for the Assessment of Neuropsychological Status (RBANS) Subscales

eTable 3A. Time to Emergency Department Visit, Re-Hospitalization, and Death Between m-CCRP Intervention and Control groups.

eTable 3B. Time to Inpatient Hospitalization by Discharge Location Between m-CCRP Intervention and Control groups.

eTable 3C. Comparison of Emergency Department Visits and Re-hospitalizations Between Patients Discharged to Home Versus Facility in Both Control and m-CCRP Groups

eTable 4A. Emergency Department Diagnoses and Reasons at the Encounter Level

eTable 4B. Hospital Readmission Diagnoses and Reasons at the Encounter Level

eTable 5. Initiation of m-CCRP Protocols by Discharge Status

eTable 6. Days From Discharge to Implementation of m-CCRP Protocols

eTable 7. Comparison of Outpatient Orders and Medication Orders Between m-CCRP and Control

eTable 8. Comparison of Outpatient Care Rlated to Initiated Protocols Between m-CCRP and Control

eTable 9. Comparison of Study Outcomes Between Study Groups in Participants ≥65 Years of Age

eTable 10. Comparison of Study Outcomes Between Study Groups in Participants With Baseline PHQ-9 ≥10.

eTable 11. Comparison of Study Outcomes by Number of m-CCRP Care Coordinator-Patient Contacts

eTable 12. Comparison of Study Outcomes When the m-CCRP Group is Divided into Those With >25% Reduction in Healthy Aging Brain Care Monitor (HABC-M Self Report) Symptom Scores and Those With No Reduction.

eTable 13. Effect of m-CCRP Intervention on Patient Outcomes Pre and During COVID-19 Pandemic

eTable 14. Effect of m-CCRP Intervention on Patient Outcomes by Discharge Status

eTable 15. Comparison of Patient Baseline Characteristics by Completion Status at 12 Months

eTable 16. Comparison of Patient Outcomes by Study Groups Over Time Including Adjustment for ICU Length of Stay and Mechanical Ventilation Duration

eTable 17. Comparison of Patient Outcomes by Study Groups Over Time for m-CCRP Patients With at Least 9 Contacts (Per Protocol Analysis)

eFigure 1. m-CCRP Intervention Results Based on Selected Sub-Group Analyses

eFigure 2. Changes in Intervention Measures in the m-CCRP Group

This supplementary material has been provided by the authors to give readers additional information about their work.

© 2023 Khan BA et al. *JAMA Network Open*.

eMethods. Methods, Intervention, Effect of COVID-19 on Study Operations, m-CCRP Care Protocols and Handouts, and Attention Control ICU Survivor Guide

Study Setting: We enrolled patients from March 2017 to April 2022 admitted to the ICU services of four Indiana hospitals (one community, one county, and two academic), affiliated with Indiana University (IU) School of Medicine. Eskenazi Health is a 310-bed, urban public hospital with 44 ICU beds. IU Health Methodist Hospital is an 802-bed tertiary care hospital with 100 ICU beds. IU Health University Hospital is a 257-bed tertiary care hospital with 18-bed Medical and 18-bed Surgical ICU. IU Health West Hospital is a 177-bed community-based hospital with 14 ICU beds.

Other Data Collection: Demographics, severity of illness measured by Acute Physiology and Chronic Health Evaluation Score (APACHE-II),[1] chronic co-morbidities (Charlson comorbidity index),[2] medications, cognition prior to admission (IQCODE),[3] activities and instrumental activities of daily living,[4,5] duration of mechanical ventilation, and length of hospital and ICU stay were also collected and stored in a Research Electronic Data Capture (REDCap) database.

m-CCRP Intervention:

Care Coordinator*:* The m-CCRP intervention was led by a nurse care coordinator with training in health coaching, case management, community organizing, and nursing care. The care coordinator received additional training in a) recognizing and managing PICS symptoms; monitoring the biopsychosocial needs and outcomes of ICU survivors; c) monitoring negative effects of medications; d) delivering recovery protocols; e) enhancing the self-management capacity of patients and their caregivers; f) communicating and coordinating care with primary and specialty healthcare providers; g) and coordinating community resources. The care coordinator conducted patient visits at both home and healthcare facilities, collaborated with the m-CCRP interdisciplinary team, maintained communication with the patient's primary care provider and specialists, implemented the individualized care plans, and monitored the effectiveness of care plans (Supplementary Box 1).

Interdisciplinary team*:* The m-CCRP team consisted of two critical care physicians, a geriatrician, an ICU nurse, and a neuropsychologist. The team met weekly with the care coordinator to discuss individual care plans.

Care Protocols: The care coordinator utilized non-pharmacological protocols to optimize cognition, physical function, personal care, mobility, sleep disturbances, pain, depression, anxiety, agitation or aggression, delusions or hallucinations, stress and physical health, legal and financial needs, and medication adherence.

Intervention: The care coordinator conducted a face-to-face or telephone encounter with the patient within one week of randomization. The coordinator assessed the patient's cognitive, physical, psychological status using the Mini Mental State Examination (MMSE),[6] Timed up and Go test,[7] Activities of daily living (Katz scale),[4] Instrumental activities of daily living (Lawton Scale),[5] Hospital Anxiety and Depression scale (HADS),[8] and the HABC-Monitor.[9-11] Pain was assessed with Pain Screening Tool (PEG),[12] and sleep through PROMIS Sleep Disturbance Short Form 4a.[13] The care coordinator reconciled all prescribed and over the counter medicines and coordinated all scheduled and recommended appointments made at discharge with specialists and therapists.

Creation of an Individualized Care Plan: Using the assessments and the information provided by the patient, the coordinator collaborated with the m-CCRP team to prepare the individualized care plan.

Delivery of the Care Plan: During the second visit within two weeks of the initial assessment, the coordinator reviewed the individualized recovery care plan with both the patient and the caregiver. This process included a) understanding the diagnoses; b) process of monitoring the patient's recovery; c) implementation of the appropriate care recovery protocols; d) distribution and explanation of the corresponding educational recovery handouts; and e) connection to in-home services and community resources.

The 12-month Intervention Period: This period included ongoing interactions between the care coordinator, the patients and the caregivers via home or clinic visit, phone contact, email, fax or mail. The amount of contact was

© 2024 Khan BA et al. *JAMA Network Open*.

scheduled to be every two weeks for the first 6 months and once per month for the last 6 months of the intervention. During these interactions, the coordinator answered any questions generated from previous visits; collected patient and caregiver's feedback; reviewed and reconciled medications and discussed adherence; reviewed specialists and therapists' appointments and adherence to care plans; administered the HABC-M to trigger or modify the use of specific care recovery protocols; and facilitated access to community resources. The m-CCRP team and care coordinator met once per week to discuss patients' progress. If a patient develops an acute illness that requires hospitalization, the team activated the acute care transition phase where the coordinator contacted the hospital team and provided them with relevant information about the patient. Following a hospital discharge, the coordinator conducted a visit within 72 hours to reconcile medications and coordinate post discharge care plan. During the COVID-19 pandemic, all interaction were conducted via phone.

Process Measures: We collected the number of nurse contacts per patient and the number of protocols initiated per patient. We also utilized the Healthy Aging Brain Care Monitor (HABC-M) to monitor the cognitive, physical, and psychological symptoms of ARF survivors.[9-11] The HABC-M monitor has 27 items tapping the previous constructs. While the total HABC-M score is helpful to measure change over time, each question also indicates a specific care area where help or coping strategies might be indicated. The HABC-M served as a longitudinal monitoring tool to guide the personalization of the m-CCRP care protocols to the needs of the patient at any point in time during recovery. The HABC-M also provided dynamic feedback to allow for adaptation and customization of the intervention in real time by the care coordinator.

Statistical Analysis: We used time-to-event models to analyze time from enrollment to emergency department visits, hospital readmissions and death with group as the independent variable while adjusting for site, noninvasive ventilation (stratification variables) as well as age, APACHE II, Charlson Comorbidity Index, and ICU length of stay. Patients who are followed to 12 months without experiencing an event had their event time censored at 12 months; patients who died or lost to follow-up had their time to ED or rehospitalization censored at the time of death or date of last contact. Recurring ED visits or rehospitalization were modeled using the Andersen-Gill model for multiple events using elapsed times with robust variance. The use of the model for recurrent events allows us to detect intervention effect on reducing acute care utilization rates as well as on delaying the time to utilization. Additional analyses such as sub-group analyses, analysis on sub-scales of outcome measures and time to event models for ED, rehospitalization and mortality are included in supplementary material. All analyses were conducted using SAS 9.4 software.

© 2024 Khan BA et al. *JAMA Network Open*.

Effect of COVID-19 on Study Operations:

Operation	COVID-19 Restrictions	Study Response
Outcomes assessments	IU research shutdown of in-person activities and extended COVID-19 policies restricted home visits for Baseline, 3-Month, and 6-Month, and 12-month outcome assessments.	Outcomes assessments transitioned to remote administration in March 2020 and continued through the end of the study. Only a subset of outcome measures was suitable for telephone administration:<br>1. SF-36<br>2. RBANS Subtests:<br>o List Learning<br>o Story Memory<br>o Semantic Fluency<br>o Digit Span<br>o List Recall<br>o List Recognition<br>o Story Recall<br>3. GAD-7<br>4. PHQ-9<br>5. ISI-7<br>6. PTSS-10<br>7. Employment Questionnaire<br>8. Medication Collection<br><br>Measures that could not be collected by telephone administration:<br>9. MOCA<br>10. SPPB<br>11. Step test<br>12. RBANS subtests<br>Figure Copy<br>Line Orientation<br>Picture Naming<br>Coding<br>Figure Recall<br>13. Trail Making Test Parts A & B
Intervention	IU research shutdown of in-person activities and extended COVID-19 policies restricted home visits for Intervention delivery.<br><br>The local health systems restricted home visits for their personnel as well, which included the study care coordinator sub-contracted through the health system.	Intervention was restricted to a telephone-based intervention during the COVID-19 pandemic.

© 2024 Khan BA et al. *JAMA Network Open*.

Mobile Critical Care Recovery Program (m-CCRP) for Acute Respiratory Failure (ARF) Survivors

Study Protocols

1. **Cognition**
2. **Exercise**
3. **Depression**
4. **Anxiety**
5. **Physical Health**
6. **Behavioral Care**
7. **Legal and Financial**
8. **Communication**
9. **Mobility**
10. **Personal Care**
11. **Sleep Disturbance**
12. **Pain**
13. **Stress**
14. **Acute Care Reduction Protocol/DELIRIUM**
15. **Medication Adherence**

© 2024 Khan BA et al. *JAMA Network Open*.

Cognition Protocol

- MMSE will be performed by the care coordinator on the initial assessment
- If MOCA is <27, cognitive therapy exercises will be initiated. Patient will perform these exercises once/day for 3 months then three times/week for the next nine months. (Give cognitive therapy handout) (Brain health handouts from CCRC)
- At each contact HABC Monitor will be completed
- Based on the responses on the HABC Monitor, individualized recommendations will be discussed with caregiver and family
- M-CCRP Protocols targeted by HABC responses will be discussed and appropriate handouts given and reviewed
- Patients/caregivers who have an increased in the HABC Caregiver Monitor score, based on eMR tracking, will be presented at weekly team meetings for problem solving and continuity of care
- The patient's PCP and m-CCRP team will be updated regarding progress and concerns
- At each contact medication reconciliation will be completed
- Recommendations to stop all anticholinergic medications will be made
- During interaction with the patient/caregiver and/or responses on the HADS/HABC Caregiver Monitor, if there is an indication of depression, then the depression protocol will be initiated

Instruct caregiver about COGNITIVE IMPAIRMENT:

1. Consider the following Caregiver Handouts:
   - Brain Exercises
   - Anticholinergic Burden Scale
2. Encourage meaningful activities that the patient with MCI enjoys.

© 2024 Khan BA et al. *JAMA Network Open*.

HOME Exercises Protocol

Perform TUG test on patients. If they score >9 seconds, the exercise protocol will be initiated.

All exercises may be performed while seated or standing – if standing, use a chair or wall for support as needed. Remember to take breaks as needed and drink water during your breaks.

Warm-Up

Upper Body

1. 10 wrist circles inward (right and left hand together or independently)
2. 10 wrist circles outward (right and left hand together or independently)
3. 10 shoulder circles forward (both shoulders)
4. 10 shoulder circles backward (both shoulders)
5. Move chin to look over right shoulder, then center, then left shoulder
6. Drop chin to chest, roll chin to right shoulder, stop, roll chin to left shoulder, stop, return to center, raise head
7. Rotate body to grab the left side of the chair (hold for 10 seconds)
8. Rotate body to grab the right side of the chair (hold for 10 seconds)
9. Grasp hands, turn palms out, push arms forward (hold for 10 seconds) and push arms upward (hold for 10 seconds)
10. Reach left arm across chest, press back the upper part of the left arm with the right hand (hold for 10 seconds)
11. Reach right arm across chest, press the upper part of the right arm with the left hand (hold for 10 seconds)

Return to first exercise and complete exercises 1-11 (two to three times total)

Lower Body

1. 10 ankle circles inward (right foot then left foot or together)
2. 10 ankle circles outward (right foot then left foot or together)
3. Place heels on the floor, point toes toward the ceiling, then point toes forward (repeat 10 times)
4. Place right heel of floor, bend forward at waist, keep head and eyes facing forward (hold for 10 seconds)
5. Place left heel of floor, bend forward at waist, keep head and eyes facing forward (hold for 10 seconds)
6. Raise right knee toward chest (hold lower right leg for ten seconds)
7. Raise left knee toward chest (hold lower left leg for ten seconds)

Return to first exercise and complete exercises 1-7 (two to three times total)

Exercises – perform each exercise 10 times and repeat 2-3 times. If possible, alternate upper body and lower body

Upper Body	***Lower Body and Core***
1. Upright rows	1. Single heel tap (extend one leg, tap the heel to the floor, and return, repeat on opposite leg)
2. Shoulder press	2. Single knee raises (hips abducted)
3. Small arm circles (forward then backward)	3. Single leg kick (extend leg forward, do not tough the heel to the floor, return, repeat on opposite leg
4. Chest flys (bring elbows together)	4. Single knee raises (hips adducted)
5. Arm (biceps) curls	5. Double heel tap (seated only)
6. Small arm circles (forward then backward)	6. Double knee raises (hips abducted – seated only)
7. Arm extensions (overhead fist to front)	7. Double leg kick (seated only)
8. Lateral elbow raises	8. Double knee raises (seated only)
9. Lateral arm raises	9. Chair stands (if possible avoid using hands)
10. Forward arm raises	10. Backward leg extensions (use wall or back of chair for support – standing only)
11. Large arm circles (forward then backward)	11. Lateral leg extensions (use wall or back of chair for support – standing only)
12. Lateral rows	12. Backward leg (hamstring) curls – standing only

© 2024 Khan BA et al. *JAMA Network Open*.

Protocol for Care – EXERCISE

Instruct patient/caregiver about EXERCISE:

1. Consider the following Caregiver Handouts:
   - Help with Exercise (Handout #8)
   - Activities to Encourage (Handout #9)
   - Activities to Avoid (Handout #10)
2. Consider the following Patient Handouts:
   - Help with Exercise (Handout #7)
   - Activities to Encourage (Handout #8)
   - Activities to Avoid (Handout #9)
3. Being active may lead to good sleep and decreased agitation, stress or moodiness for both the patient and the caregiver
4. To increase physical activity:
   - Turn on your favorite music and dance for at least 10 minutes or longer.
   - Try to follow the routine on a free exercise program, like "SIT AND BE FIT" on your local PBS station. Program details can be found at www.sitandbefit.org
   - Go for a walk in your neighborhood. Again, try to walk for at least 10 minutes.
   - Have the patient ride a stationary exercise bike for at least 10 minutes at a time. Be sure to start slowly, and then gradually build up speed.
   - Attend an exercise class.
   - Watch the exercise DVD and try to follow along.

© 2024 Khan BA et al. *JAMA Network Open*.

Depression Domain Protocol

Initial contact

- Patients will be screened for depressive symptoms using the Hospital Anxiety and Depression Scale.

Step 1

- If the HADS is positive for depression (HADS score >7), the m-CCRP care coordinator will discuss the diagnosis with the patient and/or caregiver.
- If thyroid function tests (TFTs) have not been evaluated within the past six months the care coordinator will discuss with PCP to draw TFTs and to start treatment (if hypothyroidism present). If treatment started or dosage increased for hypothyroidism the patient will be reevaluated in four to six weeks for repeat TFTs.
- Once TFTs are within normal range, treatment options of starting an antidepressant and Problem Solving Therapy (PST) will be discussed with the patient. If a patient has failed or refuses PST, then Cognitive Behavioral Therapy (CBT) will be offered.
- If the patient elects to start an antidepressant, the care coordinator will work with PCP/m-CCRP team to prescribe an SSRI, after reviewing recent electrolytes and renal function. If labs have not been evaluated in the last six months, the care coordinator will arrange draw for baseline.
- Follow up will occur, by phone or face to face, to monitor symptoms with symptom monitoring through the HABC Monitor.
- Problem Solving Therapy/Cognitive Behavioral therapy. PST/CBT will be conducted for 6-8 weekly sessions.
- Patient may be referred for Psychiatric consultation, as recommended by m-CCRP Team.
- Reassessment results will be reviewed in the weekly team meeting.

Step 2

- If no response or partial response to depression treatment in 6 weeks, consider increasing the antidepressant dose, switch to another antidepressant, augment with a second agent, or referral to psychiatry.
- At this time the patient may need to be referred for psychiatric consultation. Patients with a history of inpatient psychiatric hospitalization within the past 10 years, history of suicide attempt or self-harm requiring emergent medical or psychiatric intervention, history of severe mental illness, treatment in a day program, or electroconvulsive therapy will be referred for psychiatric consultation.
- Biweekly visits will continue for 6 months, at which time a reassessment will occur with HADS.
- If patient is in remission, depression clinical specialist will contact patient monthly.

Step 3/Maintenance

- If symptoms persist, psychiatric referral will be recommended.
- The depression clinical specialist will continue monthly follow up.
- During interaction with the caregiver and/or their responses on the HABC Caregiver Monitor, if there is an indication of depression, then a PHQ 9 will be completed with the caregiver.
- If the caregiver PHQ 9 is positive for depression, then depression information will be provided, as well as a discussion of resources and referral to the caregivers PCP.

Instruct caregiver in DEPRESSION

1. Consider the following Caregiver Handouts:
    - Understanding nonverbal Messages (Handout #6)
    - Help with exercise (Handout #8)
    - Activities to Encourage (Handout #9)
    - Activities to Avoid (Handout #10)
    - Help with Sleeping (Handout #22)
    - Help with Nonverbal Noises (Handout #25)
2. Consider the following Patient Handouts:

© 2024 Khan BA et al. *JAMA Network Open*.

- Help with exercise (Handout #7)
- Activities to Encourage (Handout #8)
- Activities to Avoid (Handout #9)
- Help with Sleeping (Handout #19)

3. Provide a bright and cheerful environment.
4. Activities
   - Increase and encourage activities that the patient can enjoy.
   - Identify activities that the patient enjoyed in the past.
   - Modify activities to reflect patient's currently level of function.
   - Keep activities simple, i.e., sing a song.
   - Play favorite music or read a book to the patient.
   - Use one or two-word instructions at a time to explain activity.
   - Observe the patient to confirm enjoyment of activities in which they are encouraged to participate.
   - Change activity when patient becomes bored or anxious.
   - Activities should be simple and last no more than 30 minutes at a time.
5. Encourage social interaction with others.
   - Plan pleasant activities with people the patient enjoys, especially if patient complains of being lonely.
   - Encourage the patient to talk about pleasant things, both past and present. Thinking about happy events and helping them remember good things can cheer them. It can also make them feel more depressed about the past, so be sensitive.
   - If a patient cannot carry on a conversation, you should remember for them.
   - Too many visitors can be overwhelming
     - Encourage friends to visit one at a time.
     - Encourage friends to talk to patient and maintain eye contact.
     - Have young children visit, but keep visits short if possible.
6. Redirect the patient's attention and provide different activity when the patient expresses feelings of worthlessness, homelessness, or being a burden to others.
7. Touch and hug the patient when he/she is crying and tearful. Reassure the patient that they are not alone.
8. Individualize all of the above approaches to the patient's needs and situation.
9. Consider antidepressant medication if depression persists or worsens.

Instruct caregiver about SUICIDE

10. Threats or statements about suicide should always be taken seriously. If a patient endorses current thoughts of suicide or self-harm or answers 1, 2, or 3 to question #9 to PHQ-9, complete the Columbia Suicide Severity Scale. Available at http://www.integration.samhsa.gov/clinical-practice/Columbia_Suicide_Severity_Rating_Scale.pdf
11. Notify the nurse practitioner/physician immediately. Remain in the home setting until you have discussed the case with the nurse practitioner/physician. If a patient is actively trying to hurt himself/herself or answers yes to 3, 4, or 5 on the suicide severity rating scale, call 911 and also inform the nurse practitioner/physician so the patient can be evaluated in the nearest ER. If a patient answers yes to only 6, then discuss with the nurse practitioner/physician whether an ER visit is warranted.
12. Remove or lock up guns, knives, scissors, and tools form environment.
13. Medications should be locked up and administered by the caregiver.

© 2024 Khan BA et al. *JAMA Network Open*.

Anxiety Protocol

Initial contact

- Patients will be screened for anxiety symptoms using the Hospital Anxiety and Depression Scale.

Step 1

- If the HADS is positive for anxiety (HADS score >7), the m-CCRP care coordinator will discuss the diagnosis with the patient and/or caregiver.
- Treatment options of starting an anxiolytics/antidepressant and Problem Solving Therapy (PST)/Cognitive Behavioral Therapy (CBT) will be discussed with the patient. PST will be offered it patient had comorbid depression. If patient has only anxiety symptoms, CBT will be offered. If patient refuses or has failed one psychotherapy modality, then the other will be offered.
- If the patient elects to start an antidepressant, the care coordinator will work with PCP/m-CCRP team to prescribe an SSRI, after reviewing recent electrolytes and renal function. If labs have not been evaluated in the last six months, the care coordinator will arrange draw for baseline.
- Follow up will occur, by phone or face to face, to monitor symptoms with symptom monitoring through the HABC Monitor.
- Problem Solving Therapy/Cognitive Behavioral therapy. PST/CBT will be conducted for 6-8 weekly sessions with the possibility of extension if patient is responding.
- Patient may be referred for Psychiatric consultation, as recommended by m-CCRP Team.
- Reassessment results will be reviewed in the weekly team meeting.

Step 2

- If no response or partial response to treatment in 3 months, consider increasing the medication dose, switch to another medicine, augment with a second agent.
- At this time the patient may need to be referred for psychiatric consultation.
- Biweekly visits will continue for 6 months, at which time a reassessment will occur with HADS.
- If patient is in remission, clinical specialist will contact patient monthly.

Step 3/Maintenance

- If symptoms persist, psychiatric referral will be recommended.
- The clinical specialist will continue monthly follow up.

Instruct caregiver in ANXIETY

1. Consider the following Caregiver Handouts:
   a. Understanding nonverbal Messages (Handout #6)
   b. Help with exercise (Handout #8)
   c. Activities to Encourage (Handout #9)
   d. Activities to Avoid (Handout #10)
   e. Help with Sleeping (Handout #22)
   f. Help with Nonverbal Noises (Handout #25)

2. Consider the following Patient Handouts:
   - Help with exercise (Handout #7)?
   - Activities to Encourage (Handout #8)
   - Activities to Avoid (Handout #9)
   - Help with Sleeping (Handout #19)
3. Provide a bright and cheerful environment.

© 2024 Khan BA et al. *JAMA Network Open*.

4. Activities
   a. Increase and encourage activities that the patient can enjoy.
   b. Identify activities that the patient enjoyed in the past.
   c. Modify activities to reflect patient's currently level of function.
   d. Keep activities simple, i.e., sing a song.
   e. Play favorite music or read a book to the patient.
   f. Use one or two-word instructions at a time to explain activity.
   g. Observe the patient to confirm enjoyment of activities in which they are encourage to participate.
   h. Change activity when patient becomes bored or anxious.
   i. Activities should be simple and last no more than 30 minutes at a time.
5. Encourage social interaction with others.
   a. Plan pleasant activities with people the patient enjoys, especially if patient complains of being lonely.
   b. Encourage the patient to talk about pleasant things, both past and present. Thinking about happy events and helping them remember good things can cheer them. It can also make them feel more depressed about the past, so be sensitive.
   c. If a patient cannot carry on a conversation, you should remember for them.
   d. Too many visitors can be overwhelming
      i. Encourage friends to visit one at a time.
      ii. Encourage friends to talk to patient and maintain eye contact.
      iii. Have young children visit, but keep visits short if possible.
6. Redirect the patient's attention and provide different activity when the patient expresses feelings of worthlessness, homelessness, or being a burden to others.
7. Touch and hug the patient when he/she is crying and tearful. Reassure the patient that they are not alone.
8. Individualize all of the above approaches to the patient's needs and situation.

© 2024 Khan BA et al. *JAMA Network Open*.

Protocol for Care – PHYSICAL HEALTH

1. Consider the following Caregiver Handouts
   - For the Caregiver (Handout #1)
   - Help with Exercise (Handout #8)
   - Activities to Encourage (Handout #9)
   - Activities to Avoid (Handout #10)

2. Consider the following Patient Handouts
   - For the Patient (Handout #1)
   - Help with Exercise (Handout #7)
   - Activities to Encourage (Handout #8)
   - Activities to Avoid (Handout #9)
3. For the Caregiver remember:
   - You must take care of your physical and emotional health first or you will not be able to help anyone else.
   - Keep your own doctor’s appointments.
   - Take your medications as directed.

© 2024 Khan BA et al. *JAMA Network Open*.

Protocol for Behavioral Care – AGGRESSION/AGITATION/REPITITIVE BEHAVIORS/DELUSIONS/HALLUCINATIONS/PARANOIA/WANDERING

1. **Assess for history of aggression/agitation**
2. **Consider medical intervention**

Instruct caregiver to:

1. Intervene early. By recognizing a problem situation and intervening before it becomes a crisis, caregivers can avoid many instances of agitation and aggression.

Instruct caregiver about COMBATIVE/DESTRUCTIVE BEHAVIOR:

2. Consider the following Caregiver Handouts
    - Suggestions for Communication (Handout #5)
    - Understanding Nonverbal Messages (Handout #6)
    - Nonverbal Messages (Handout #7)
    - Activities to Encourage (Handout #9)
    - Activities to Avoid (Handout #10)
    - Help with Verbal Noises (Handout #25)
    - Catastrophic Reactions & Sundowning (Handout #27)
    - Help with Inappropriate Sexual Behavior (Handout #30).
3. Consider the following Patient Handouts
    - Suggestions for Communication (Handout #5)
    - Activities to Encourage (Handout #8)
    - Activities to Avoid (Handout #9)
    - Catastrophic Reactions & Sundowning (Handout #21)
    - Help with Inappropriate Sexual Behavior (Handout #23).
4. Keep and/or remove the patient away from situations and individuals that upset the patient.
5. Speak with a reassuring and gentle tone to the patient. It can help to defuse a situation by calming the person.
6. Approach an agitated person slowly and calmly from the front. Caregivers should tell the person what they are going to do and try not to startle them.
7. Use non-threatening postures when dealing with an agitated patient. Standing over a patient who is seated or in bed can be frightening and may provoke anger. Caregivers should bend from the knees, kneel or sit down so that they are at the patient's eye level.
8. Redirect the person with questions about the problems and gradually turn their attention to something unrelated and pleasant. Provide different activities. Go to another room and leave the current situation for a while.
9. Establish a calm, quiet environment.
    - Use soft lighting and calm colors such as tan, peach, pale blue or green.
    - Use carpeting to absorb sound (avoid throw rugs since patients can trip.)
    - Add soft music.
    - Offer a favorite soothing item, such as a cup of tea, blanket, snack, or try a soothing behavior like a walk.

    Avoid the following:

    - Abstract/noisy designs which can be confusing or disturbing
    - Mirrors (outside dressing areas/bathrooms) which may be confusing
    - Loud telephone bells or paging systems, frequent re-design or room changes
10. Avoid arguing and trying to reason while the patient is agitated. Arguing almost always causes the agitation. It is almost always impossible to reason with a person with dementia.
11. Be flexible. Medications may be given within one hour before or after scheduled time and a bath is not required daily.
12. Use touch judiciously. Sometimes a touch or a hug can be comforting to an agitated person, but for another it could be provoking.
13. Present patient to m-CCRP Team for evaluation.

© 2024 Khan BA et al. *JAMA Network Open*.

14. Consider medical intervention.

Instruct caregiver about REPETITIVE QUESTIONS:

1. Consider the following Caregiver Handouts
   - Help with Wandering (Handout #23)
   - More help with Wandering (Handout #24)
   - Help with Verbal Noises (Handout #25)
   - Help with Repetitive Behaviors or Words (Handout #26)
2. Consider the following Patient Handouts
   - Help with Wandering (Handout #20)
3. Attempt to ignore consistently repeated questions. This technique can work with some patients, but it will upset others who may become angry because they were not answered.
4. Understand that sometimes the patient may be unable to express what is actually worrying him or her. For example, if a patient continues to ask for someone who is dead, he or she may be trying to express that they feel lost.

Caregivers can:

- React to this emotional aspect, explaining that they will take care of the patient.
- Distract the patient, either by asking him/her about something related or unrelated.

5. Occasionally, the patient responds only to agreement by caregivers or being distracted by a lie, for example, telling the patient a deceased friend or relative will be coming later or by simply stating, “No, they cannot come today.”

Instruct caregiver about REPETITIVE TASKS:

1. Implement for a patient who is continually repeating a task:
   - Give a new or specific task to perform. It is important that the caregiver does not appear to pressure the patient or sound upset; in such situations, a catastrophic reaction can be precipitated.
   - Providing positive attention for more appropriate behaviors will encourage these behaviors.
   - Identify tasks that are repetitive and engage the patient in these tasks (e.g., folding laundry, sorting socks, organizing a toolbox, etc.).

Instruct caregiver about Wandering

1. Consider the following Caregiver Handouts:
   - Help with Wandering (Handout #23)
   - More Help with wandering (Handout #24)
2. Consider the following Patient Handouts:
   - Help with Wandering (Handout #20)
3. Install locks that are unfamiliar and difficult to operate and are out of the patient’s reach so that he cannot go outside unsupervised. Secure all means of exit in addition to doors. Patients may climb out second story windows, so secure them too.
4. Surround the patient with familiar things (i.e., pictures of his/her family, a throw blanket, and cuddly animals).
5. Provide frequent reassurance about where they are and why they are there; a patient will often forget that he or she is supposed to be in a particular place.
6. Involve the patient who is going to be moved in planning the move. Visit the new setting before hand, if he/she is able to understand what is going on.
   - With a more severely confused patient, it may be easier not to introduce them gradually, but to make the move as quickly as possible and without any fuss.
7. Give him/her a pocket card, if the person is can still read, understand and follow instructions. Instructions on the card should be simple and may include the following:
   - ‘Stay calm and don’t walk away’
   - ‘Call home’ and #000-000-0000.
   - For a shopping trip, ‘Ask to be shown the menswear department and stay there. I will find you’.
8. Implement for aimless wandering:

© 2024 Khan BA et al. *JAMA Network Open*.

- Exercise may help to reduce restlessness. Walk daily.
- Try to redirect him/her rather than directly confront the patient.
- Walk with the patient and then lead him/her around a big circle.
- Create an environment that calms the person.

9. Implement for incessant wandering:
   - Sit down with the patient and help him/her to put their feet up. Continuous walking causes the patient's feet to swell. He/she may sit still as long as the caregiver sits still.
10. Implement for restless wandering:
    - Give him/her some active task like dusting or stacking boots.
11. Consider physical devices to restrain a person in a chair or bed. Either a chair or a Posey restraint may help to keep a person still and safe long enough for you to take a bath or fix supper. Never leave a person alone in the house while he is restrained, because of the possibility of a fire.
12. Present patient to Care Plan Team for evaluation.
13. Consider medication intervention.

Instruct caregiver about SHADOWING:

1. Tolerance for this behavior will keep the patient and you calm. Find other people to help with the person so you can get away for some respite, preferable on a regular basis.
2. Childproof doorknobs or the bathroom door may help give you a few minutes of privacy.
3. Redirect the patient with simple tasks that the person can still do.

Caregiver:

4. When the wandering behavior is more than can be managed or when a person cannot be kept safely in a home setting, and the caregiver has done all he or she can do, the caregiver will need to consider plans for institutional care for the patient.

Instruct Caregiver about DELUSIONS/HALLUCINATIONS

1. Consider the following Caregiver Handouts:
   - Help with Paranoia (Handout #28)
   - Help with Hallucinations (Handout #29)
   - Help with Inappropriate Sexual Behavior (Handout #30)
2. Consider the following Patient Handouts:
   - Help with Hallucinations (Handout #22)
   - Help with Inappropriate Sexual Behavior (Handout #23)
3. Avoid denying the person's experience or directly confronting him/her or arguing with the person.
4. Listen or give a noncommittal answer. Do not play along with a delusion or a hallucination if possible.
5. Try to distract the person.
6. Attempt to touch the person physically, as long as they don't misinterpret your touch as an effort to restrain him/her.

Instruct Caregiver about PARANOIA:

1. Consider the following Caregiver Handouts:
   - Help with Paranoia (Handout #28)
   - Help with Hallucinations (Handout #29)
   - Help with Inappropriate Sexual Behavior (Handout #30)
2. Consider the following Patient Handouts:
   - Help with Hallucinations (Handout #22)
   - Help with Inappropriate Sexual Behavior (Handout #23)
3. Understand that this is not behavior the patient can control.
4. Do not confront the patient or argue about the truthfulness of the complaint.
5. Distract the patient with other activities.
6. Consider medication intervention.

© 2024 Khan BA et al. *JAMA Network Open*.

Psychiatric Consultation

1. After failed non-pharmacological intervention and clinically significant problem (e.g. the hallucinations or paranoia leads to agitated behaviors).
   a. Symptoms present at least 1 month's duration.
   b. If mood episodes have occurred concurrently with the delusions, hallucinations or paranoia, their total duration has been brief relative to the duration of the delusional, hallucinatory or paranoid periods.
   c. The disturbance is not due to the direct physiological effects of a substance or illness.

2. If the patient is at risk of harming himself or others or has significant functional impairment from delusions, hallucinations, and paranoia (e.g. refusing to eat because believes food is poisoned or unable to leave the house because of severe paranoia).

© 2024 Khan BA et al. *JAMA Network Open*.

Protocol for Care – LEGAL & FINANCIAL

- Refer the patient to Social Services at their local Health Care Facility. Provide the Social Services Phone Number and ask if they need help to set up the appointment.

- If they ask for help, set up the appointment with the local social services.

© 2024 Khan BA et al. *JAMA Network Open*.

Protocol for Care – COMMUNICATION

Instruct caregiver in COMMUNICATION:

1. Consider the following Caregiver Handouts:
    - Communicating with your Loved One (Handout #4)
    - Suggestions for Communication (Handout #5)
    - Understanding Nonverbal Messages (Handout #6)
    - Nonverbal Messages (Handout #7)
2. Consider the following Patient Handouts:
    - Communicating with your caregiver (Handout #4)
    - Suggestions for Communication (Handout #5)
3. Speak slowly and wait for the patient to respond.
4. Confirm the patient is able to hear you.
5. Lower the pitch of your voice.
6. Eliminate distracting noises or activities in the surrounding area.
7. Ask only one simple question at a time and repeat the same way if necessary.
8. Ask the patient to do only one task at a time and give simple instructions.
9. Help the patient to find the correct word, if he/she is struggling to find a word.
10. Have the patient point to an object, if they can't think of the name.
11. Repeating the patient's last words may get them started again.
12. Speak in simple, short sentences and/or remove the patient away from situations and individuals that upset the patient.
13. Check the patient's comfort frequently if they cannot talk:
    - Make sure their clothing is comfortable
    - Make sure the room is warm or cool enough.
    - No rashes or sores on their skin
    - Take to the toilet on a regular schedule
    - Make sure they are not hungry or sleepy.
14. Recognize nonverbal clues:
    - Remain calm and supportive.
    - Smile and touch the person to express affection
    - Look directly at person to confirm if they are paying attention.
    - Demonstrate specific activities such as dressing.

© 2024 Khan BA et al. *JAMA Network Open*.

Protocol for Care – MOBILITY: BALANCE/FALLS

Instruct caregiver in BALANCE/FALLS:

1. Consider the following Handouts:
   - Help with Balance and Walking (Handout #19)
   - About Falling and Injuries (Handout #20).
2. Consider the following Patient Handouts:
   - Help with Balance and Walking (Handout #16)
   - About Falling and Injuries (Handout #17).
3. Put away scatter rugs, pad steps, tack down rug edges.
4. Provide secure leather sole shoes.
5. Remove all clutter from traffic areas.
6. Place person in the front seat of the car with seatbelt.
7. Do not move the patient after a fall.
8. When a person falls:
   - Remain calm
   - Check for injury or pain
   - Avoid precipitating a catastrophic reaction
   - Watch the person for signs of pain, swelling, bruises agitation, or increased distress; call the doctor if any of these symptoms appears or if you think there is any chance that he hit his head or otherwise hurt himself.

© 2024 Khan BA et al. *JAMA Network Open*.

Protocols for Personal Care- BATHING/DRESSING/MEALS/TOILETING

Instruct caregiver in BATHING:

1. Consider the following Caregiver Handouts:
   - Personal Care (Handout #11)
   - Bath Time (Handout #14)
2. Consider the following Patient Handouts:
   - Bath Time (Handout #11)
3. Maintain the patient's independence by encouraging participation in ADL's, e.g. wash face.
4. Maintain the person's daily routines when possible, and simplify the process.
5. Following a regular routine, done the same way at the same time i.e. shower versus bath, morning versus evening.
6. Get the assistance of a male/female or attendant to assist with personal care.
7. Talk to the patient with a reassuring and calm voice. Give instructions one-step at a time.
8. Partial baths or sponge baths are adequate daily hygiene.
9. Assistive appliances make bathing easier i.e. bath seat, hand held showerhead, and rubber mat.
10. Place only 2-3 inches of water in the tub.
11. Check the skin for reddened areas; breaks in the skin, rashes, and sores.
12. Install a flexible shower-head.

Instruct caregiver in DRESSING:

1. Consider the following Caregiver Handouts
   - Personal Care (Handout #11)
   - Getting Dressed (Handout #12)
   - Clothing Ideas (Handout #13)
2. Consider the following Patient Handouts
   - Getting Dressed (Handout #10)
3. Arrange outfits with all pieces together, i.e. shirt with pants, blouse with skirt.
   - Eliminate belts, scarves, sweaters, ties, and other accessories that are often confusing and likely to be put on wrong.
4. Remove clothing that will not be used and simplify the closet or dresser. Too many clothes are often confusing.
5. Lay out a clean outfit for the patient. Laying out clothes in the order in which he/she puts them on may also help.
6. Purchase clothing with elastic waist-bands, replace buttons with Velcro tape, slip on shoes, and loose-fitting clothing.
7. Select clothing that is washable and that doesn't need ironing. For women short ankle socks are recommended versus stockings (full or knee high).

Instruct caregiver in MEALS

1. Consider the following Caregiver Handouts:
   - Personal Care (Handout #11)
   - Mealtimes (Handout #16)
   - More help at Mealtimes (Handout #17)
2. Consider the following Patient Handouts:
   - Mealtimes (Handout #13)
   - More help at Mealtimes (Handout #17)
3. Use plastic tablecloth or placemats when the person develops problems with coordination and becomes messy. When the patient starts to use fingers instead of silverware, serve finger foods.
4. Use heavy dishes with sides and/or a plate guard to prevent food from being pushed from the dish.
5. Use smocks over clothing.
6. Try a convalescent feeding cup (spill proof cup for children) when drinking from a cup.

Instruct caregiver in TOILETING/INCONTINENCE (both urine and bowel):

© 2024 Khan BA et al. *JAMA Network Open*.

1. Consider the following Caregiver Handouts:
    - Personal Care (Handout #11)
    - Toileting (Handout #15)
2. Consider the following Patient Handouts:
    - Toileting (Handout #12)
3. Give adequate fluids (6-8 cups/day) before 7:00 PM to decrease nighttime voiding.
4. Providing a commode and/or urinal to decrease the distance the patient has to travel.
5. Provide a chair and a bed that is easy for the person to get up and out of.
6. Leave a night light on, remove throw rugs, and use slippers that are not slick-soled or floppy.
7. Remove the lock from the bathroom door.
8. Schedule bathroom trips every 3-4 hours.
9. Observe for non-verbal clues that the person has to use the bathroom (i.e., restlessness, picking at clothing, etc.).

© 2024 Khan BA et al. *JAMA Network Open*.

Protocol for Care – SLEEP DISTURBANCE

Step 1.

Instruct Patient/Caregiver about SLEEPING:

1. Consider the following Caregiver Handouts:
   - Help with Sleeping (Handout #22)
   - Help with Wandering (Handout #23)
   - More help with Wandering (Handout #24)
   - Help with Verbal Noises (Handout #25)
2. Consider the following Patient Handouts:
   - Help with Sleeping (Handout #19)
   - Help with Wandering (Handout #20)
3. Implement a bedtime routine:
   - Brush teeth
   - Comb hair
   - Bath
   - Soft music
   - Conversation
4. Keep patient form napping during the day so he/she will be tired at night. Try keeping him occupied, active, and awake in the daytime.
5. Plan a regular activity program:
   - Long walk
   - Car ride
6. Take the person to the bathroom before bedtime.
7. Make sure the sleeping arrangements are comfortable:
   - Room is neither too warm nor too cool
   - Bedding is comfortable. Quilts are less likely to tangle than blankets and sheets.
8. Have the patient sleep in a lounge chair or on a sofa, if they will not sleep in a bed.
9. If all else fails, consider medication intervention. The caregiver needs to get rest.

Step 2.

1. If no sleep improvement in two weeks, initiate pharmacologic therapy for sleep.
2. Start Melatonin 5 mg by mouth daily after consultation with the PCP and m-CCRP Team.

Step 3.

1. If no improvement in 2-3 weeks, increase the Melatonin dose to 10 mg by mouth daily.
2. **Consider adding Trazodone 25-50 mg by mouth at nighttime.**
3. **Consider referring to a sleep specialist.**

© 2024 Khan BA et al. *JAMA Network Open*.

Protocol for Care – Pain Management

1. Review and confirm diagnosis and potential contributing causes.
2. Assess pain on every visit through PEG pain scale.
3. Evaluate for depression as possible contributor to chronic pain.
4. Provide patient/caregiver the pain related handouts for education on pain and pain management.
5. Provide education on potential side effects of pain medicines.
6. Encourage regular exercise, walking, and physical activity.
7. Work with patient's primary care provider for pain management through the pharmacologic pain protocol and the analgesic algorithm.
8. Discuss with patients primary care to consider treatment with antidepressant for depression. Consider supportive counseling or group therapy for patient and/or caregiver.
9. Consider alternative modalities such as acupuncture, massage, heat, cold, relaxation and distraction techniques.
10. If patient is on narcotics, initiate prophylactic bowel regimen and monitor for constipation.
11. Consider Pain Consult for further evaluation and management of chronic pain.
12. Consider Physical Medicine and Rehabilitation Consult for further evaluation and management of chronic pain.
13. Consider Physical Therapy Consult for treatment of pain, rehabilitation of impaired range of motion, specific muscle weakness, etc

© 2024 Khan BA et al. *JAMA Network Open*.

Protocol for Care – STRESS

Assess for:

- Poor insight of situation
- Unsatisfactory caregiver-care receiver relationship
- Competing roles
- Social isolation
- Insufficient leisure
- Unrealistic expectations of caregiver place on themselves and/or by others
- Access or accept help
- Insufficient resources

Instruct patient/caregiver about STRESS:

1. Consider the following Caregiver Handouts:
   - For the Caregiver (Handout #1)
   - Guidelines for Coping (Handout #2)
   - Looking on the Bright Side (Handout #3)
   - Depression (Handout #21)
   - Help with Sleeping (Handout #22)
   - Help with Verbal Noises (Handout #25)
2. Consider the following Patient Handouts:
   - For taking care (Handout #1)
   - Guidelines for Coping (Handout #2)
   - Looking on the Bright Side (Handout #3)
   - Depression (Handout #18)
   - Help with Sleeping (Handout #19)
3. Instruct caregiver to:
   - Identify all possible sources of volunteer help
     a. Family (siblings, cousins)
     b. Friends, neighbors
     c. Church, community groups
   - Improve coping skills (time management, stress management)
     a. Energy
     b. Beliefs
     c. Commitments, health, social skills, social support, material resources
   - Obtain positive feedback to lower levels of burnout, i.e., attend support group
   - Need consistent social support, i.e., set a consistent schedule for respite care, with a minimum of 4 hours a week.
   - Ease into home health or companion services for persons with severe cognitive impairment.

© 2024 Khan BA et al. *JAMA Network Open*.

Acute Care Reduction Domain – Current ABC Medical Home Patients at Indiana University

- Research staff informs m-CCRP care coordinator of admission or emergency room visit.
- Care coordinator reviews medical record for reason for admission or ED visit
- Care coordinator will text the ACE nurse practitioner (for patients >65 years of age) and Registered Nurse to inform them that this is an m-CCRP patient, request an ACE consult, and inform m-CCRP will follow at discharge.
- Care coordinator monitors patient care during inpatient stay and discusses/intervenes with ACE and inpatient teams, as indicated.
- If patient is discharged home, nurse practitioner notifies m-CCRP care coordinator to schedule home visit within 72 hours of discharge.
- At first home visit, m-CCRP care coordinator completes:
    - History of problems or questions since discharge
    - Review discharge summary and recommendations
    - Review reason for admission/ED visit
    - Discuss alternative ways to avoid inpatient stays and ED visits
    - Reconcile Medications
    - Functional status
    - CAM
    - MMSE/MOCA, if CAM negative
    - HABC Caregiver Monitor
    - HABC Self Report Monitor
    - Provides caregiver delirium handouts
- Present patient at weekly m-CCRP Team Meeting and complete Root Cause Analysis.
- Recommend local memory clinic consult for MCI if not previously evaluated.
- Follow up will be dependent on diagnosis
- Admitting Nurse Practitioner will update patient's PCP and m-CCRP Team, and m-CCRP patient, of visit and follow up plans.

© 2024 Khan BA et al. *JAMA Network Open*.

Protocol for Care – DELIRIUM: PREVENTING DELIRIUM IN THE HOSPITAL

1. To ensure continuity of patient care when an m-CCRP patient is hospitalized, the following steps should be taken:
   - M-CCRP Care Coordinator will notify the ACE/geriatrics team for patients who are ≥65 years of age, if available in the hospital, of the patient's admission and will request a consult.
   - If the ACE team is unable to evaluate the patient or the patient is <65 years of age, the Care Coordinator will notify the inpatient team of the patient's admission and relevant diagnoses.
   - The Care Coordinator will inform the ACE team or inpatient team of the patient's increased risk of developing delirium and any previous history of delirium.
   - The Care Coordinator will provide the ACE team or inpatient team with patient's medication list, information about patient's living situation, specific concerns the Care Coordinators has about the patient and any other information that might assist the ACE team or the inpatient staff in caring for the patient.
   - The Care Coordinator will request that the ACE team or inpatient staff provide him/her with notification of patient discharge, specific concerns about this patient at the time of discharge and a discharge summary.

Instruct Caregiver about DELIRIUM:

2. Consider the following Caregiver Handouts:
   - Help with Delirium in the Hospital (Handout #31)
   - Help with Delirium at Home (Handout #32)

© 2024 Khan BA et al. *JAMA Network Open*.

Protocol for Care – DELIRIUM: POST-HOSPITALIZATION

Instruct caregiver in DELIRIUM:

1. Consider the following Caregiver Handouts:
   - Help with Delirium in the Hospital (Handout #31 & 32)
   - Help with Delirium at Home (Handout #33 & 34)

2. Initiate Medication Adherence Protocol within 72 hours after hospital discharge

© 2024 Khan BA et al. *JAMA Network Open*.

MEDICATION ADHERENCE PROTOCOL

Medication Reconciliation is defined by the JCAHO as the "process of comparing a patient's medication orders to all of the medications that the patient has been taking. This reconciliation is done to avoid medications errors such as omissions, duplications, dosing errors, or drug interactions…" and may well prevent re-hospitalizations. Medication reconciliation will be conducted on each visit by the m-CCRP Care Coordinator

- The Care Coordinator will review all medications the patient is taking, including OTCs and herbal products. With the patient's and/or the patient's caregiver's permission, all expired or unused medications will be removed.
- The Care Coordinator will review the admission medication list, discharge medication list and the patient's actual medications.
- Any medication discrepancies will be corrected. If the Care Coordinator is unsure of what action to take to correct a medication discrepancy, he/she will contact the patient's Primary Care Provider ("PCP") as soon as possible.
- If a medication discrepancy is noted but cannot be corrected immediately, the Care Coordinator will note the discrepancy on the patient's medication list. Once the Care Coordinator obtains clarification about the discrepancy, the Care Coordinator will call the patient and the patient's caregiver to explain how the medication is to be taken.
- The Care Coordinator will mail an updated medication list to the patient and the patient's caregiver within 24 hours after reconciliation is completed.

3. Immediately after completing the medication reconciliation, the Care Coordinator will assist the patient and the patient's caregiver in developing an effective medication management system. This will include:
    - Educating the patient and the patient's caregiver about the need for each medication and the importance of taking as prescribed.
    - If a patient is unable to pay for any medication, either assisting the patient in obtaining assistance for such medication or referring the patient to someone who is able to provide such assistance
    - Providing education about what to do if a medication discrepancy is found.
    - Educating the patient and the patient's caregiver to take the medication list to each provider appointment and have it updated when there are changes.
4. The Care Coordinator will provide education to both the patient and the patient's caregiver about the "red flags' that may indicate the patient's condition is worsening, how to respond and when to contact the Care Coordinator and/or the patient's PCP.
5. Within 48 hours after the medication reconciliation is completed, the Care Coordinator will be responsible for updating the patient's outpatient medication list in the Electronic Medical Record, if needed.

© 2024 Khan BA et al. *JAMA Network Open*.

Taking Care Of Yourself

You cannot take care of anyone else if you don't take care of yourself first.

Remember to :

- Get plenty of rest.
- Drink 8 glasses of water a day and eat right.
- Take a walk everyday and include your loved one.
- Keep your prescriptions filled and take your medications as prescribed.
- Keep your own doctor's appointments. Plan ahead to arrange transportation and get someone to stay with your loved one.
- Make time for yourself! Take regular breaks from caregiving. 8 hours a week is ideal.

Don't put your own health on the back burner

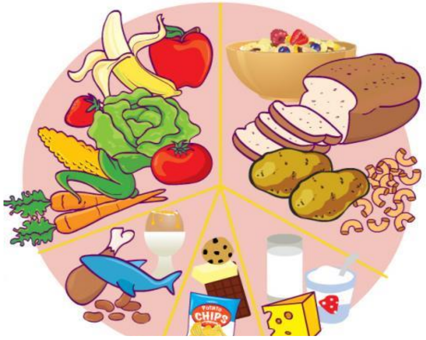

© 2023 Khan BA et al. *JAMA Network Open*.

Guidelines for Coping

- Be realistic about all that you can do.
- Don't do everything for everybody. Let others do as much for themselves as possible.
- Take it one day at a time.
- Communication is essential.
- Ask for help - this is a sign of strength.

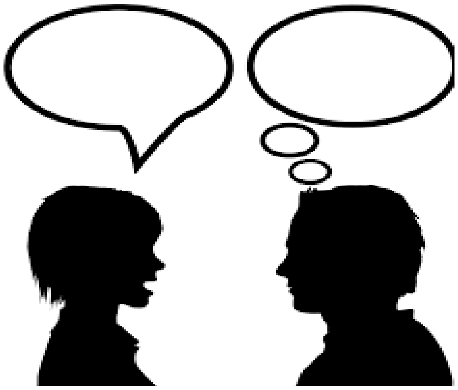

- Tell your family and friends how you feel, what it is like caring for a loved one with dementia.
- Plan ahead. Think about the future, things that need to be taken care of. Make the plans.
- Tell your family and friends about wills, power of attorney, health care representative, living will - but don't dwell on it.

© 2023 Khan BA et al. *JAMA Network Open*.

Looking on the Bright Side

Important caregiver responses are those of:

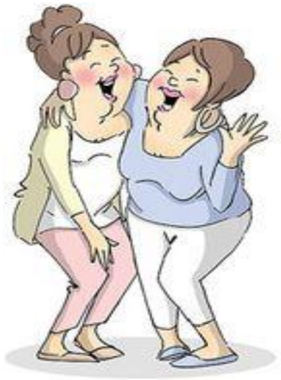

- Joy
- Warmth
- Closeness
- Satisfaction
- Laughter
- Happiness

Many family members feel good about providing care to a loved one.

Don't forget to look at the bright side.

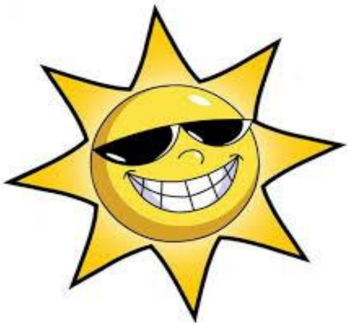

© 2023 Khan BA et al. *JAMA Network Open*.

Communicating with your loved one

- Identify yourself.
- Speak slowly and softly.
- Use positive facial expressions.
- Talk in a noise free, non distracting place.
- Move slowly and touch gently on the arm or hand to get their attention.
- Converse in a social and non threatening manner.
- Use short, specific, familiar words and simple sentences.
- Give one direction or ask one question at a time. -"Are you in pain?"
- Allow plenty of time for them to respond.
- If there is no response, repeat exactly what you said. -"Are you in pain?"

© 2023 Khan BA et al. *JAMA Network Open*.

Suggestions for communication

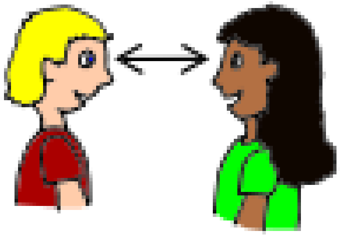

- Maintain eye contact.
- Do not take negative things they say personally.
- Watch for signs of restlessness or withdraw[illegible] away or frowning.
- Tell them you understand it's not a good time and you'll try again later.
- If they can't find the right word, cue their associations. Pointing to his mouth, he says" it's like i eat with it...in here." You say, "Your teeth."
- People who live "in the moment" need reassurance in that moment.
- Remember persons with dementia can read words long after they stop understanding the written thought.
- Always treat your loved one with dignity and respect.

© 2023 Khan BA et al. *JAMA Network Open*.

Understanding non verbal messages

Facial Expressions

Your loved one may no longer be as lively in their facial expressions as they once were. Often their face is rigid, but if you look closely and carefully, you may be able to see small changes which may give you a clue to what they're feeling.

For example, if they are happy, the lower eyelids are crinkly, cheeks are raised, the mouth smiles. If they are sad, the face droops, and the mouth may tremble. Look for other nonverbal clues to interpret what they are trying to say.

The EYES are the window to the soul. The emotions and intelligence are sometimes expressed in the eyes of those who can no longer talk or understand tell us the that there is still the soul of a human being present who deserves caring and respect.

Just because they cannot speak doesn't mean they are not trying to tell us something.

Eyes may show that your loved one is still aware. Look for widening or narrowing of the eyes, eye movement , tension in the lids, and tears in the eyes.

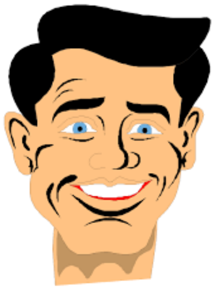

© 2023 Khan BA et al. *JAMA Network Open*.

Nonverbal Messages

Posture which indicates your loved one is non receptive.

- Your loved one backs away
- Turns their head or body away
- Walks away
- Frowns
- Avoids eye contact
- Pulls away from you
- Shrinks away from being touched
- Tells you to go away

If your loved one is non receptive and you continue to interact with them, this may lead to catastrophic reactions.

Vocalizations.

When words fail, listen for clues in the speed, pitch, and volume in your loved ones vocalizations.

A loud tone is more likely to cause concern than a quiet one.

Speaking at a fast rate indicates that they may be losing self control or feeling anxious.

Wait for your loved one to feel calmer before continuing or approaching again.

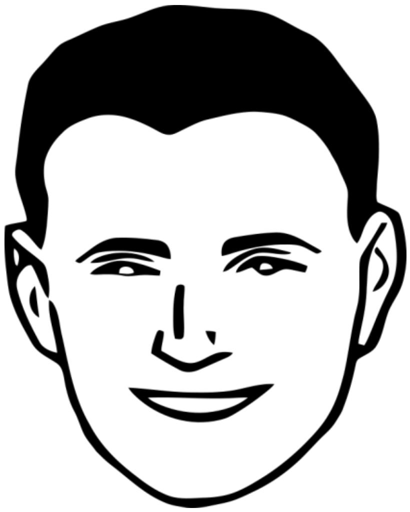

© 2023 Khan BA et al. *JAMA Network Open*.

Help with Exercise

Being active may lead to good sleep and decreased agitation, stress, or moodiness for both you and your loved one. Be sure to get a doctor's approval before you/your loved one starts an exercise plan.

TO INCREASE PHYSICAL ACTIVITY, YOU MAY:

- Turn on your favorite music. Dance by yourself or with a partner for at least 10 minutes or longer at a time.
- Try to follow the routine on a free television exercise program "SIT and BE FIT" on your local PBS station.
- Go for a walk in your neighborhood. Again, try to walk for at least 10 minutes.
- Ride a stationary exercise bike for at least 10 minutes at a time. Be sure to start slowly, then gradually build up speed.
- Attend a local exercise classes designed for seniors.
- Try to do stretching, balance and strength building exercises. The National Institute of Aging (NIA) offers a free exercise book and videos, which can be ordered.

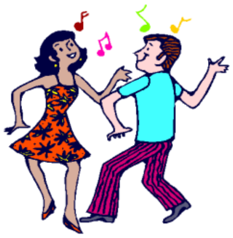

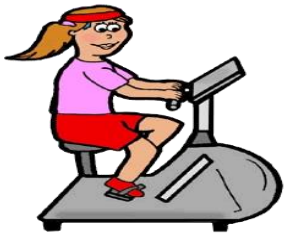

© 2023 Khan BA et al. *JAMA Network Open*.

Activities to Encourage

- Pleasant current events
- Exercise
- Music and movement
- One-to-one attention
- Reminiscence
- Creative activities
- Food
- Helping around the house

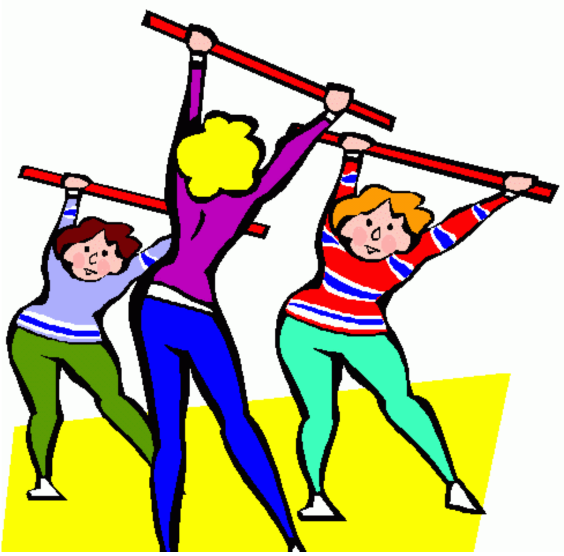

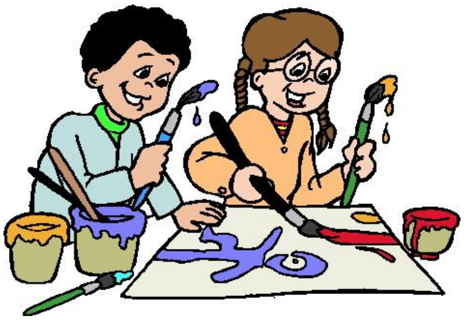

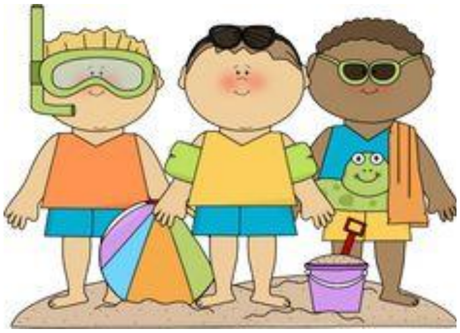

© 2023 Khan BA et al. *JAMA Network Open*.

Activities to Avoid

- Leaving the TV or radio on all day.
- Noisy, confusing programs or entertainment.
- Long programs.
- Activities that drastically change your loved one's routine.
- Activities that require a lot of preparation and explanation.
- Busy work.
- Things that require memory.
- Childish activities.
- Too much activity or too many visitor.

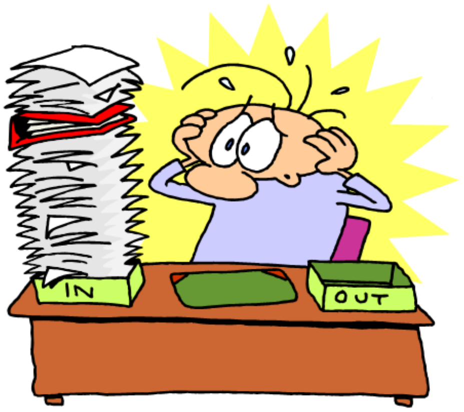

© 2023 Khan BA et al. *JAMA Network Open*.

Personal Care

- Figure out an easy routine and stick to it.
- Remember that light, color, and noise may affect your
- loved one.
- As much as possible, keep things the same.
- Let your loved ones take care of themselves as much as they can.
- Don’t try to explain or reason.
- Don’t argue.
- Keep things calm.
- If your loved one’s personal care becomes difficult, ask for help.

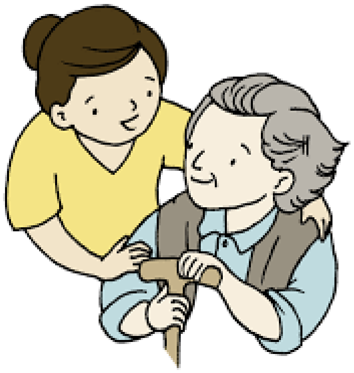

© 2023 Khan BA et al. *JAMA Network Open*.

Getting Dressed

- Get dressed in one place, undressed in another.
- Lay clothing out in the order it is put on.
- Pull curtains and close doors for privacy.
- Your loved one may undress if room’s too warm.
- They may not want to undress if it's too cold.
- Watch for a sign that you need to help.
- Help more when the time comes.
- Don’t argue or force changing clothes.
- Be flexible and ready to try a new way.

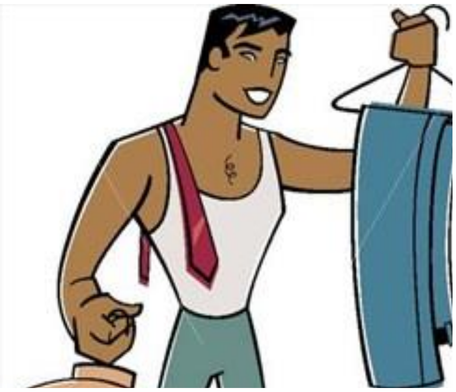

© 2023 Khan BA et al. *JAMA Network Open*.

Clothing Ideas

- Keep the closet neat.
- Store out of season clothes somewhere else, if possible.
- Hang all parts of the outfit together.
- Lay clothing out in the order it's put on.
- Ask someone who sews to replace buttons with Velcro.
- Avoid clothes that go over the head.
- Use clothes that fasten in front. If they remove clothes at the wrong times, use clothes that fasten in back.
- Slip-on shoes with rubber on the bottom are safe and easy.
- Use clothing that is easy and comfortable; Sweat pants are easy and comfortable.
- Wants to wear the same clothes every day? Get outfits that look the same.

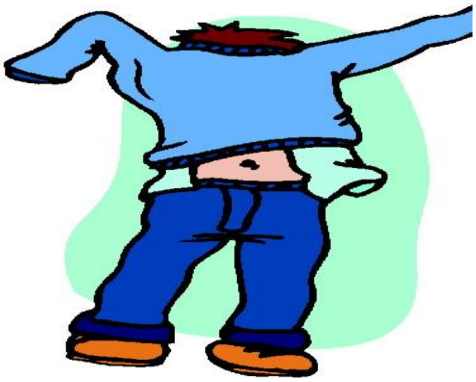

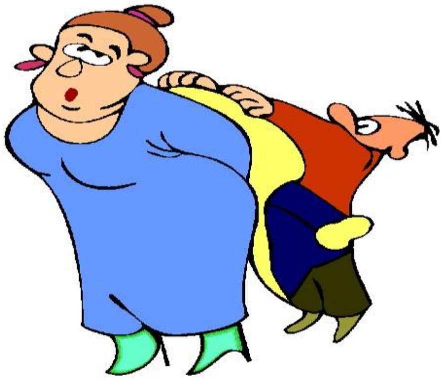

© 2023 Khan BA et al. *JAMA Network Open*.

Bath Time

- Keep bath time the same every day or week.
- Keep bath area safe:
  - -no throw rugs
  - -use non-slip mats
  - -provide enough light
  - -turn down
  - -temperature on water heater
- Make sure bath is warm and have everything ready.
- Don't ask if your loved one is ready for a bath. Say "Your bath is ready now."
- For privacy, close curtains and bathroom door.
- Don't embarrass your loved one.
- Give one instruction at a time. For example: "pick up the soap."
- Allow your loved one to do as much as possible.
- Don't argue, fight, or rush.
- Never leave them alone.
- Allow time for relaxation.

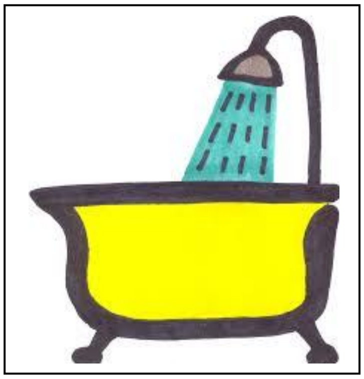

© 2023 Khan BA et al. *JAMA Network Open*.

Toileting

- Lead them to the toilet and help them get started.
- Try to predict when your loved one will need the toilet.
- Use the toilet regularly, for example every two hours.
- Try to avoid or prevent accidents.
- Use the toilet before and after meals and before bed.
- Provide good lighting to and in the bathroom.
- Look for patterns of where and when accidents occur.
- Would a commode by the bed at night help?.
- Give them 6-8 glasses of water per day, until 7:00 pm.
- Limit caffeine and avoid citrus juices and other bladder irritants
- Avoid skin breakdowns. Keep your loved one clean and dry.

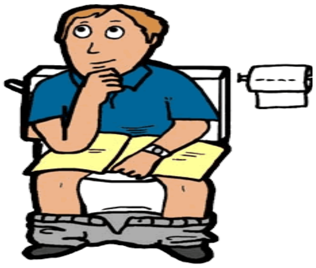

© 2023 Khan BA et al. *JAMA Network Open*.

Meal times

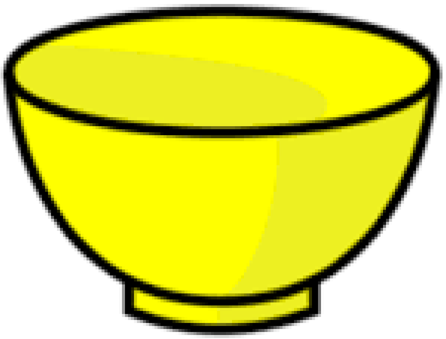

- Offer meals at regular times.
- Try soft, relaxing music at mealtimes.
- Keep distracting things off the table.
- Use bowls rather than plates.
- Try one food, one bowl, one utensil at a time.
- Don't use plastic utensils.
- Serve "finger foods."
- Allow your loved ones to feed themselves.
- Give verbal clues and reminders:
  - -Open your mouth.
  - -Chew your food.
  - -Have another bite.
  - -Drink the water.
- Allow enough time to eat.

© 2023 Khan BA et al. *JAMA Network Open*.

More Help at Meal times

- Remember your loved one's eating habits, their likes and dislikes.
- Pay attention to your loved one who's eating.
- Always pay attention to food temperature.
- Give regular drinks to avoid dehydration.
- Alcohol and alcoholic drinks may increase confusion.
- Check for dental and mouth care problems.
- When there's a problem, look for a pattern.
  - -Certain foods causing a problem?
  - -Chewing or swallowing a problem?
  - -Other people causing agitation?
  - -Is something a distraction?

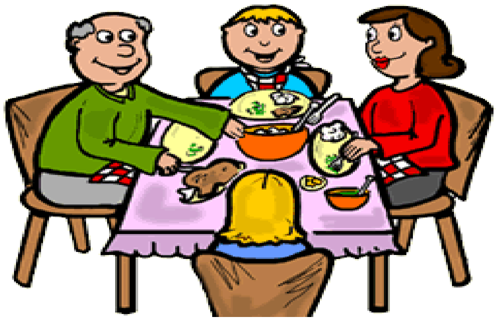

© 2023 Khan BA et al. *JAMA Network Open*.

Dental Care

- Oral health is important. Don’t neglect it.
- Keep a consistent routine for dental care.
- Give simple one-step instructions.
- Show and tell your loved one the behavior you want.
- Use mouthwash that can be swallowed.
- Electric toothbrushes may be confusing.
- Check regularly for mouth sores.
- Check and recheck how dentures are fitting.
- Help your loved one more as the disease progresses.

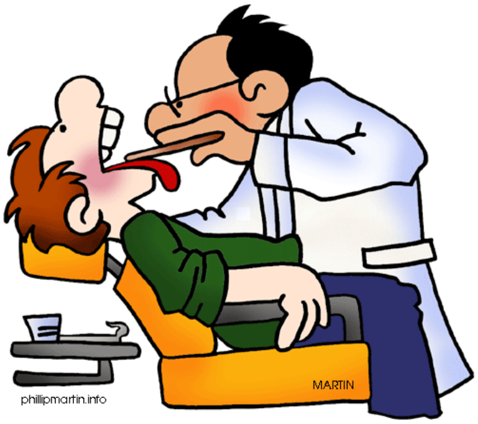

© 2023 Khan BA et al. *JAMA Network Open*.

Help with Balance and Walking

As memory loss progresses, your loved one may become stiff or awkward, may develop stooped or leaning posture or a shuffling walk. This is due to damaged areas of the brain which control muscle movement.

Tell your health care provider about any change in walking, posture, stiffness, repetitive motions, or falling. This may be due to memory loss or there may be a reason that can be treated.

- Put away scatter rugs and pad steps and corners of furniture.
- Chairs and furniture, they lean on should be sturdy.
- Simplify traffic patterns in the home and get things out of the way.
- Tightly install handrails and grabbers.
- Consider which works better. Smooth or sticky shoe bottoms?
- If they will, have your loved one take your arm——hold your arm close to your body.
- If they can't learn to use a cane or walker properly, then it is safer not to use it.

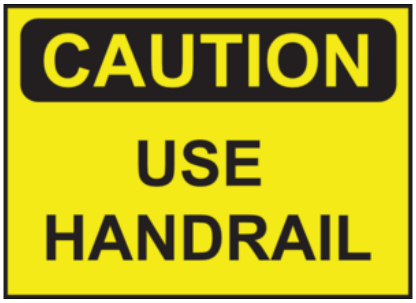

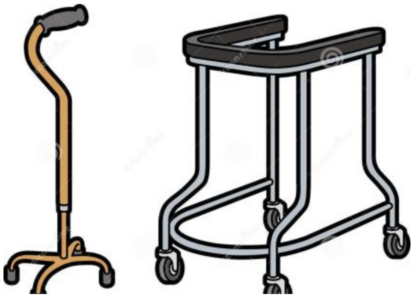

© 2023 Khan BA et al. *JAMA Network Open*.

About Falling and Injuries

Your loved one with memory problems may become clumsy, bump into things, trip, or fall out of bed.
When a person falls:

1. Remain calm and check for pain and injuries you can see.

2. Avoid the start of a catastrophic reaction.

3. Watch for signs of pain, swelling, bruises, agitation, or distress.

4. Call the doctor if these symptoms occur or if they hit their head.

Reasons it's easy to overlook serious injuries:

- Older people are more vulnerable to broken bones from what seems like a minor injury.
- Your loved one continue to use a broken arm or leg.
- People with memory loss may not tell you they are in pain or may forget to tell you they have fallen.

Have a routine and check your loved one for cuts and bruises. Changes in behavior may be your only clue to an injury.

TAKE YOUR TIME

Accidents happen when caregivers and loved one's rush.

© 2023 Khan BA et al. *JAMA Network Open*.

Depression

Depression is very common in dementia and a common response to caregiving. Treating depression can significantly improve one's quality of life.

If you or your loved one go through any of the following, call your health care provider for help:

- Sleeping too much or too little.
- Waking up early in the morning and can't fall back to sleep.
- Eating too little or too much.
- Trouble concentrating.
- Feeling sad all the time and wanting to die.
- Wanting to die.
- Feeling guilty about things in the past.
- Feeling helpless.

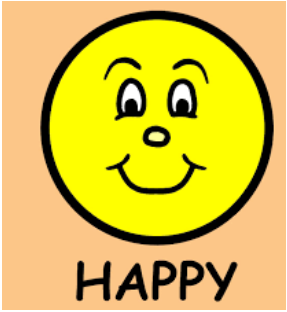

© 2023 Khan BA et al. *JAMA Network Open*.

Help with Sleeping

- Keep previous sleeping and waking habits in mind.
- Being active may lead to good sleep.
- Any change may affect sleep:
  - -Sleeping place
  - -Caregiver change
  - -Different visitors
- Review medications.
- Provide a calm predictable setting.
- Keep a consistent bed-time routine.
- Avoid napping during the day.
- Avoid alcohol, caffeine and heavy spicy foods prior to bedtime.

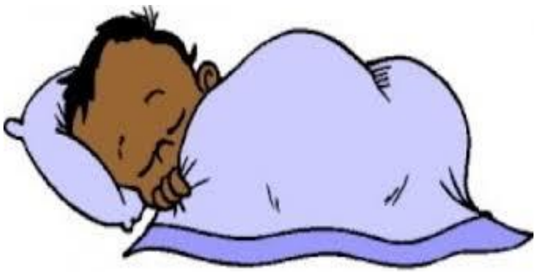

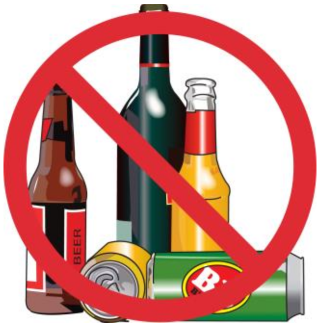

© 2023 Khan BA et al. *JAMA Network Open*.

Help with Wandering

Wandering is a common behavior. There appears to be two kind of wanderers:

The **<u>aimless wanderer</u>** enjoys moving around and looking at things, much like window shopping	The **<u>directed wanderer</u>** has a "mission" with someone to see or some place to go to. This wanderer is usually more difficult to re-direct

- Involve your loved one's in a regular activities and exercise.
- Try to keep your loved one's surrounding familiar and calm.
- Never argue or try to reason.
- Put things out of sight that might remind your loved ones to leave, such as coats, hats, purses and keys.
- Make the door less noticeable.
- Encourage family and visitors to arrive and leave without being noticed.
- Have a plan and a backup plan for what you will do when your loved one starts wandering.

© 2023 Khan BA et al. *JAMA Network Open*.

More Help with Wandering

- If your home is safe and secure, allow them to wander. Install locks that are out of your loved one's reach.
- Develop safe wandering paths inside and outside.
- Try to predict what leads to wandering, then try to change the things that lead to it.
- Consider using monitoring devices that alert you to sound and movement.
- Make sure your loved one has identification on them, such as a bracelet with their name, your phone number, and says "memory impaired."

Is he or she registered with the Safe Return Identification Program? Contact the Alzheimer's Association for details.

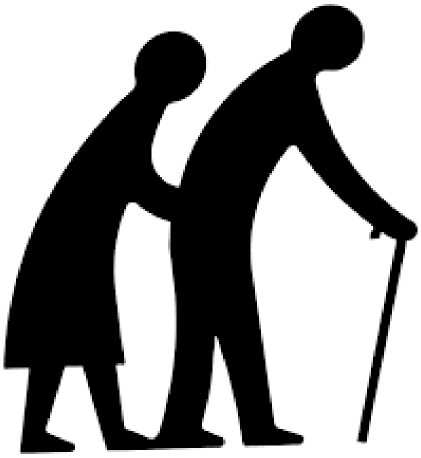

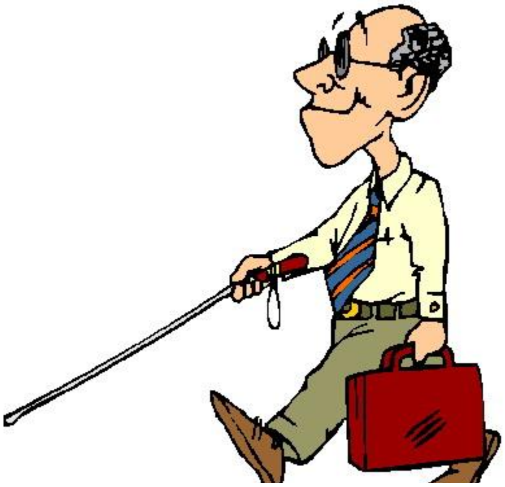

© 2023 Khan BA et al. *JAMA Network Open*.

Help With Verbal Noises

- Create calm, predictable, relaxing surroundings.
- Have a consistent routine.
- Play soft music or relaxing tapes.
- Speak in a soothing tone.
- Use touch, massage, hugs or gently rock your loved one.
- Make sure clothing is comfortable.
- Provide enough meals, drinks, and snacks.
- Make sure they are clean and dry.
- Regularly check the position of their body.
- Check for pain or illness.
- Look for things in the surroundings that might frighten them.
- Ask your doctor or nurse to review medications.

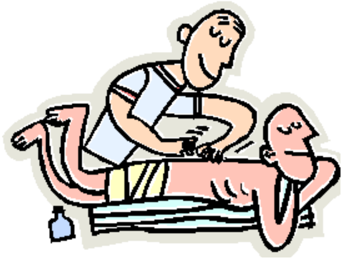

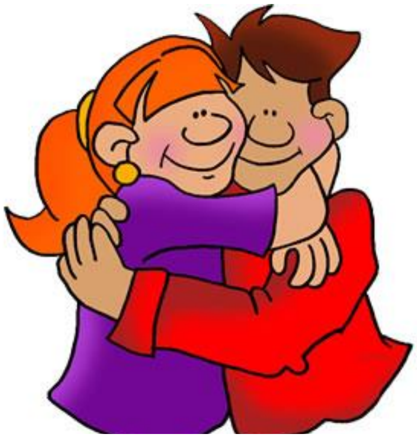

© 2023 Khan BA et al. *JAMA Network Open*.

Help with Repetitive Behavior or Words

Repetition may be:

-caused by the loss of short term memory so your loved one cannot remember they are repeating

-an attempt to regain some control over their behavior

-a method of trying to get attention or pass on information which they cannot express any other way

-an attempt at being social

- Keep a routine and distract your loved one by keeping them busy.
- Sometimes having a note to carry is helpful.
- Video or audiotapes of the family help.
- Redirect with music or food.

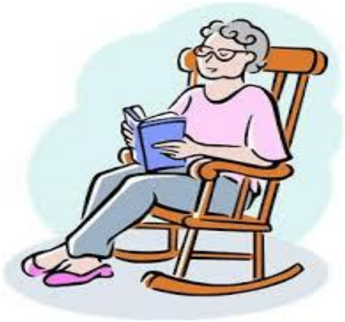

- Answer questions in the same words, in the same tone of voice each time the question is asked.
- Sometimes ignoring the behavior is best, especially if you're getting angry.
- Interrupting a repetitious behavior may upset your loved one.
- Remember that this is a symptom of the disease; they do not do it to annoy you.

© 2023 Khan BA et al. *JAMA Network Open*.

Catastrophic Reactions and Sundowning

SUNDOWNING

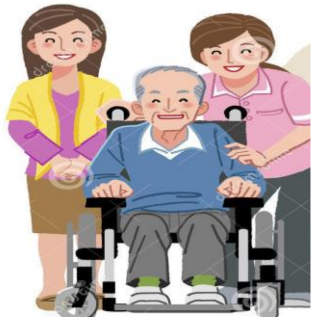

Catastrophic reactions are sudden, unexpected responses that appear to be overreactions to what would normally be viewed as mild stimulation.

Sundown Syndrome: increased agitation and aggressive behavior that occurs late in the day.

**WHY** does my loved one do that?	**THINGS TO TRY** to avoid or smooth out unwanted behavior.
Surroundings are too stimulating, confusing, new.	Simplify the surroundings.
Change in routine or caregiver.	Maintain a steady routine and schedule.
Caregiver is agitated, frustrated, impatient.	Maintain a postive matter-of-fact, calm approach.
Too many demands being made on the loved one	Simplify instructions and do not ask questions that require good memory.
Brain damage from the disease causes misunderstanding of surroundings.	Do not argue. What seems real to your loved one is different than what is real to you.
Side effect of medication.	Suggest a medication review.

© 2023 Khan BA et al. *JAMA Network Open*.

Help with Paranoia

Paranoia is suspicious thinking with feelings that the person is being persecuted, harmed or judged.

Because of memory loss, your loved one may have trouble understanding what is real and what is not.

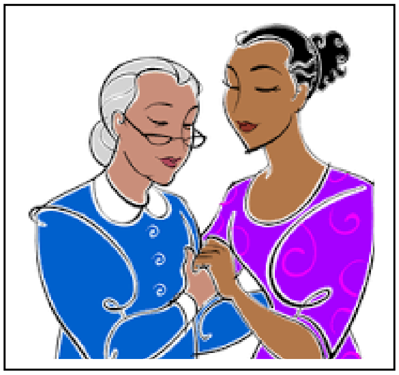

- Establish a daily routine.
- Keep familiar objects around.
- Change the surroundings as little as possible.
- When the surroundings must change, try to prepare your loved one.
- Do not argue or disagree.
- Check out your loved one’s suspicions. They may be based on fact.
- Use touch to reassure your loved one -- if they are open to being touched.
- Do not take being accused personally. Paranoia is part of the disease.

© 2023 Khan BA et al. *JAMA Network Open*.

Help with Hallucinations

Hallucination:

- Misunderstanding the surroundings,
- Causing a person to hear, see, or smell things that are not here
- A hallucination is not based on reality.

Delusion:

- A false belief that is not based on the person's surroundings.
- The belief usually is not consistent with what the person knows as fact and has experienced.

- Make sure there is adequate lighting.
- Do not argue.
- Some medications can cause hallucinations.
- Try distracting your loved one with other activities.
- Get a hearing and vision test performed.
- Get a medical evaluation.
- A psychiatric evaluation may be needed.

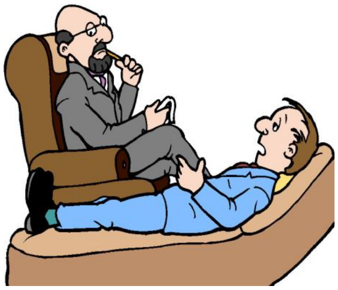

© 2023 Khan BA et al. *JAMA Network Open*.

Help with Inappropriate Sexual Behavior

- Is the behavior simply an expression?
- Is the room temperature comfortable?
- Is clothing comfortable?
- Need to go to the bathroom?
- Are there specific times when this behavior happens? Bath time, bed time?
- Keep routine, surroundings and caregiver consistent.
- Try creating a distraction with food, drink, a walk, or other activity.
- Ignoring the remark or behavior may work.
- Avoid scolding or trying to reason.
- Answer in a matter-of-fact tone.
- Give a gentle touch or appropriate affection such as a hug or holding hands.
- Encourage the family to show affection. This may be what your loved one wants.

© 2023 Khan BA et al. *JAMA Network Open*.

Help with Delirium in the Hospital

Remember to:

- A person with delirium cannot think clearly, has trouble paying attention and is not aware of what is going on around him/her.
- Sometimes your loved one may even see or hear things that are not really there.
- Delirium usually clears up after a few days to weeks.
- Persons with dementia are at higher risk for developing delirium as a result of medications, medical illness and hospitalization for medical procedures.
- If your loved on is going to the hospital, the best thing you can do to help is to work together with your loved one's doctors and nurses to prevent delirium.

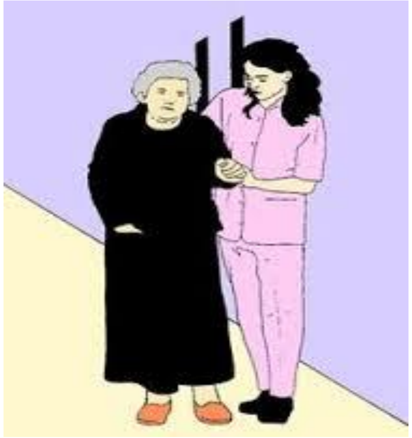

© 2023 Khan BA et al. *JAMA Network Open*.

More Help with Delirium in the Hospital

If your loved one is going to the hospital what can you do to help prevent delirium?

- Provide information regarding any time your loved one has been confused because of illness medications in the past.

- Bring personal items to the hospital to help your loved one feel more at ease and keep them oriented.

- If your loved one wears glasses or hearing aids, bring them to the hospital so that he/she can use them.

- Sit with your loved one as much as possible and help to reassure and calm him/her.

- Offer your loved one a warm drink of milk and a gentle back rub before bedtime.

- Watch your loved one for signs that he/she is in pain or constipated. Notify the doctors and nurses if your loved one is experiencing either of these symptoms or exhibiting any unusual behavior.

© 2023 Khan BA et al. *JAMA Network Open*.

Help with Delirium at Home

If your loved one develops delirium in the hospital, special care will be needed when he/she returns home.

Your loved one needs at least 4-6 hours of uninterrupted sleep at night. If your loved one is having trouble sleeping, try the following:

- Decreases noise at night and provide a warm drink of milk before bedtime.
- Offer your loved one a back rub for 10-15 minutes before bedtime.
- Encourage your loved one to stay awake during the day and discourage daytime napping.
- Keep a clock and calendar inside your loved one's bedroom.
- If your loved one wears glasses or hearing aids, make sure he/she has them available to use.
- Keep a light on in your loved one's bedroom from sunrise to sunshine.

© 2023 Khan BA et al. *JAMA Network Open*.

More Help with Delirium at Home

When your loved one comes home from the hospital :

- Make sure your loved one is taking the right medications in the right dose at the right time. Medications have been started or stopped when your loved was in the hospital. If you have questions, contact the ABC medical Home nurse practitioner immediately.

- Watch for signs that your loved one is in pain or constipated.

- Notify the m-CCRP nurse if your loved one is experiencing either of these symptoms or exhibiting any usual behavior.

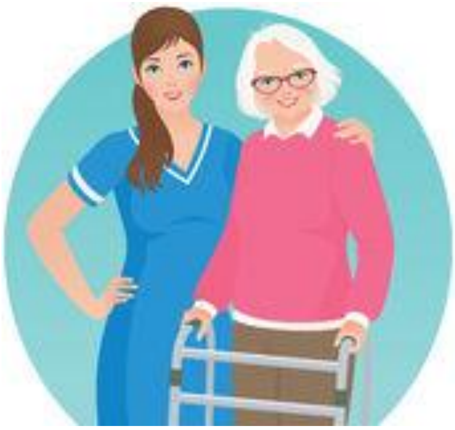

© 2023 Khan BA et al. *JAMA Network Open*.

Taking Care Of Yourself

Remember to :

- Get plenty of rest.
- Drink 8 glasses of water a day and eat right.
- Take a walk everyday with your loved ones.
- Make time for yourself! Do what you enjoy doing.
- Compliance to medications is key.
- Never miss follow up clinics.

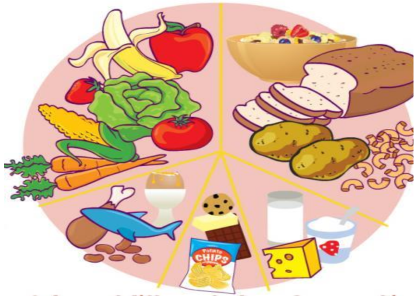

© 2023 Khan BA et al. *JAMA Network Open*.

Guidelines for Coping

- Communication is essential.
- Ask for help - this is a sign of strength. not weakness.
- Tell your family and friends how you feel, don't worry about things to be taken personally.
- Tell your family and friends about wills and power of attorney.

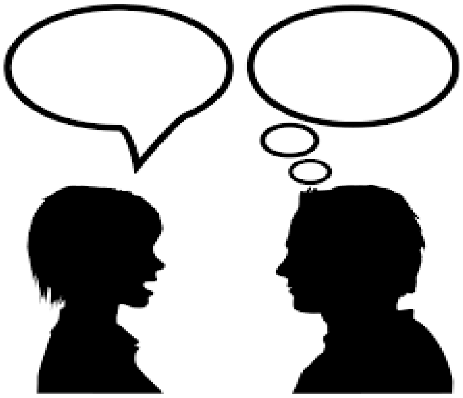

© 2023 Khan BA et al. *JAMA Network Open*.

Looking on the Bright Side

Important patient responses are those of:

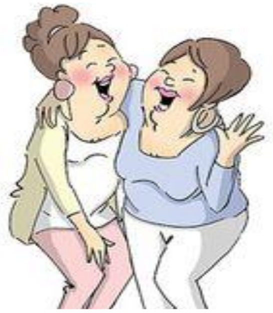

- Appreciative
- Warmth
- Closeness
- Understanding
- Happiness

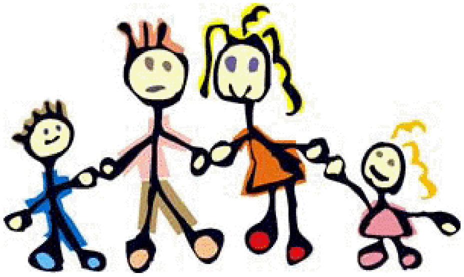

Many family members feel good about being cared by a loved one.

Don't forget to look on the bright side.

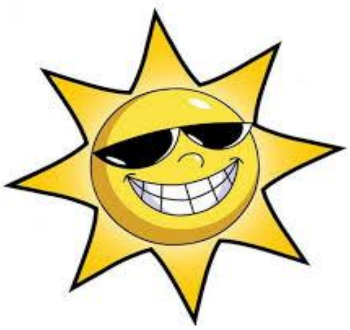

© 2023 Khan BA et al. *JAMA Network Open*.

Communicating with your care giver

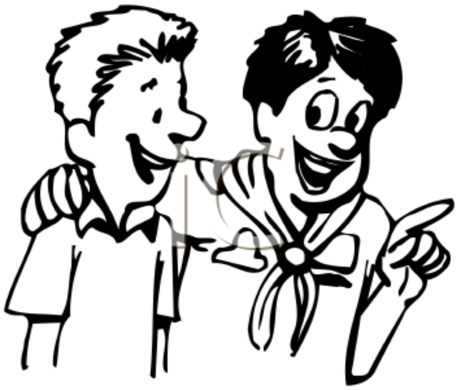

- Identify yourself.
- Speak slowly, clearly and softly.
- Use positive facial expressions.
- Converse in a social and non threatening manner.
- Use simple sentences.
- Allow time for the caregiver to respond.
- If there is no response, repeat exactly what you said. -"I want food."

© 2023 Khan BA et al. *JAMA Network Open*.

Suggestions for communication

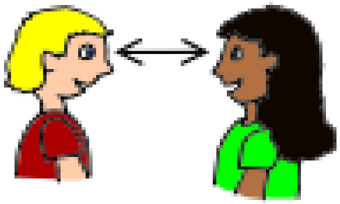

- Maintain eye contact.
- Do not take negative things they say personally.
- Watch for signs of exhaustion, agitation or withdrawal, for example look away or frowning.
- Always treat your care provider with dignity and respect.

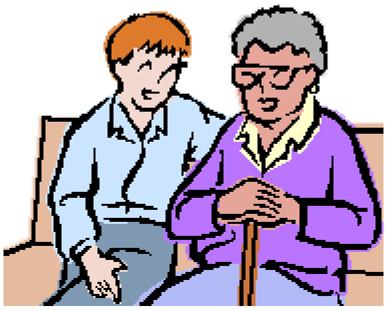

© 2023 Khan BA et al. *JAMA Network Open*.

Addictions

Social addictions are harmful to health. These are proven to be responsible for various cancers and lung diseases. Addictions can have various forms:

- Tobacco smoking
- Illicit drugs including marijuana, cocaine to name a few.
- Alcohol

Discuss quitting with the health care provider. Inquire about alternatives, social groups and therapies.

WE CAN HELP YOU QUIT

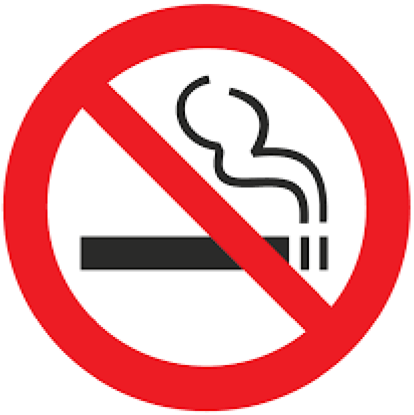

© 2023 Khan BA et al. *JAMA Network Open*.

Help with Exercise

Being active may lead to good sleep and decreased agitation, stress, or moodiness for both you and your loved one. Be sure to get a doctor's approval before you start an exercise plan.

TO INCREASE PHYSICAL ACTIVITY, YOU MAY:

- Turn on your favorite music. Dance by yourself or with a partner for at least 10 minutes or longer at a time.
- Try to follow the routine on a free television exercise program "SIT and BE FIT" on your local PBS station.
- Go for a walk in your neighborhood with your loved one.
- Ride a stationary exercise bike.
- Attend a local exercise classes designed for seniors.
- Try to do stretching, balance and strength building exercises. The National Institute of Aging (NIA) offers a free exercise book and videos, which can be ordered.

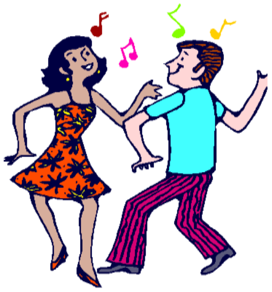

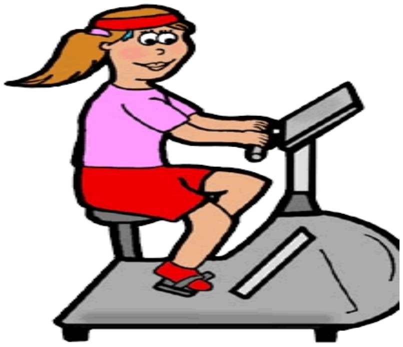

© 2023 Khan BA et al. *JAMA Network Open*.

Activities to Do

- Pleasant current events
- Exercise
- Music and movement
- One-to-one attention
- Reminiscence
- Creative activities
- Food
- Helping around the house

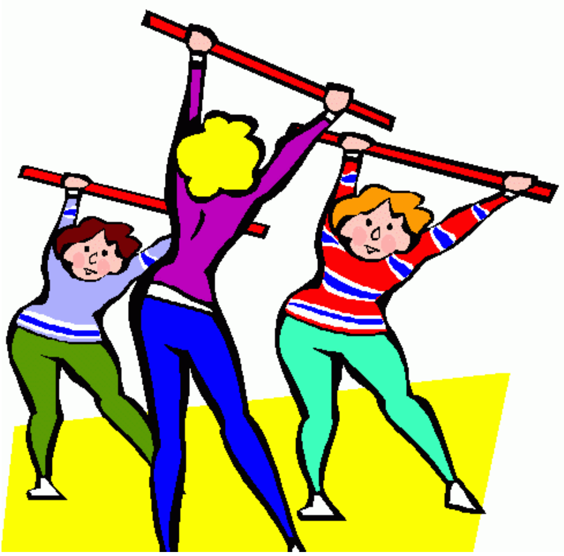

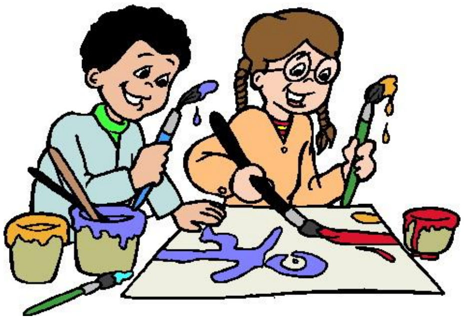

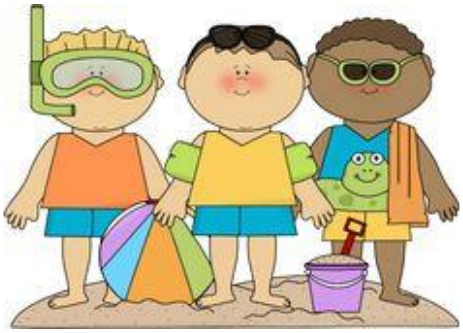

© 2023 Khan BA et al. *JAMA Network Open*.

Activities to Avoid

- Watching the TV or listening to radio all day.
- Noisy, confusing programs or entertainment.
- Long programs.
- Activities that drastically change your routine.
- Doing the same activity every hour.
- Too much activity.

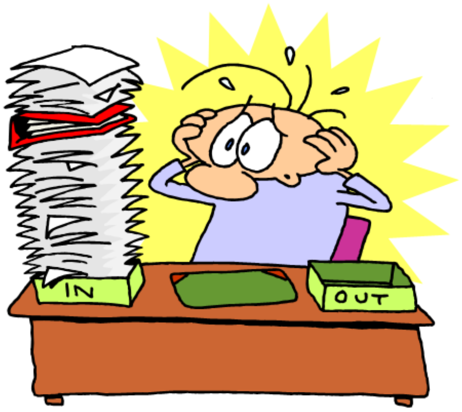

© 2023 Khan BA et al. *JAMA Network Open*.

Getting Dressed

- Do not lock room or bathroom doors while changing.
- Never hesitate to ask for assistance.
- Be flexible and ready to try a new way.
- Avoid clothes that go over the head.
- Use clothes that fasten in front.
- Slip-on shoes with rubber on the bottom are safe and easy.
- Use clothing that is easy and comfortable; Sweat pants are easy and comfortable.
.

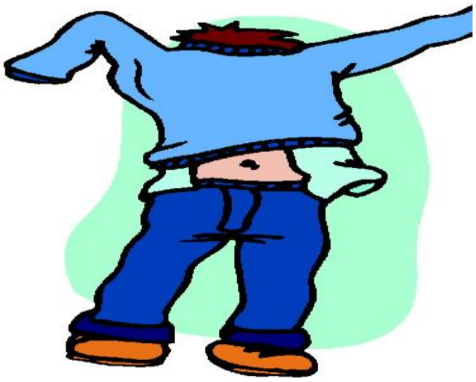

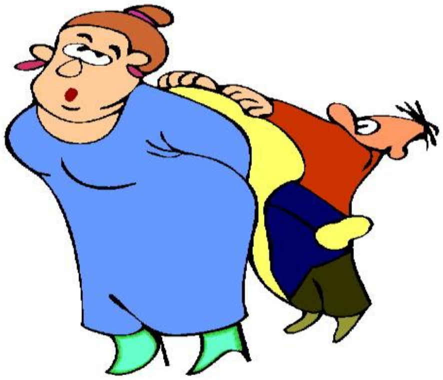

© 2023 Khan BA et al. *JAMA Network Open*.

Bath Time

- Keep bath time the same every day or week.
- Try making use of handle bars or rails. Ask for a chair in the shower if it is easy.
- Do not lock bathroom door
- Give one instruction at a time. For example: “give me the soap”
- Don’t argue, fight, or rush.
- Never let them leave you alone.
- Allow time for relaxation.

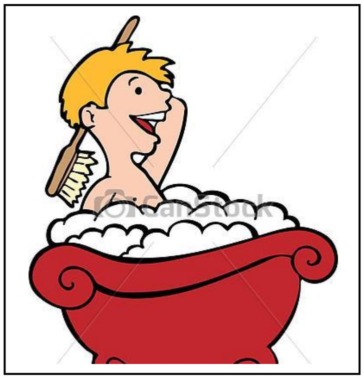

© 2023 Khan BA et al. *JAMA Network Open*.

Toileting

- Do not hesitate to ask for assistance.
- Use the toilet regularly, for example every two hours and not retain.
- Use the toilet before and after meals and before bed.
- Ask for a mobile commode if required.
- Limit caffeine and avoid citrus juices and other bladder irritants
- Avoid skin breakdowns. Remain clean and dry.

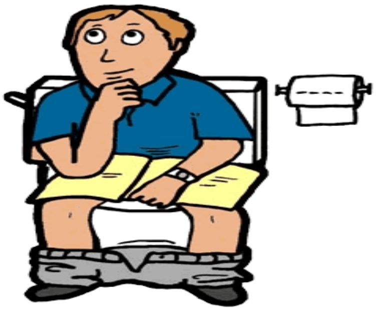

© 2023 Khan BA et al. *JAMA Network Open*.

Meal times

- Eat meals at regular times.
- Ask for soft, relaxing music at mealtimes.
- Use bowls rather than plates.
- Try eating yourselves first.
- Eat slowly and drink lots of water.
- Take enough time to eat and do not rush.

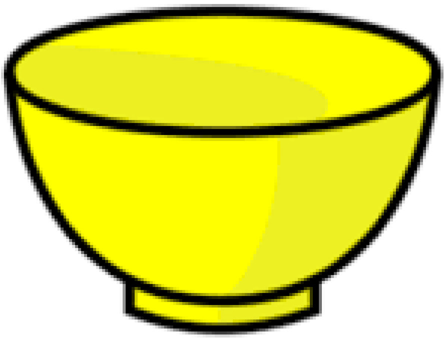

© 2023 Khan BA et al. *JAMA Network Open*.

More Help at Meal times

- Always pay attention to food temperature.
- Avoid Alcohol and alcoholic drinks. They may increase confusion.
- Check for dental and mouth care problems.
- When there's a problem, look for a pattern and inform caregiver immediately.
  -Certain foods causing a problem?
  -Chewing or swallowing a problem?
  -Other people causing agitation?
  -Is something a distraction?

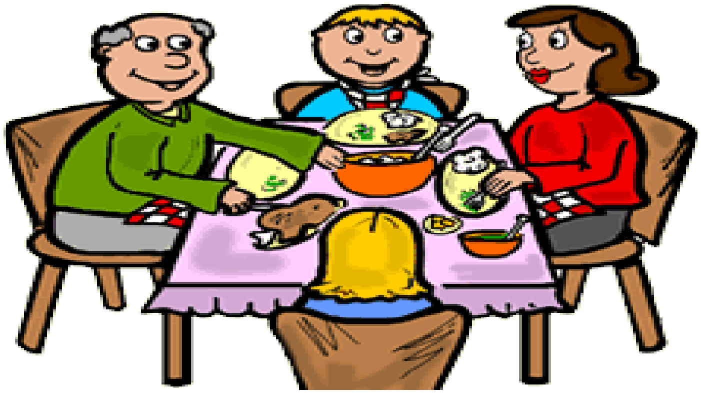

© 2023 Khan BA et al. *JAMA Network Open*.

Dental Care

- Oral health is important. Don't neglect it.
- Ask caregiver for dental appointments
- Use mouthwash that can be swallowed.
- Electric toothbrushes may be confusing.
- Check regularly for mouth sores.
- Check and recheck how dentures are fitting.

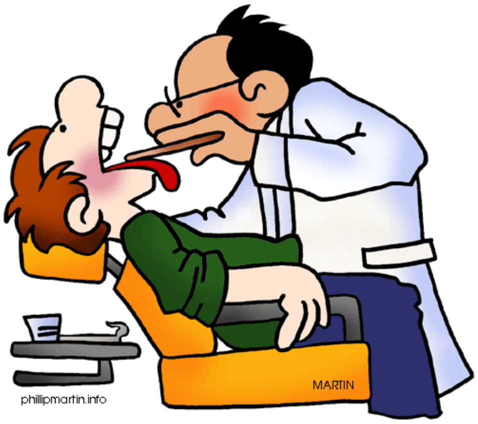

© 2023 Khan BA et al. *JAMA Network Open*.

Help with Balance and Walking

As memory loss progresses, you may become stiff or awkward, may develop stooped or leaning posture or a shuffling walk. This is due to damaged areas of the brain which control muscle movement.

Tell your caregiver about any change in walking, posture, stiffness, repetitive motions, or falling. This may be due to memory loss or there may be a reason that can be treated.

- Walk slowly.
- Make use of installed handrails and grabbers.
- Have your loved one take your arm and hold it tightly.
- Try using a cane or walker for help.

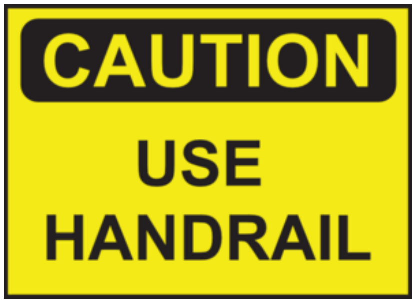

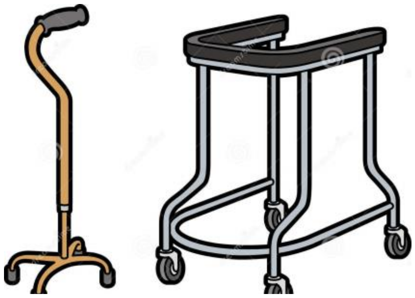

© 2023 Khan BA et al. *JAMA Network Open*.

About Falling and Injuries

When you fall:
1. Remain calm.

2. Let the caregiver help you out.

3. Ask for a doctor if there are injuries symptoms or if there is head injury.
Reasons it's easy to overlook serious injuries:

- Older people are more vulnerable to broken bones from what seems like a minor injury.
- They continue to use a broken arm or leg.
- People with memory loss may not tell they are in pain or may forget to share that they have fell.

Accidents happen when caregivers and loved one's rush.

© 2023 Khan BA et al. *JAMA Network Open*.

Depression

Depression is very common in dementia and a common response to caregiving. Treating depression can significantly improve one's quality of life.

Signs to watch out for:

- Sleeping too much or too little maybe alarming.
- Waking up early in the morning and can't fall back to sleep.
- Eating too little or too much.
- Trouble concentrating.
- Feeling sad all the time and wanting to die.
- Wanting to die.
- Feeling guilty about things in the past.
- Feeling helpless.

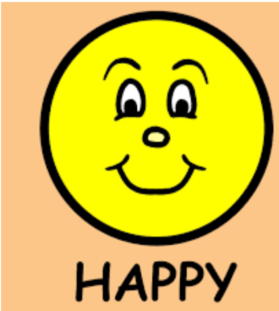

© 2023 Khan BA et al. *JAMA Network Open*.

Help with Sleeping

- Keep proper schedule of sleeping and waking up.

- Being active may lead to good sleep.

- Any change may affect sleep:
  - -Sleeping place
  - -Caregiver change
  - -Different visitors

- Avoid napping during the day.

- Avoid alcohol, caffeine and heavy spicy foods prior to bedtime.

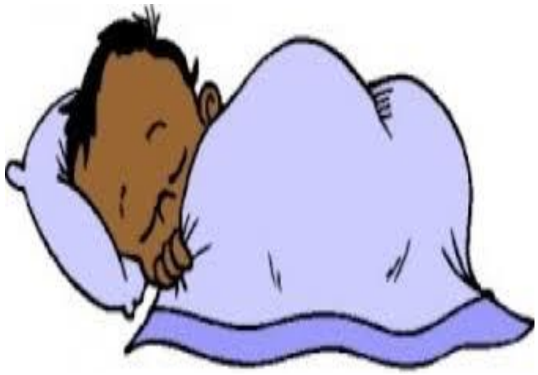

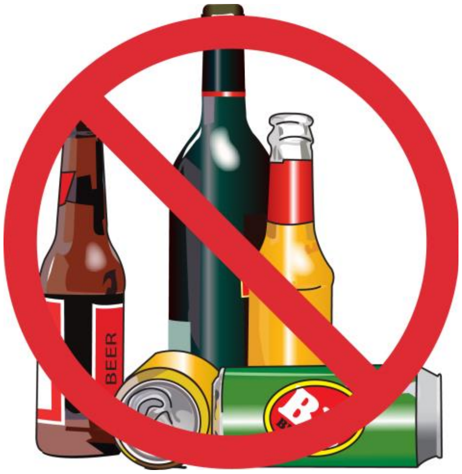

© 2023 Khan BA et al. *JAMA Network Open*.

Help with Wandering

- Make sure your wearing an identification, such as a bracelet with your name, caregiver's phone number, and says “memory impaired.”

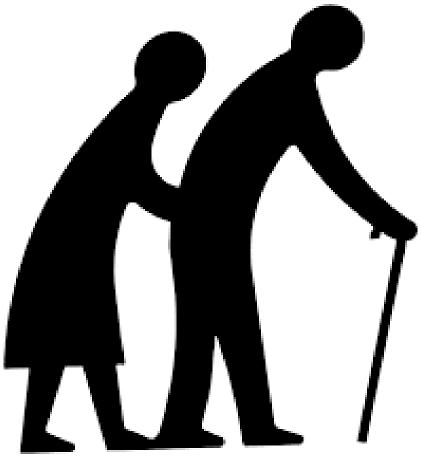

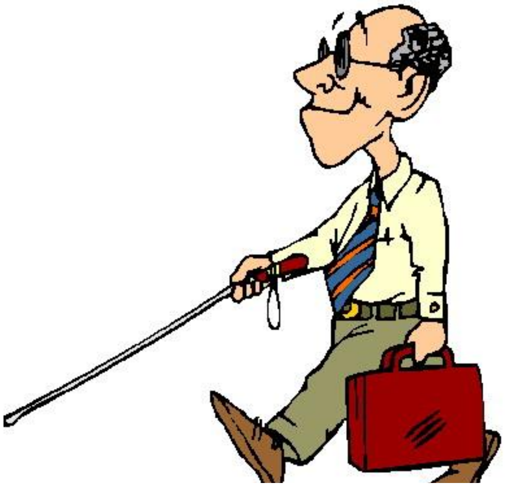

© 2023 Khan BA et al. *JAMA Network Open*.

Catastrophic Reactions and Sun downing

Catastrophic reactions are sudden, unexpected responses that appear to be overreactions to what would normally be viewed as mild stimulation.

Sundown Syndrome: increased agitation and aggressive behavior that occurs late in the day.

**WHY** do you do that?	**THINGS TO TRY** to avoid or smooth out unwanted behavior.
Surroundings are too stimulating, confusing, new.	Simple surroundings.
Change in routine or caregiver.	Maintain a steady routine and schedule.
Caregiver is agitated, frustrated, impatient.	Remain calm.
Too many demands being made on you	Ask to simplify instructions.
Brain damage from the disease causes misunderstanding of surroundings.	Listen to Caregiver.
Side effect of medication.	Ask for a medication review.

© 2023 Khan BA et al. *JAMA Network Open*.

Help with Hallucinations

Hallucination:

-Misunderstanding the surroundings,

-Causing a person to hear, see, or smell things that are not here

-A hallucination is not based on reality.

Delusion:

-A false belief that is not based on the person's surroundings.

-The belief usually is not consistent with what the person knows as fact and has experienced.

- Some medications can cause hallucinations.
- Stay busy.
- Get a hearing and vision test done.
- Get a medical evaluation.
- A psychiatric evaluation may be needed.

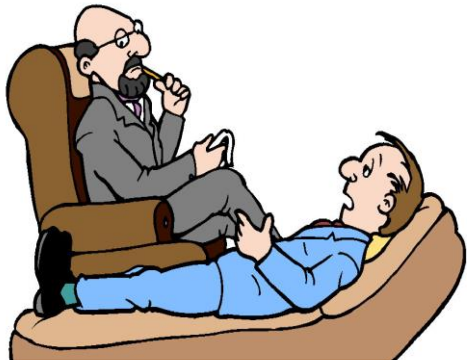

© 2023 Khan BA et al. *JAMA Network Open*.

Help with Inappropriate Sexual Behavior

- Is the behavior simply an expression?
- Is the room temperature comfortable?
- Is clothing comfortable?
- Need to go to the bathroom?
- Are there specific times when this behavior happens? Bath time, bed time?
- Ask for a gentle touch or appropriate affection such as a hug or holding hands.
- Ask the family to show affection. This may be what you want.

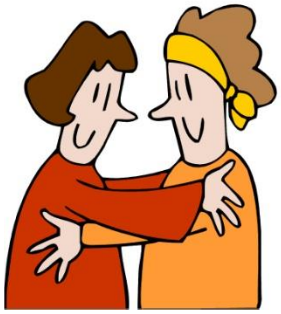

© 2023 Khan BA et al. *JAMA Network Open*.

ICU Survivors Guide: Post-intensive Care Syndrome

Millions of patients are admitted to intensive care units (ICUs) each year, one third of these patients need a machine to help them breathe, such as ventilators. Some of these patients may develop health problems related to their illness, injury, breathing machine or other treatments. Such problems cannot be totally prevented and can continue after the patient leaves the hospital.

Post intensive care syndrome or PICS can happen to a lot of patients when they leave the hospital after being in the ICU. If you are an ICU patient or family member this guide will help you understand PICS. Many people have PICS and help is available. You will have the best chance of getting better if you talk to your primary care doctor about your health problems. You can also get help from the resources at the end of this guide.

What Is PICS?

Post-intensive care syndrome, or PICS, is made up of health problems that continue after critical illness. These problems can involve the patient's body, thoughts, feelings, or mind. PICS may also effect the patient's family. Some signs of PICS are noticeable muscle weakness; problems with thinking and judgment; and other mental health problems.

ICU-acquired weakness

ICU-acquired weakness (ICUAW) is muscle weakness that develops during an ICU stay.

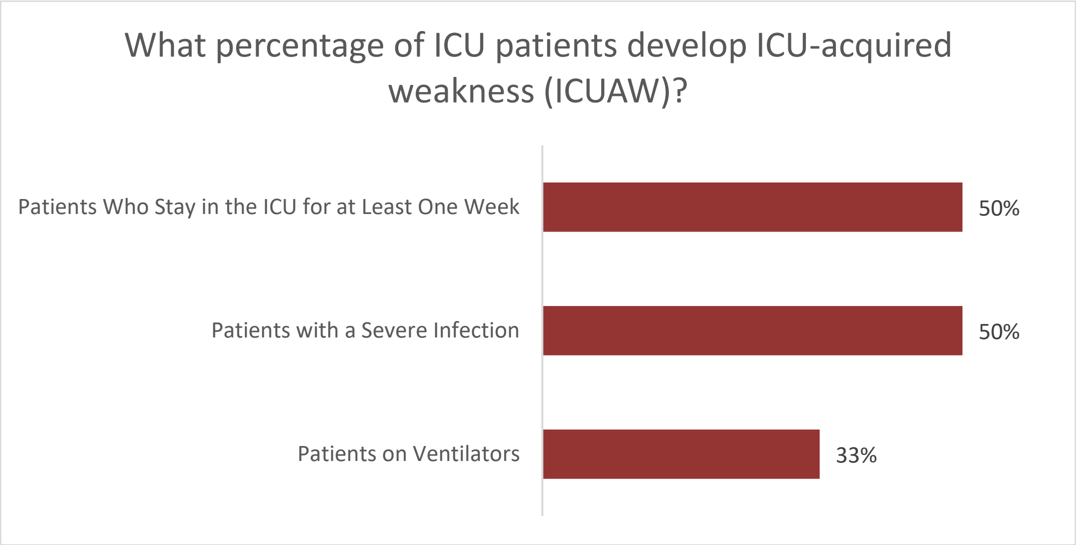

Patients who develop ICUAW may take more than a year to get better. ICUAW makes some activities

© 2023 Khan BA et al. *JAMA Network Open*.

difficult including dressing, feeding, bathing and walking. ICUAW may keep the patient from doing activities in the way he or she used to do them.

Brain dysfunction

Problems with remembering, paying attention, solving problems, and working on complicated tasks are called brain dysfunction. After leaving the ICU, 30% to 80% of patients may have these kinds of problems. Some people get better during the first year after leaving the hospital. Other people may never fully recover.

Patients who have brain dysfunction may have trouble going back to work, balancing a checkbook, or doing things that need organization and focusing.

Other mental health problems

Critically ill patients may feel depressed and anxious.   It is common for patients to feel depressed and/or anxious after leaving the ICU.

How Does PICS Effect Recovery?

Up to 50% of patients may go back to work within the first year. Some patients may not be able to go back to the jobs they had before their illness. Patients may need help with activities after leaving the hospital.

How Does PICS Affect Family?

Critical illness is a family crisis. Feeling worried and confused can cause family members to stop taking care of their own health. Family members may be asked to make difficult decisions. Because of this, 30% of family members might have their own mental health problems. These problems could include depression and anxiety.

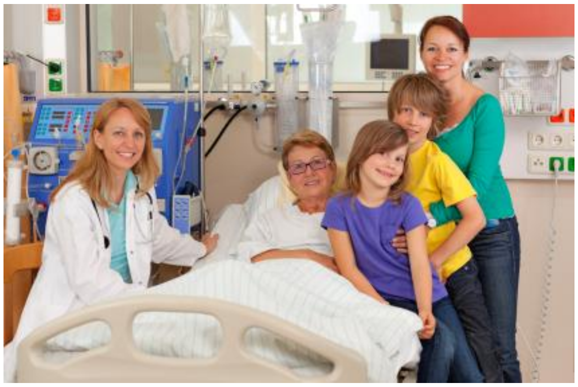

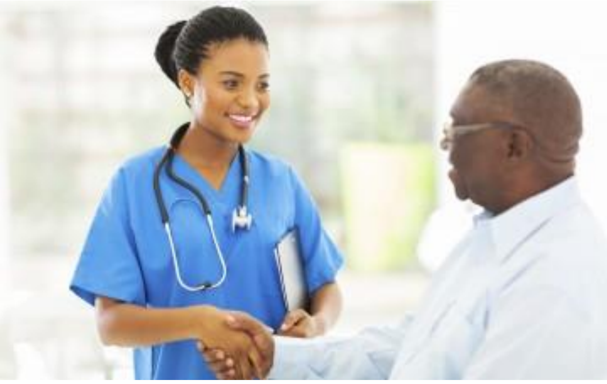

How Can Family Members Lower Their Chances of Developing PICS?

If you are a family member, you need to take care of yourself. This is very important for your health and

© 2023 Khan BA et al. *JAMA Network Open*.

your family member's health. Remember that your health is just as important as your family member's health. If you are healthy, you have the physical and emotional strength to support your family member. Eat a balanced diet, get as much rest as possible, exercise, and ask for support. The hospital has social workers, case managers, and pastoral caregivers who can help.

Take time to understand your family member's illness and treatment options. Having this information will help you make decisions. It will also help you feel confident about your decisions and decrease stress. Ask questions and talk with the care team. Keep a journal. Review the journal with a member of the care team whom you trust. They can help you make sense of what has happened and how you are responding to it. These steps can help you and the care team learn your family member's preferences. That will help the care team to respect your family member's wishes and values as much as possible.

After Leaving the ICU

If you are an ICU patient, the care team may diagnose you with PICS. They may also tell you if you are at risk for developing PICS. They may refer you to other caregivers for support services after you leave the ICU. If not, these are possible warning signs: muscle weakness or problems with balance, problems with thinking and memory, severe anxiety and depression

If you notice any of these problems, please ask your **primary care doctor.**

Important phone numbers:

If you are having problems with your physical activity, please contact the Physical Therapy department.

Eskenazi Health: 317-634-0600, University Hospital: 317-274-1661, Methodist Hospital: 317-962-2000

If you are having any problems with your mood, please contact Midtown services for mental health at 317-554-2704.

If you are having any problems with your brain function such as memory or attention, please contact the Neuroscience center at 317-948-5450.

If you are having problems with your medications, please contact your primary care provider.

If you are having problems with your quality of life, please contact Social Work Department.

Eskenazi Health: 317-880-8687, University Hospital: 317-944-8265, Methodist Hospital: 317-612-2754

Helpful Websites

ICU

These websites have all kinds of information about the critical care setting, the caregivers involved, and what options you have:

- MyICUCare Website, created by the Society of Critical Care Medicine

© 2023 Khan BA et al. *JAMA Network Open*.

- ICU Steps Website, created by a charitable organization

Acute Respiratory Failure

ARDS Network Website: This website, created by the National Heart, Lung and Blood Institute, helps you understand acute respiratory distress syndrome, a problem with the lungs that leads to low oxygen levels in the blood. Acute respiratory distress syndrome is a common reason why patients are in the ICU.

[*]ICU Survivors Guide adopted and modified from Society of Critical Care Medicine page created By: Judy E. Davidson, RN, CNS, DNP, FCCM; Ramona O. Hopkins, PhD; Deborah Louis, RN, MSN; Theodore J. Iwashyna, MD, PhD

© 2023 Khan BA et al. *JAMA Network Open*.

eReferences

1. Knaus WA, Draper EA, Wagner DP, Zimmerman JE. APACHE II: a severity of disease classification system. *Crit Care Med*. Oct 1985;13(10):818-29.
2. Charlson ME, Pompei P, Ales KL, MacKenzie CR. A new method of classifying prognostic comorbidity in longitudinal studies: development and validation. *J Chronic Dis*. 1987;40(5):373-83. doi:10.1016/0021-9681(87)90171-8
3. Jorm AF, Jacomb PA. The Informant Questionnaire on Cognitive Decline in the Elderly (IQCODE): socio-demographic correlates, reliability, validity and some norms. *Psychol Med*. Nov 1989;19(4):1015-22. doi:10.1017/s0033291700005742
4. Katz S, Ford AB, Moskowitz RW, Jackson BA, Jaffe MW. Studies of Illness in the Aged. The Index of Adl: A Standardized Measure of Biological and Psychosocial Function. *JAMA*. Sep 21 1963;185:914-9. doi:10.1001/jama.1963.03060120024016
5. Lawton MP, Brody EM. Assessment of older people: self-maintaining and instrumental activities of daily living. *Gerontologist*. Autumn 1969;9(3):179-86.
6. Folstein MF, Folstein SE, McHugh PR. "Mini-mental state". A practical method for grading the cognitive state of patients for the clinician. *J Psychiatr Res*. Nov 1975;12(3):189-98. doi:10.1016/0022-3956(75)90026-6
7. Podsiadlo D, Richardson S. The timed "Up & Go": a test of basic functional mobility for frail elderly persons. *J Am Geriatr Soc*. Feb 1991;39(2):142-8. doi:10.1111/j.1532-5415.1991.tb01616.x
8. Zigmond AS, Snaith RP. The hospital anxiety and depression scale. *Acta Psychiatr Scand*. Jun 1983;67(6):361-70. doi:10.1111/j.1600-0447.1983.tb09716.x
9. Monahan PO, Alder CA, Khan BA, Stump T, Boustani MA. The Healthy Aging Brain Care (HABC) Monitor: validation of the Patient Self-Report Version of the clinical tool designed to measure and monitor cognitive, functional, and psychological health. *Clin Interv Aging*. 2014;9:2123-32. doi:10.2147/CIA.S64140
10. Wang S, Allen D, Perkins A, et al. Validation of a New Clinical Tool for Post-Intensive Care Syndrome. *Am J Crit Care*. Jan 2019;28(1):10-18. doi:10.4037/ajcc2019639
11. Wang S, Jawed Y, Perkins A, et al. Healthy Aging Brain Care Monitor, Caregiver Version: Screening for Post-Intensive Care Syndrome. *Am J Crit Care*. Mar 1 2022;31(2):137-144. doi:10.4037/ajcc2022451
12. Krebs EE, Lorenz KA, Bair MJ, et al. Development and initial validation of the PEG, a three-item scale assessing pain intensity and interference. *J Gen Intern Med*. Jun 2009;24(6):733-8. doi:10.1007/s11606-009-0981-1
13. Yu L, Buysse DJ, Germain A, et al. Development of short forms from the PROMIS sleep disturbance and Sleep-Related Impairment item banks. *Behav Sleep Med*. Dec 28 2011;10(1):6-24. doi:10.1080/15402002.2012.636266

© 2023 Khan BA et al. *JAMA Network Open*.

eBox. m-CCRP Care Coordinator Services

m-CCRP Care Coordinator Services

- Self-Management Skills Enhancement
- Support groups
- Continuous access to care coordinator via telephone support
- Medications Reconciliation
- Cognitive Protocol (Paper and computer based cognitive training focused on memory, attention, executive function)
- Physical protocol (Multimodal exercises focused on seated aerobic and progressive resistance training)
- Psychological protocol (Problem solving therapy, cognitive behavioral therapy, SSRIs per PCP)
- Monitoring caregiver’s emotional health
- Managing transitional care
- Managing acute care needs
- Root-Cause analysis of re-hospitalization or emergency room visits

© 2023 Khan BA et al. *JAMA Network Open*.

eTable 1. m-CCRP Intervention Effects on SF-36 Subscales

Short Form-36 Dimensions and Assessment Timepoints	Control		m-CCRP Intervention		Estimated Difference in Change from Baseline (95% CI)	P-value
	N	Mean (SD)	N	Mean (SD)		
**Physical Functioning[a]**						*0.770*
Baseline	223	29.76 (27.25)	224	31.49 (27.98)		
3 Months	164	43.60 (29.79)	164	46.42 (29.25)	2.34 (-3.78, 8.47)	0.453
6 Months	159	48.02 (30.01)	151	46.86 (30.24)	0.26 (-6.51, 7.03)	0.940
12 Months	146	49.87 (29.83)	153	51.26 (31.25)	1.40 (-5.60, 8.40)	0.694
**Role-Physical**						*0.744*
Baseline	223	17.49 (30.24)	223	18.39 (31.99)		
3 Months	165	30.30 (34.35)	164	29.57 (34.39)	-0.42 (-8.78, 7.93)	0.921
6 Months	159	42.14 (41.16)	152	44.41 (40.09)	4.05 (-5.81, 13.91)	0.420
12 Months	147	43.20 (38.66)	153	46.46 (41.28)	2.55 (-6.91, 12.01)	0.597
**Pain**						*0.346*
Baseline	223	40.86 (31.71)	224	39.65 (31.78)		
3 Months	165	53.94 (31.04)	164	57.73 (30.07)	6.06 (-1.27, 13.39)	0.105
6 Months	159	59.53 (30.78)	152	58.98 (28.62)	3.09 (-4.48, 10.66)	0.423
12 Months	147	60.19 (29.13)	153	59.44 (30.44)	1.88 (-5.70, 9.47)	0.626
**General Health**						*0.923*
Baseline	222	44.21 (20.43)	223	44.64 (20.24)		
3 Months	165	48.48 (22.60)	164	48.11 (22.07)	-0.24 (-4.36, 3.88)	0.909
6 Months	159	47.74 (22.91)	152	46.02 (22.15)	-0.09 (-4.46, 4.29)	0.969
12 Months	147	50.23 (22.01)	153	49.93 (23.72)	0.94 (-3.45, 5.32)	0.675
**Vitality**						*0.970*
Baseline	223	45.29 (32.82)	224	48.49 (31.36)		
3 Months	165	63.79 (31.16)	164	67.30 (28.32)	-0.94 (-6.44, 4.56)	0.738
6 Months	159	68.79 (30.74)	152	68.91 (30.38)	-0.15 (-5.99, 5.70)	0.961
12 Months	147	70.07 (27.74)	153	67.73 (31.04)	0.14 (-5.23, 5.52)	0.958

© 2023 Khan BA et al. *JAMA Network Open*.

**Social Functioning**						*0.625*
Baseline	222	34.25 (23.57)	224	36.25 (23.20)		
3 Months	165	44.93 (25.55)	164	45.71 (23.17)	1.02 (-6.70, 8.75)	0.795
6 Months	159	46.19 (24.51)	152	46.55 (24.66)	-0.60 (-8.80, 7.60)	0.886
12 Months	147	46.97 (22.40)	153	48.46 (22.46)	-3.35 (-11.80, 5.10)	0.436
**Role Emotional**						*0.213*
Baseline	223	49.78 (41.96)	224	55.36 (41.29)		
3 Months	165	64.85 (41.03)	164	66.26 (40.95)	-2.62 (-13.09, 7.85)	0.623
6 Months	159	69.81 (40.70)	152	68.42 (40.56)	-4.31 (-15.02, 6.41)	0.430
12 Months	147	72.34 (37.29)	153	66.67 (40.47)	-10.03 (-20.28, 0.23)	0.055
**Emotional Well-Being**						*0.831*
Baseline	222	64.30 (22.81)	224	66.96 (23.45)		
3 Months	165	72.15 (20.90)	164	73.07 (21.75)	-1.07 (-5.57, 3.42)	0.639
6 Months	159	73.07 (19.52)	152	71.50 (23.89)	-2.29 (-7.44, 2.85)	0.382
12 Months	147	74.42 (20.16)	153	73.41 (22.29)	-2.12 (-7.45, 3.21)	0.435

[a]Physical functioning, limitations in physical activities because of health problems; role physical, limitations in usual role activities because of physical health problems; bodily pain, the perceived amount of pain and interference with normal activities; general health, general health as perceived by self; vitality, feelings of energy vs. tiredness; social functioning, limitations in social activities because of physical or behavioral/mood problems; role emotional, limitations in usual role activities because of emotional problems; mental health, feelings of anxiety or depression. Higher scores represent better health status.

© 2023 Khan BA et al. *JAMA Network Open*.

eTable 2. m-CCRP Intervention Effects on Repeatable Battery for the Assessment of Neuropsychological Status (RBANS) Subscales

Outcomes	Control		m-CCRP Intervention		Estimated Difference in Change from Baseline (95% CI)	P-value
	N	Mean (SD)	N	Mean (SD)		
**List Learning Z-Score[a]**						*0.981*
Baseline	167	0.00 (1.06)	177	0.00 (0.95)		
3 Months	149	0.24 (1.00)	142	0.12 (1.01)	-0.04 (-0.24, 0.16)	0.691
6 Months	142	0.46 (0.94)	135	0.38 (0.96)	-0.04 (-0.25, 0.17)	0.730
12 Months	133	0.17 (0.99)	129	0.14 (1.04)	-0.03 (-0.26, 0.20)	0.808
**Story Memory Z-Score**						*0.538*
Baseline	165	0.01 (0.96)	176	0.00 (1.04)		
3 Months	147	-0.38 (0.92)	141	-0.50 (0.96)	-0.05 (-0.27, 0.16)	0.631
6 Months	142	0.31 (1.07)	135	0.19 (0.97)	-0.02 (-0.24, 0.20)	0.884
12 Months	133	0.28 (0.95)	129	0.29 (0.99)	0.10 (-0.13, 0.33)	0.396
**Semantic Fluency Z-Score**						*0.741*
Baseline	162	-0.03 (1.00)	175	0.03 (1.00)		
3 Months	146	-0.04 (0.88)	141	0.03 (0.84)	0.06 (-0.17, 0.28)	0.634
6 Months	142	0.22 (0.88)	135	0.25 (0.93)	0.00 (-0.25, 0.24)	0.975
12 Months	133	0.20 (1.09)	129	0.38 (1.03)	0.12 (-0.15, 0.38)	0.386
**Digit Span Z-score**						*0.505*
Baseline	163	-0.03 (0.97)	176	0.02 (1.03)		
3 Months	146	0.21 (0.91)	141	0.09 (0.97)	-0.11 (-0.31, 0.10)	0.302
6 Months	142	0.28 (1.06)	135	0.20 (1.09)	-0.08 (-0.31, 0.15)	0.504
12 Months	133	0.11 (0.99)	128	0.15 (0.97)	0.03 (-0.18, 0.24)	0.792
**List Recall Z-score**						*0.558*
Baseline	162	-0.01 (0.98)	176	0.01 (1.02)		
3 Months	146	0.40 (0.93)	140	0.20 (0.94)	-0.14 (-0.36, 0.07)	0.181
6 Months	142	0.49 (0.93)	134	0.37 (0.98)	-0.14 (-0.36, 0.08)	0.216

© 2023 Khan BA et al. *JAMA Network Open*.

12 Months	133	0.28 (0.96)	129	0.19 (0.99)	-0.11 (-0.35, 0.12)	0.342
**List Recognition Z-score**						*0.763*
Baseline	162	-0.04 (1.05)	175	0.04 (0.97)		
3 Months	146	0.26 (0.74)	141	0.18 (1.00)	-0.10 (-0.32, 0.13)	0.406
6 Months	142	0.23 (0.80)	135	0.26 (0.93)	0.00 (-0.22, 0.22)	0.994
12 Months	133	0.28 (0.74)	129	0.30 (0.78)	-0.04 (-0.26, 0.17)	0.681
**Story Recall Z-Score**						*0.928*
Baseline	162	0.02 (0.98)	176	-0.02 (1.02)		
3 Months	146	-0.18 (0.81)	141	-0.28 (0.92)	-0.01 (-0.22, 0.20)	0.954
6 Months	142	0.40 (0.97)	133	0.34 (0.95)	0.03 (-0.18, 0.25)	0.749
12 Months	133	0.50 (0.96)	129	0.45 (1.01)	0.06 (-0.18, 0.29)	0.627

[a]For each individual subscale, the z-score is created by taking the (score-baseline mean)/standard deviation. The z-scores for the subscales have a mean of 0 and standard deviation of 1. The overall z-score is the mean of the 7-item z-scores.

© 2023 Khan BA et al. *JAMA Network Open*.

eTable 3A. Time to Emergency Department Visit, Re-Hospitalization, and Death Between m-CCRP Intervention and Control groups.

Outcome	Hazards Ratio (95% CI)	P-value
**Time to Emergency Department Visit, Readmission, Death[a]**		
Emergency Department Visit	1.16 (0.94, 1.43)	0.180
Inpatient Readmission	1.06 (0.89, 1.26)	0.508
Death	0.68 (0.40, 1.13)	0.137

[a]The models are adjusted for stratification variables (site, noninvasive ventilation) as well as age, Acute Physiology and Chronic Health Evaluation-II score, Charlson Comorbidity Index, and Intensive Care Unit length of stay.

eTable 3B. Time to Inpatient Hospitalization by Discharge Location Between m-CCRP Intervention and Control groups.

Time to Inpatient Hospitalization by Discharge Location[b]		
**Discharge Location**	**HR (95% CI)**	**P-value**
Home (reference)		
Inpatient Rehabilitation	1.13 (0.79, 1.60)	0.505
Skilled Nursing Facility	0.78 (0.26, 2.36)	0.661
Long term Acute Care	1.40 (0.73, 2.67)	0.310
Acute Rehab Facility	1.45 (0.98, 2.13)	0.061
Other	0.69 (0.08, 5.77)	0.735

[b]The models are adjusted for stratification variables (site, noninvasive ventilation) as well as age, Acute Physiology and Chronic Health Evaluation-II score, Charlson Comorbidity Index, and Intensive Care Unit length of stay, and randomization.

© 2023 Khan BA et al. *JAMA Network Open*.

eTable 3C. Comparison of Emergency Department Visits and Re-hospitalizations Between Patients Discharged to Home Versus Facility in Both Control and m-CCRP Groups

Control							
	**Home (n=135)**	**Inpatient Rehab (n=50)**	**SNF[a] (n=8)**	**LTAC[b] (n=12)**	**Acute Rehab (n=23)**	**Other (n=5)**	**P-value**
Re-hospitalizations n (%)	50 (37.0)	21 (42.0)	3 (37.5)	6 (50.0)	15 (65.2)	0 (0.0)	0.065
Emergency Department Visits n (%)	45 (33.3)	19 (38.0)	4 (50.0)	3 (25.0)	10 (43.5)	0 (0.0)	0.403
**m-CCRP**							
	**Home (n=141)**	**Inpatient Rehab (n=52)**	**SNF[a] (n=6)**	**LTAC[b] (n=11)**	**Acute Rehab (n=22)**	**Other (n=1)**	**P-value**
Re-hospitalizations n (%)	69 (48.8)	26 (50.0)	2 (33.3)	5 (45.5)	14 (63.6)	1 (100.0)	0.630
Emergency Department Visits n (%)	58 (41.1)	19 (36.5)	2 (33.3)	3 (27.3)	11 (50.0)	0 (0.0)	0.735

[a]SNF=Skilled Nursing Facility
[b]LTAC= Long-term Acute Care

© 2023 Khan BA et al. *JAMA Network Open*.

eTable 4A. Emergency Department Diagnoses and Reasons at the Encounter Level

Diagnoses	Control (n=159)	m-CCRP (n=187)
**Emergency Department Visit Diagnoses**		
Cardiac	30 (18.9)	28 (15.0)
Falls	23 (14.5)	27 (14.4)
Sepsis	20 (12.6)	13 (7.0)
Respiratory	18 (11.3)	30 (16.0)
Gastrointestinal	13 (8.2)	17 (9.1)
Renal	9 (5.7)	11 (5.9)
Surgical/Trauma	9 (5.7)	14 (7.5)
Central Nervous System/Neurologic	7 (4.4)	12 (6.4)
Hematologic	7 (4.4)	3 (1.6)
Endocrine/Metabolic	4 (2.5)	9 (4.8)
Mental Health	1 (0.6)	3 (1.6)
Musculoskeletal	1 (0.6)	1 (0.5)
Other[a]	17 (10.7)	18 (9.6)
**Emergency Department Visit Reasons**		
Acute Care	80 (50.3)	88 (47.1)
Transitional Care	71 (44.7)	94 (50.3)
Physical	24 (15.1)	27 (14.4)
Cognitive	4 (2.5)	0 (0.0)
Emotional Health	1 (0.6)	4 (2.1)
Medication Issue	1 (0.6)	1 (0.3)

[a]Other includes vascular access issues, malignancy, epistaxis, vision changes, concern for foreign body, abnormal outpatient labs.

© 2023 Khan BA et al. *JAMA Network Open*.

eTable 4B. Hospital Readmission Diagnoses and Reasons at the Encounter Level

Diagnoses	Control (n=255)	m-CCRP (n=260)
**Hospital Readmission Diagnoses**		
Sepsis	58 (22.7)	50 (19.2)
Endocrine/Metabolic	44 (17.3)	53 (20.4)
Respiratory	44 (17.3)	40 (15.4)
Cardiac	28 (11.0)	38 (14.6)
Surgical/Trauma	18 (7.1)	21 (8.1)
Gastrointestinal	17 (6.7)	20 (7.7)
Hematologic	17 (6.7)	9 (3.5)
Central Nervous System/Neurologic	12 (4.7)	12 (4.6)
Falls	10 (3.9)	6 (2.3)
Renal	5 (2.0)	7 (2.7)
Mental Health	1 (0.4)	5 (1.9)
Other[a]	6 (2.4)	6 (2.3)
**Hospital Readmission Reasons**		
Acute Care	208 (81.6)	203 (78.1)
Transitional Care	90 (35.3)	96 (36.9)
Cognitive	12 (4.7)	12 (4.6)
Physical	11 (4.3)	4 (1.5)
Emotional Health	2 (0.8)	8 (3.1)
Medication Issue	0 (0.0)	1 (0.4)

[a]Other includes vascular access issues, malignancy, epistaxis, vision changes, concern for foreign body, abnormal outpatient labs.

© 2023 Khan BA et al. *JAMA Network Open*.

eTable 5. Initiation of m-CCRP Protocols by Discharge Status

	m-CCRP Intervention Protocols (n=233)						
**Protocols Initiated, n (%)**	**Overall (n=233)**	**Home (n=141)**	**Inpatient Rehab (n=52)**	**SNF[a] (n=6)**	**LTAC[b] (n=11)**	**Acute Rehab (n=22)**	**Other (n=1)**
Sleep Disturbances	151 (64.8)	92 (65.3)	31 (59.6)	4 (66.7)	7 (63.6)	17 (77.3)	0 (0.0)
Exercise	149 (64.0)	89 (63.1)	31 (59.6)	5 (83.3)	8 (72.7)	16 (72.7)	0 (0.0)
Physical Health	147 (63.1)	88 (62.4)	31 (59.6)	4 (66.7)	7 (63.6)	17 (77.3)	0 (0.0)
Mobility	139 (59.7)	84 (59.6)	27 (51.9)	4 (66.7)	7 (63.6)	17 (77.3)	0 (0.0)
Pain	123 (52.8)	73 (51.8)	28 (53.9)	3 (50.0)	6 (54.6)	13 (59.1)	0 (0.0)
Depression	103 (44.2)	65 (46.1)	16 (30.8)	3 (50.0)	6 (54.6)	13 (59.1)	0 (0.0)
Anxiety	103 (44.2)	64 (45.4)	17 (32.7)	3 (50.0)	6 (54.6)	13 (59.1)	0 (0.0)
Behavioral Care	98 (42.1)	61 (43.3)	19 (36.5)	2 (33.3)	5 (45.5)	11 (50.0)	0 (0.0)
Stress	91 (39.1)	57 (40.4)	14 (26.9)	2 (33.3)	6 (54.6)	12 (54.6)	0 (0.0)
Cognition	67 (28.8)	38 (27.0)	12 (23.1)	5 (83.3)	5 (45.5)	7 (31.8)	0 (0.0)
Personal Care	29 (12.4)	15 (10.6)	9 (17.3)	2 (33.3)	3 (27.3)	0 (0.0)	0 (0.0)
Medication Adherence	13 (5.6)	8 (5.7)	4 (7.7)	0 (0.0)	1 (9.1)	0 (0.0)	0 (0.0)
Acute Care Reduction/Delirium	11 (4.7)	4 (2.8)	4 (7.7)	0 (0.0)	3 (27.3)	0 (0.0)	0 (0.0)
Legal and Financial	4 (1.7)	3 (2.1)	0 (0.0)	0 (0.0)	1 (9.1)	0 (0.0)	0 (0.0)
Communication	4 (1.7)	1 (0.7)	1 (1.9)	0 (0.0)	2 (18.2)	0 (0.0)	0 (0.0)

[a]SNF=Skilled nursing facility
[b]LTAC=Long-term acute care hospital.

© 2023 Khan BA et al. *JAMA Network Open*.

eTable 6. Days From Discharge to Implementation of m-CCRP Protocols

Protocols Initiated, n	Median Time to Implementation, median (IQR), days	Within 2 weeks, n (%)	Weeks 3-4, n (%)	Weeks 5-12, n (%)	Week 13+, n (%)
Sleep Disturbances (n = 151)	27 (19, 49)	22 (14.6)	60 (39.7)	46 (30.5)	23 (15.2)
Exercise (n = 149)	27 (19, 53)	21 (14.1)	61 (40.9)	48 (32.2)	19 (12.8)
Physical Health (n = 147)	28 (18, 66)	25 (17.0)	51 (34.7)	42 (28.6)	29 (19.7)
Mobility (n = 139)	28 (18, 59)	22 (15.8)	49 (35.3)	44 (31.7)	24 (17.3)
Cognition (n = 67)	30 (20, 94)	5 (7.5)	27 (40.3)	16 (23.9)	19 (28.4)
Personal Care (n = 29)	31 (20, 85)	1 (3.5)	13 (44.8)	7 (24.1)	8 (27.6)
Pain (n = 123)	33 (21, 85)	13 (10.6)	39 (31.7)	40 (32.5)	31 (25.2)
Anxiety (n = 103)	37 (21, 106)	12 (11.7)	32 (31.1)	27 (26.2)	32 (31.1)
Medication Adherence (n = 13)	44 (26, 61)	0 (0.0)	4 (30.8)	7 (53.8)	2 (15.4)
Depression (n = 103)	45 (21, 118)	11 (10.7)	31 (30.1)	28 (27.2)	33 (32.0)
Stress (n = 91)	48 (19, 124)	14 (15.4)	26 (28.6)	19 (20.9)	32 (35.2)
Communication (n = 4)	57 (35, 84.5)	0 (0.0)	0 (0.0)	3 (75.0)	1 (25.0)
Behavioral Care (n = 98)	57.5 (27, 108)	9 (9.2)	19 (19.4)	28 (28.6)	42 (42.9)
Acute Care Reduction/Delirium (n = 11)	62 (30, 87)	0 (0.0)	2 (18.2)	5 (45.4)	4 (36.4)
Legal and Financial (n = 4)	134.5 (114.5, 259)	0 (0.0)	0 (0.0)	0 (0.0)	4 (100.0)

© 2023 Khan BA et al. *JAMA Network Open*.

eTable 7. Comparison of Outpatient Orders and Medication Orders Between m-CCRP and Control

Orders	m-CCRP (n=233)	Control (n=233)	P-value
**Outpatient Orders and Consults**			
Referral to Physical Therapy	47 (20.2)	50 (21.5)	0.732
Referral to Primary Care and Transitional Care	85 (36.5)	80 (34.3)	0.628
Diabetes, Weight Loss, Dietician	27 (11.6)	11 (4.7)	0.010
Home Health and Home Services including Medical Supplies	14 (6.0)	17 (7.3)	0.577
Other Rehabilitation Service	12 (5.2)	10 (4.3)	0.662
Referral for Occupational Therapy	7 (3.0)	9 (3.9)	0.611
Referral for Pain Diagnosis and Management	6 (2.6)	8 (3.4)	0.587
Referral for Speech Therapy	6 (2.6)	7 (3.0)	0.779
Referral to Mental Health	11 (4.7)	9 (3.9)	0.648
Referral to Social Work and Mental Services	14 (6.0)	4 (1.7)	0.028
Referral to Senior Care	3 (1.3)	1 (0.4)	0.624
Referral to Specialties, Symptom-Focused	78 (33.5)	68 (29.2)	0.318
Sleep	25 (10.7)	18 (7.7)	0.263
**Outpatient Medication Orders**			
Antidepressants	95 (40.8)	87 (37.3)	0.448
Antianxiety/Anxiolytics	66 (28.3)	49 (21.0)	0.068
Pain/Analgesics	165 (70.8)	160 (68.7)	0.614
Sleep	60 (25.8)	68 (29.2)	0.406

© 2023 Khan BA et al. *JAMA Network Open*.

eTable 8. Comparison of Outpatient Care Related to Initiated Protocols Between m-CCRP and Control

Protocol based on Orders[a]	m-CCRP (n=233)	Control (n=233)	P-value
Transitional Care	119 (51.1)	113 (48.5)	0.578
Physical Health	111 (47.6)	102 (43.8)	0.403
Communication/Personal Care	68 (29.2)	56 (24.0)	0.208
Acute Care	63 (27.0)	69 (29.6)	0.607
Exercise	56 (24.0)	60 (25.8)	0.668
Pain	37 (15.9)	30 (12.9)	0.355
Cognition	36 (15.5)	40 (17.2)	0.616
Sleep	26 (11.2)	21 (9.0)	0.442
Legal/Financial	13 (5.6)	4 (1.7)	0.045
Stress	13 (5.6)	4 (1.7)	0.045
Depression or Mental Health	12 (5.2)	10 (4.3)	0.662
Medications	1 (0.4)	1 (0.4)	1.000

[a]Transitional care included orders to primary care, sub-specialty care (medical and surgical), geriatrics care, transitional care services. Physical health and exercise included referrals to physical therapy, occupational therapy, exercise programs, yoga therapy, spa, cardiopulmonary rehabilitation services, weight loss services. Personal Care included weight management services, home health and home medical supplies. Communication included vision, hearing, speech services. Acute care included admissions and emergency department visits. Exercise included rehabilitation services. Pain included pain management services, pain classes, electromyography, orthototics. Cognition included referrals to memory care clinics, neurology, neuropsychology, speech therapy. Sleep included orders for positive pressure devices, sleep consults, sleep studies, and access to device care. Legal and financial included referrals to care management and social work, community-based assistance programs, meals on wheels, financial counseling, safety concerns. Stress included spa therapy orders, and social work. Mental Health included psychology, psychiatry, geriatric psychiatry, psychotherapy. Medications included referral to pharmacists.

© 2023 Khan BA et al. *JAMA Network Open*.

eTable 9. Comparison of Study Outcomes between Study Groups in Participants ≥65 Years of Age

Outcomes	Control		m-CCRP Intervention		Estimated Difference in Change from Baseline (95% CI)	P-value
	N	Mean (SD)	N	Mean (SD)		
**SF-36 Physical Component Summary (PCS)[a]**						*0.943*
Baseline	72	27.45 (9.67)	59	27.12 (8.64)	N/A	N/A
3 Months	51	32.05 (10.81)	40	32.27 (10.24)	0.69 (-3.22, 4.60)	0.728
6 Months	44	34.51 (11.57)	36	33.61 (10.86)	0.72 (-3.77, 5.21)	0.751
12 Months	43	33.05 (12.41)	38	34.10 (11.08)	1.55 (-3.43, 6.52)	0.540
**SF-36 Mental Component Summary (MCS)[a]**						*0.522*
Baseline	72	44.92 (11.72)	59	47.30 (12.11)	N/A	N/A
3 Months	51	51.38 (11.91)	40	56.52 (8.42)	3.12 (-1.36, 7.61)	0.171
6 Months	44	52.57 (13.36)	36	55.94 (10.28)	1.02 (-4.66, 6.71)	0.722
12 Months	43	50.54 (11.12)	38	55.57 (10.32)	2.36 (-3.15, 7.86)	0.398
**Patient Health Questionnaire-9**						*0.851*
Baseline	61	9.37 (5.77)	51	6.66 (5.82)	N/A	N/A
3 Months	50	6.60 (5.94)	40	3.30 (3.15)	-0.29 (-2.74, 2.16)	0.816
6 Months	44	5.84 (6.18)	38	3.26 (4.01)	0.41 (-2.06, 2.88)	0.744
12 Months	43	6.19 (5.69)	38	3.76 (4.97)	0.12 (-2.33, 2.58)	0.921
**Generalized Anxiety Disorder-7**						*0.898*

© 2023 Khan BA et al. *JAMA Network Open*.

Baseline	62	6.36 (6.41)	52	5.33 (4.97)		
3 Months	50	4.78 (5.89)	40	2.70 (2.98)	-0.40 (-2.69, 1.90)	0.733
6 Months	44	3.34 (4.45)	38	2.03 (3.06)	0.15 (-2.45, 2.75)	0.908
12 Months	43	4.40 (5.43)	38	3.00 (4.67)	0.14 (-2.15, 2.43)	0.904
**RBANS Z-Score[b]**						*0.145*
Baseline	46	-0.13 (0.66)	48	-0.25 (0.87)		
3 Months	43	0.06 (0.60)	35	-0.25 (0.75)	-0.09 (-0.31, 0.14)	0.454
6 Months	41	0.23 (0.59)	33	0.22 (0.77)	0.12 (-0.15, 0.39)	0.391
12 Months	40	0.24 (0.69)	31	0.12 (0.74)	-0.02 (-0.31, 0.27)	0.880

[a]The Medical Outcomes Study 36-Item Short-Form Health Survey (SF-36) consists of eight domains. It yields two summary scores (PCS: physical component score, and MCS: mental component score). MCS and PCS range from 0 to 100, and higher scores indicate better health status. A difference of 2 or more points is considered clinically meaningful for both the PCS and MCS subscales of SF-36. [b]The overall RBANS z-score is the mean of the 7-item z-scores.

© 2023 Khan BA et al. *JAMA Network Open*.

eTable 10. Comparison of Study Outcomes Between Study Groups in Participants With Baseline PHQ-9 ≥10

Outcomes	Control		m-CCRP Intervention		Estimated Difference in Change from Baseline (95% CI)	P-value
	N	Mean (SD)	N	Mean (SD)		
**SF-36 PCS[a]**						*0.992*
Baseline	84	25.42 (8.40)	74	26.44 (9.40)	N/A	
3 Months	60	30.31 (10.95)	55	30.18 (8.56)	-0.30 (-3.86, 3.27)	0.870
6 Months	57	31.70 (12.80)	54	31.03 (10.19)	-0.65 (-4.89, 3.60)	0.764
12 Months	53	32.62 (12.45)	49	32.92 (11.32)	-0.34 (-4.55, 3.87)	0.873
**SF-36 MCS[a]**						*0.841*
Baseline	84	39.96 (10.77)	74	39.76 (11.44)	N/A	
3 Months	60	45.67 (13.25)	55	44.58 (13.83)	-0.56 (-5.16, 4.04)	0.812
6 Months	57	48.03 (12.38)	54	45.07 (13.14)	-1.70 (-6.87, 3.47)	0.517
12 Months	53	47.92 (11.09)	49	44.46 (13.55)	-1.98 (-6.79, 2.82)	0.416
**Patient Health Questionnaire-9**						*0.207*
Baseline	85	15.02 (4.26)	76	15.22 (4.03)	N/A	
3 Months	61	9.49 (7.18)	54	10.04 (7.07)	-0.26 (-2.49, 1.97)	0.820
6 Months	56	7.66 (5.84)	53	10.00 (7.02)	1.38 (-0.97, 3.73)	0.247
12 Months	54	7.28 (5.62)	49	10.04 (7.14)	1.54 (-0.69, 3.78)	0.175
**Generalized Anxiety Disorder-7**						*0.465*
Baseline	84	11.85 (5.76)	76	12.26 (5.51)	N/A	

© 2023 Khan BA et al. *JAMA Network Open*.

3 Months	61	7.43 (6.61)	54	8.60 (6.48)	0.63 (-1.57, 2.83)	0.570
6 Months	56	6.20 (5.47)	53	8.79 (6.41)	1.66 (-0.60, 3.91)	0.149
12 Months	54	7.22 (5.60)	49	8.67 (7.23)	0.37 (-1.99, 2.73)	0.756
**RBANS Z-Score[b]**						*0.799*
Baseline	66	-0.06 (0.76)	61	-0.03 (0.66)	N/A	
3 Months	52	0.09 (0.67)	48	-0.10 (0.56)	-0.11 (-0.32, 0.11)	0.326
6 Months	51	0.31 (0.70)	50	0.24 (0.57)	-0.07 (-0.28, 0.15)	0.552
12 Months	46	0.28 (0.73)	42	0.20 (0.64)	-0.07 (-0.30, 0.16)	0.554

[a]The Medical Outcomes Study 36-Item Short-Form Health Survey (SF-36) consists of eight domains. It yields two summary scores (PCS: physical component score, and MCS: mental component score). MCS and PCS range from 0 to 100, and higher scores indicate better health status. A difference of 2 or more points is considered clinically meaningful for both the PCS and MCS subscales of SF-36. [b]The overall RBANS z-score is the mean of the 7-item z-scores.

© 2023 Khan BA et al. *JAMA Network Open*.

eTable 11. Comparison of Study Outcomes by Number of m-CCRP Care Coordinator-Patient Contacts

	Control		m-CCRP						P-value
			0 – 1 Contacts		2 – 12 Contacts		13+ Contacts		
	N	Mean (SD)	N	Mean (SD)	N	Mean (SD)	N	Mean (SD)	
**SF-36 Physical Component Summary[a]**									*0.898*
Baseline	222	28.01 (9.89)	80	28.40 (10.62)	72	26.00 (8.13)	69	28.92 (10.33)	
3 Months	164	32.40 (11.28)	42	34.63 (11.85)	56	29.47 (9.58)	66	35.30 (10.74)	
6 Months	159	34.73 (12.32)	39	35.19 (10.59)	45	31.04 (11.02)	67	37.06 (11.25)	
12 Months	146	35.29 (12.20)	41	36.99 (12.66)	47	32.89 (12.60)	65	38.14 (12.15)	
**SF-36 Mental Component Summary[a]**									*0.502*
Baseline	222	44.85 (11.43)	80	47.75 (10.71)	72	45.05 (13.40)	69	47.34 (11.30)	
3 Months	164	49.90 (12.46)	42	48.08 (13.29)	56	49.19 (12.44)	66	53.13 (9.87)	
6 Months	159	50.50 (12.05)	39	49.07 (13.22)	45	47.81 (12.44)	67	52.02 (11.09)	
12 Months	146	51.12 (10.23)	41	50.25 (11.93)	47	49.00 (11.72)	65	50.12 (11.82)	
**Patient Health Questionnaire - 9**									*0.200*
Baseline	202	9.10 (6.19)	69	7.94 (5.77)	69	10.12 (6.64)	67	6.89 (5.85)	
3 Months	164	7.02 (6.38)	39	8.77 (7.94)	56	6.84 (5.84)	66	4.65 (4.61)	
6 Months	157	6.22 (5.68)	38	7.87 (7.74)	46	8.02 (6.51)	66	4.58 (4.92)	
12 Months	147	5.66 (5.37)	41	6.66 (6.81)	47	6.43 (6.02)	65	5.14 (5.74)	
**Generalized Anxiety Disorder - 7**									*0.888*
Baseline	203	7.47 (6.24)	70	7.57 (6.18)	69	8.42 (6.64)	68	5.29 (5.28)	
3 Months	162	5.50 (5.85)	39	6.91 (7.20)	56	6.14 (5.59)	66	3.61 (4.31)	
6 Months	157	5.15 (5.36)	38	6.37 (6.44)	46	6.67 (6.42)	66	3.48 (4.57)	
12 Months	147	4.82 (5.25)	41	5.71 (6.74)	47	5.77 (6.19)	65	4.09 (5.51)	
**RBANS Z-score[b]**									*0.758*
Baseline	163	-0.01 (0.73)	58	-0.01 (0.84)	58	-0.08 (0.66)	60	0.12 (0.72)	
3 Months	146	0.08 (0.66)	29	-0.07 (0.89)	48	-0.12 (0.63)	64	0.08 (0.66)	
6 Months	142	0.34 (0.68)	33	0.18 (0.80)	41	0.15 (0.69)	61	0.42 (0.66)	

© 2023 Khan BA et al. *JAMA Network Open*.

12 Months	133	0.26 (0.68)	34	0.26 (0.70)	37	0.21 (0.73)	58	0.32 (0.70)	

[a]The Medical Outcomes Study 36-Item Short-Form Health Survey (SF-36) consists of eight domains. It yields two summary scores (PCS: physical component score, and MCS: mental component score). MCS and PCS range from 0 to 100, and higher scores indicate better health status. A difference of 2 or more points is considered clinically meaningful for both the PCS and MCS subscales of SF-36. [b]The overall RBANS z-score is the mean of the 7-item z-scores.

© 2023 Khan BA et al. *JAMA Network Open*.

eTable 12. Comparison of Study Outcomes When the m-CCRP Group is Divided into Those With >25% Reduction in Healthy Aging Brain Care Monitor (HABC-M Self Report) Symptom Scores and Those With No Reduction

Outcomes	Control		m-CCRP no Reduction in HABC-M SR[a]		m-CCRP 25% Reduction in HABC-M SR[a]		P-value
	N	Mean (SD)	N	Mean (SD)	N	Mean (SD)	
**SF-36 Physical Component Summary[b]**							*0.069*
Baseline	222	28.01 (9.89)	146	28.03 (9.80)	75	27.29 (9.90)	
3 Months	164	32.40 (11.28)	102	32.89 (10.95)	62	33.54 (10.97)	
6 Months	159	34.73 (12.32)	95	33.44 (10.92)	56	37.05 (11.49)	
12 Months	146	35.29 (12.20)	94	34.48 (12.46)	59	38.98 (12.29)	B[c]
**SF-36 Mental Component Summary[b]**							*0.016*
Baseline	222	44.85 (11.43)	146	47.07 (11.60)	75	46.11 (12.32)	
3 Months	164	49.90 (12.46)	102	49.13 (12.64)	62	52.73 (10.12)	
6 Months	159	50.50 (12.05)	95	47.81 (12.28)	56	53.73 (11.01)	A[d]
12 Months	146	51.12 (10.23)	94	47.47 (12.07)	59	53.54 (10.27)	A[d]
**Patient Health Questionnaire - 9**							*0.018*
Baseline	202	9.10 (6.19)	132	8.07 (6.12)	73	8.81 (6.41)	
3 Months	164	7.02 (6.38)	99	7.28 (6.69)	62	5.02 (4.97)	
6 Months	157	6.22 (5.68)	92	7.62 (6.83)	58	4.64 (5.23)	
12 Months	147	5.66 (5.37)	94	7.12 (6.39)	59	4.07 (5.22)	A[d]
**Generalized Anxiety Disorder-7**							*0.080*
Baseline	203	7.47 (6.24)	134	7.49 (6.30)	73	6.40 (5.93)	
3 Months	162	5.50 (5.85)	99	5.89 (6.21)	62	4.33 (4.71)	
6 Months	157	5.15 (5.36)	92	6.12 (6.07)	58	3.72 (5.20)	
12 Months	147	4.82 (5.25)	94	6.34 (6.55)	59	2.97 (4.58)	
**RBANS Z-score[e]**							*0.107*
Baseline	163	-0.01 (0.73)	111	0.00 (0.74)	65	0.03 (0.76)	
3 Months	146	0.08 (0.66)	84	-0.12 (0.74)	57	0.12 (0.63)	

© 2023 Khan BA et al. *JAMA Network Open*.

6 Months	142	0.34 (0.68)	81	0.21 (0.71)	54	0.38 (0.71)	
12 Months	133	0.26 (0.68)	75	0.19 (0.67)	54	0.39 (0.73)	

[a]HABC-M SR: Healthy Aging Brain Care Monitor Self-Report
[b]The Medical Outcomes Study 36-Item Short-Form Health Survey (SF-36) consists of eight domains. It yields two summary scores (PCS: physical component score, and MCS: mental component score). MCS and PCS range from 0 to 100, and higher scores indicate better health status. A difference of 2 or more points is considered clinically meaningful for both the PCS and MCS subscales of SF-36.
[c]B-significant difference between group with 25% symptom reduction and attention control
[d]A-significant difference between group with <25% symptom reduction and attention control
[e]The overall RBANS z-score is the mean of the 7-item z-scores.

© 2023 Khan BA et al. *JAMA Network Open*.

eTable 13. **Effect of m-CCRP Intervention on Patient Outcomes Pre and During COVID-19 Pandemic**

Outcomes	Pre-COVID-19						During COVID-19					
	Control		m-CCRP Intervention		Predicted Difference in Differences (95% CI)	P-value	Control		m-CCRP Intervention		Predicted Difference in Differences (95% CI)	P-value
	N	Mean (SD)	N	Mean (SD)			N	Mean (SD)	N	Mean (SD)		
**SF-36 Physical Component Summary[a]**												
Baseline	153	28.07 (9.91)	151	28.10 (9.76)			69	27.88 (9.93)	70	27.08 (9.96)		
3 Months	100	32.94 (11.43)	105	33.51 (11.10)	1.61 (-1.22, 4.45)	0.264	64	31.55 (11.09)	59	32.47 (10.67)	1.02 (-2.77, 4.82)	0.596
6 Months	93	33.60 (12.33)	88	35.54 (11.49)	2.70 (-0.51, 5.90)	0.099	66	36.32 (12.21)	63	33.72 (10.88)	-0.13 (-4.20, 3.93)	0.948
12 Months	75	34.66 (12.13)	80	37.18 (13.63)	2.97 (-0.54, 6.46)	0.097	71	35.95 (12.33)	73	35.16 (11.24)	0.70 (-3.36, 4.77)	0.734
**SF-36 Mental Component Summary[a]**												
Baseline	153	44.82 (11.49)	151	47.97 (11.66)			69	44.93 (11.36)	70	44.10 (11.86)		
3 Months	100	51.53 (12.33)	105	51.39 (11.40)	-2.22 (-5.58, 1.14)	0.195	64	47.35 (12.32)	59	48.90 (12.54)	1.79 (-2.74, 6.33)	0.438
6 Months	93	51.55 (12.96)	88	50.23 (11.70)	-3.48 (-7.25, 0.28)	0.070	66	49.03 (10.54)	63	49.69 (12.81)	2.26 (-2.61, 7.12)	0.363
12 Months	75	52.10 (9.47)	80	49.30 (11.64)	-5.75 (-9.37, -2.12)	0.002	71	50.07 (10.94)	73	50.37 (11.93)	2.19 (-2.27, 6.66)	0.335
**Patient Health Questionnaire-9**												
Baseline	138	9.05 (6.37)	137	8.16 (6.04)			64	9.20 (5.81)	68	8.68 (6.60)		
3 Months	101	6.58 (6.09)	102	6.11 (6.10)	-0.29 (-1.89, 1.32)	0.728	63	7.73 (6.82)	59	6.93 (6.30)	0.29 (-1.88, 2.46)	0.791
6 Months	91	5.69 (5.57)	87	6.25 (6.23)	0.77 (-1.00, 2.54)	0.393	66	6.95 (5.78)	63	6.76 (6.68)	0.001 (-2.30, 2.30)	0.999
12 Months	76	5.41 (5.45)	80	5.55 (5.74)	0.63 (-1.09, 2.35)	0.471	71	5.93 (5.32)	73	6.37 (6.55)	0.43 (-1.73, 2.59)	0.696
**Generalized Anxiety Disorder-7**												
Baseline	139	7.18 (6.17)	138	6.61 (5.98)			64	8.11 (6.39)	69	8.10 (6.48)		

© 2023 Khan BA et al. *JAMA Network Open*.

3 Months	99	5.19 (5.88)	102	4.89 (5.65)	-0.46 (-1.99, 1.06)	0.551	63	5.98 (5.82)	59	5.98 (5.81)	0.98 (-1.10, 3.05)	0.356
6 Months	91	4.70 (5.42)	87	5.39 (6.00)	0.57 (-1.13, 2.27)	0.510	66	5.77 (5.25)	63	4.92 (5.66)	-0.59 (-2.81, 1.64)	0.603
12 Months	76	4.86 (5.39)	80	4.79 (6.00)	-0.09 (-1.79, 1.61)	0.915	71	4.77 (5.13)	73	5.32 (6.20)	0.67 (-1.45, 2.80)	0.535
**RBANS 7-Item Z-score[b]**												
Baseline	111	-0.12 (0.73)	113	-0.07 (0.77)			52	0.22 (0.66)	63	0.16 (0.68)		
3 Months	88	0.06 (0.63)	89	-0.10 (0.76)	-0.06 (-0.22, 0.10)	0.472	58	0.12 (0.71)	52	0.11 (0.59)	0.01 (-0.21, 0.23)	0.927
6 Months	81	0.34 (0.58)	79	0.23 (0.75)	-0.01 (-0.17, 0.16)	0.930	61	0.34 (0.80)	56	0.35 (0.64)	-0.02 (-0.24, 0.19)	0.830
12 Months	70	0.15 (0.66)	69	0.23 (0.70)	0.08 (-0.10, 0.27)	0.371	63	0.38 (0.69)	60	0.32 (0.71)	-0.002 (-0.23, 0.22)	0.986

[a]The Medical Outcomes Study 36-Item Short-Form Health Survey (SF-36) consists of eight domains. It yields two summary scores (PCS: physical component score, and MCS: mental component score). MCS and PCS range from 0 to 100, and higher scores indicate better health status. A difference of 2 or more points is considered clinically meaningful for both the PCS and MCS subscales of SF-36. [b]The RBANS overall z-score is the mean of the 7-item z-scores.

© 2023 Khan BA et al. *JAMA Network Open*.

eTable 14. Effect of m-CCRP Intervention on Patient Outcomes by Discharge Status

Outcomes	Not Discharged Home						Discharged Home					
	Control		m-CCRP Intervention		Predicted Difference in Differences	P-value	Control		m-CCRP Intervention		Predicted Difference in Differences	P-value
	N	Mean (SD)	N	Mean (SD)			N	Mean (SD)	N	Mean (SD)		
**SF-36 Physical Component Summary[a]**												
Baseline	89	25.88 (9.27)	87	26.38 (9.38)			133	29.43 (10.08)	134	28.68 (10.02)		
3 Months	60	29.58 (9.81)	61	29.81 (8.92)	0.68 (-3.12, 4.49)	0.725	104	34.02 (11.79)	103	35.11 (11.55)	1.83 (-1.07, 4.74)	0.215
6 Months	55	32.20 (11.51)	55	31.61 (11.11)	0.57 (-3.71, 4.85)	0.794	104	36.07 (12.57)	96	36.60 (10.96)	1.91 (-1.31, 5.13)	0.244
12 Months	48	33.07 (12.54)	50	34.10 (11.89)	1.10 (-3.46, 5.65)	0.637	98	36.37 (11.95)	103	37.24 (12.79)	1.95 (-1.37, 5.27)	0.249
**SF-36 Mental Component Summary[a]**												
Baseline	89	42.86 (10.45)	87	46.63 (12.28)			133	46.19 (11.89)	134	46.82 (11.58)		
3 Months	60	50.78 (13.07)	61	52.19 (11.36)	-2.26 (-6.70, 2.19)	0.319	104	49.39 (12.12)	103	49.49 (12.07)	-0.04 (-3.45, 3.37)	0.983
6 Months	55	50.97 (12.72)	55	51.45 (11.19)	-2.23 (-7.17, 2.70)	0.375	104	50.25 (11.73)	96	49.17 (12.62)	-0.88 (-4.62, 2.86)	0.644
12 Months	48	52.12 (9.24)	50	50.25 (12.16)	-4.49 (-9.17, 0.20)	0.061	98	50.63 (10.69)	103	49.60 (11.61)	-1.26 (-4.74, 2.21)	0.475
**Patient Health Questionnaire-9**												
Baseline	81	9.98 (6.47)	77	9.19 (6.00)			121	8.51 (5.94)	128	7.81 (6.32)		
3 Months	59	7.66 (6.93)	60	5.88 (5.87)	-0.91 (-3.07, 1.25)	0.410	105	6.67 (6.06)	101	6.72 (6.34)	0.37 (-1.26, 2.01)	0.653
6 Months	55	6.05 (5.77)	54	6.19 (6.16)	0.25 (-2.07, 2.56)	0.835	102	6.31 (5.65)	96	6.63 (6.57)	0.51 (-1.24, 2.26)	0.566
12 Months	48	5.25 (4.86)	50	5.38 (6.11)	0.18 (-2.05, 2.42)	0.873	99	5.86 (5.62)	103	6.21 (6.15)	0.67 (-0.97, 2.31)	0.425
**Generalized Anxiety Disorder-7**												

© 2023 Khan BA et al. *JAMA Network Open*.

Baseline	82	7.78 (6.42)	77	7.40 (6.29)			121	7.26 (6.13)	130	6.93 (6.13)		
3 Months	58	5.43 (6.45)	60	4.46 (5.60)	-0.71 (-2.74, 1.33)	0.494	104	5.54 (5.52)	101	5.78 (5.75)	0.50 (-1.05, 2.04)	0.529
6 Months	55	4.78 (5.52)	54	4.54 (5.72)	-0.28 (-2.51, 1.94)	0.804	102	5.35 (5.29)	96	5.56 (5.91)	0.35 (-1.34, 2.03)	0.687
12 Months	48	3.92 (4.55)	50	4.44 (5.66)	0.39 (-1.82, 2.60)	0.727	99	5.25 (5.53)	103	5.33 (6.28)	0.35 (-1.27, 1.96)	0.672
**RBANS 7-Item Z-score[b]**												
Baseline	58	-0.27 (0.78)	66	-0.19 (0.75)			105	0.13 (0.66)	110	0.13 (0.71)		
3 Months	53	0.00 (0.78)	53	-0.12 (0.70)	-0.05 (-0.27, 0.17)	0.676	93	0.13 (0.58)	88	0.04 (0.70)	-0.05 (-0.21, 0.11)	0.534
6 Months	50	0.34 (0.66)	50	0.21 (0.74)	-0.05 (-0.28, 0.17)	0.627	92	0.34 (0.69)	85	0.32 (0.69)	-0.02 (-0.18, 0.14)	0.835
12 Months	44	0.25 (0.71)	44	0.15 (0.72)	0.06 (-0.18, 0.30)	0.627	89	0.27 (0.67)	85	0.33 (0.69)	0.004 (-0.17, 0.17)	0.963

[a]The Medical Outcomes Study 36-Item Short-Form Health Survey (SF-36) consists of eight domains. It yields two summary scores (PCS: physical component score, and MCS: mental component score). MCS and PCS range from 0 to 100, and higher scores indicate better health status. A difference of 2 or more points is considered clinically meaningful for both the PCS and MCS subscales of SF-36. [b]The RBANS overall z-score is the mean of the 7-item z-scores.

© 2023 Khan BA et al. *JAMA Network Open*.

eTable 15. Comparison of Patient Baseline Characteristics by Completion Status at 12 Months

Characteristics	m-CCRP (n=233)			Control (n=233)			P-value
	Completed (n=153)	Died (n=24)	Other* (n=56)	Completed (n=147)	Died (n=38)	Other* (n=48)	
**Age**	54.4 (13.9)	63.0 (12.3)	54.2 (15.2)	56.7 (13.7)	60.3 (12.5)	55.1 (17.6)	0.464
**Sex**							0.285
Male n (%)	71 (46.4)	12 (50.0)	25 (44.6)	62 (42.2)	18 (47.4)	28 (58.3)	
Female n (%)	82 (53.6)	12 (50.0)	31 (55.4)	85 (57.8)	20 (52.6)	20 (41.7)	
**Race**							0.907
African-American n (%)	68 (44.4)	7 (29.2)	19 (33.9)	52 (35.4)	15 (39.5)	11 (22.9)	
Other n (%)	2 (1.3)	1 (4.2)	0 (0.0)	4 (2.7)	0 (0.0)	0 (0.0)	
White n (%)	82 (53.6)	16 (66.7)	36 (64.3)	87 (59.2)	22 (57.9)	37 (77.1)	
Unknown n (%)	1 (0.7)	0 (0.0)	1 (1.8)	4 (2.7)	1 (2.6)	0 (0.0)	
Hispanic n (%)	0 (0.0)	0 (0.0)	0 (0.0)	4 (2.7)	0 (0.0)	0 (0.0)	0.999
**Education**							0.545
0-11 years n (%)	23 (15.0)	5 (23.8)	5 (10.0)	16 (11.0)	2 (5.9)	8 (17.0)	
12 years n (%)	55 (40.0)	6 (28.6)	26 (52.0)	52 (35.9)	16 (47.1)	19 (40.4)	
Vocational School n (%)	3 (2.0)	1 (4.8)	0 (0.0)	6 (4.1)	0 (0.0)	2 (4.3)	
Some College n (%)	22 (14.4)	3 (14.3)	10 (20.0)	30 (20.7)	7 (20.6)	8 (17.0)	
Associate's Degree n (%)	21 (13.7)	2 (9.5)	3 (6.0)	10 (6.9)	3 (8.8)	4 (8.5)	
Bachelor's Degree n (%)	17 (11.1)	3 (14.3)	4 (8.0)	18 (12.4)	1 (2.9)	4 (8.5)	
Master's Degree/Doctorate n (%)	12 (7.8)	1 (4.8)	2 (4.0)	13 (9.0)	5 (14.7)	2 (4.3)	
**APACHE II**[a]	24.0 (8.8)	23.6 (10.2)	24.3 (8.9)	25.7 (8.1)	26.9 (9.1)	25.6 (8.5)	0.756
**Charlson Comorbidity Index**	2.1 (1.9)	2.6 (2.0)	1.9 (1.9)	2.0 (2.1)	3.3 (2.6)	2.3 (2.9)	0.410
**Activities of Daily Living (ADL)**[b]	5.4 (1.3)	5.4 (1.1)	5.7 (0.7)	5.5 (1.3)	5.3 (1.4)	5.1 (1.7)	0.139
**Instrumental Activities of Daily Living (IADL)**[c]	6.9 (1.8)	5.9 (1.8)	7.2 (1.9)	7.0 (1.6)	6.5 (2.1)	7.1 (2.0)	0.665
**IQCODE**[d]	24.0 (8.8)	23.6 (10.2)	24.3 (8.9)	25.7 (8.1)	26.9 (9.1)	25.6 (8.5)	0.666
**Site, n (%)**							0.490
Methodist	98 (64.0)	14 (58.3)	47 (83.9)	103 (70.1)	23 (60.5)	33 (68.8)	
University	9 (5.9)	1 (4.2)	2 (3.6)	5 (3.4)	3 (7.9)	4 (8.3)	
Eskenazi	45 (29.4)	8 (33.3)	7 (12.5)	37 (25.2)	12 (31.6)	11 (22.9)	
West	1 (0.7)	1 (4.2)	0 (0.0)	2 (1.4)	0 (0.0)	0 (0.0)	
**Insurance, n (%)**							0.968
Medicaid/Medicare	28 (18.7)	6 (26.1)	9 (16.4)	25 (17.1)	11 (30.6)	8 (17.0)	
Medicaid	18 (12.0)	5 (21.7)	11 (20.0)	20 (13.7)	6 (16.7)	8 (17.0)	

© 2023 Khan BA et al. *JAMA Network Open*.

Medicare	17 (11.3)	3 (13.0)	4 (7.3)	20 (13.7)	10 (27.9)	8 (17.0)	
Medicare/Private	19 (12.7)	4 (17.4)	7 (12.7)	17 (11.6)	4 (11.1)	8 (17.0)	
Private	41 (27.3)	4 (!7.4)	16 (29.1)	39 (26.7)	5 (13.9)	9 (19.2)	
Other	20 (13.3)	1 (4.4)	6 (10.9)	17 (11.6)	0 (0.0)	2 (4.3)	
None	7 (4.7)	0 (0.0)	2 (3.6)	8 (5.5)	0 (0.0)	4 (8.5)	
**Route of Ventilation, n (%)**							0.996
Invasive Mechanical ventilation	150 (98.0)	22 (91.7)	52 (92.9)	139 (94.6)	38 (100.0)	48 (100.0)	
Non-invasive Positive Pressure Ventilation or Heated High Flow Nasal Cannula	3 (2.0)	2 (8.3)	4 (7.1)	8 (5.4)	0 (0.0)	0 (0.0)	
**ICU characteristics**							
Duration of mechanical ventilation (hours)	115.3 (132.6)	151.4 (230.2)	81.6 (80.0)	99.6 (116.8)	163.7 (194.3)	146.8 (190.7)	0.045 (0.006)
Length of Hospital stay (days)	19.1 (14.9)	19.1 (12.3)	17.1 (10.5)	16.2 (10.2)	23.5 (22.7)	19.9 (12.2)	0.054 (0.145)
Length of ICU stay (days)	11.5 (8.7)	12.2 (10.8)	8.7 (5.9)	9.4 (6.1)	16.0 (19.7)	10.9 (7.8)	0.022 (0.015)
**Service**							0.636
Medical ICU[e] n (%)	88 (57.5)	16 (66.7)	32 (57.1)	93 (63.3)	29 (76.3)	26 (54.2)	
Surgical ICU n (%)	65 (42.5)	8 (33.3)	24 (42.9)	54 (36.7)	9 (23.7)	22 (45.8)	
**Discharge Location n(%)**							0.988
Home	103 (67.3)	6 (25.0)	32 (57.1)	99 (67.4)	13 (34.2)	23 (47.9)	
Inpatient Rehab	24 (15.7)	11 (45.9)	17 (30.4)	24 (16.3)	14 (36.8)	12 (25.0)	
Acute Rehab	15 (9.8)	3 (12.5)	4 (7.1)	15 (10.2)	3 (7.9)	5 (10.4)	
LTAC	8 (5.2)	2 (8.3)	1 (1.8)	6 (4.1)	4 (10.5)	2 (4.2)	
Skilled Nursing Facility	3 (2.0)	1 (4.2)	2 (3.6)	3 (2.0)	2 (5.3)	3 (6.2)	
Other	0 (0.0)	1 (4.2)	0 (0.0)	0 (0.0)	2 (5.3)	3 (6.2)	

Data presented as mean (SD) and median (IQR) unless otherwise stated
[a] APACHE: Acute Physiology and Chronic Health evaluation
[b] ADLs assessed by Katz Scale
[c] IADLs assessed by Lawton Scale
[d] IQCODE: Informant Questionnaire on Cognitive Decline in the Elderly
[e] ICU: Intensive Care Unit
*Other includes patients lost to follow up or patients who withdrew.

© 2023 Khan BA et al. *JAMA Network Open*.

eTable 16. Comparison of Patient Outcomes by Study Groups Over Time Including Adjustment for ICU Length of Stay and Mechanical Ventilation Duration

Outcomes	Control		m-CCRP Intervention		Estimated Difference in Change from Baseline (95% CI)	P-value
	N	Mean (SD)	N	Mean (SD)		
**SF-36 Physical Component Summary[a]**						*0.621*
Baseline	222	28.01 (9.89)	221	27.78 (9.82)	N/A	
3 Months	164	32.40 (11.28)	164	33.14 (10.93)	1.41 (-0.90, 3.71)	0.232
6 Months	159	34.73 (12.32)	151	34.78 (11.24)	1.42 (-1.15, 3.99)	0.279
12 Months	146	35.29 (12.20)	153	36.22 (12.55)	1.64 (-1.04, 4.31)	0.231
**SF-36 Mental Component Summary[a]**						*0.348*
Baseline	222	44.85 (11.43)	221	46.74 (11.83)	N/A	
3 Months	164	49.90 (12.46)	164	50.49 (11.85)	-0.86 (-3.59, 1.87)	0.536
6 Months	159	50.50 (12.05)	151	50.00 (12.13)	-1.39 (-4.39, 1.60)	0.361
12 Months	146	51.12 (10.23)	153	49.81 (11.75)	-2.43 (-5.22, 0.37)	0.089
**Patient Health Questionnaire-9**						*0.619*
Baseline	202	9.10 (6.19)	205	8.33 (6.22)	N/A	
3 Months	164	7.02 (6.38)	161	6.41 (6.17)	-0.06 (-1.37, 1.25)	0.923
6 Months	157	6.22 (5.68)	150	6.47 (6.41)	0.44 (-0.97, 1.85)	0.540
12 Months	147	5.66 (5.37)	153	5.94 (6.13)	0.52 (-0.81, 1.85)	0.442
**Generalized Anxiety Disorder-7**						*0.935*
Baseline	203	7.47 (6.24)	207	7.11 (6.18)	N/A	N/A
3 Months	162	5.50 (5.85)	161	5.29 (5.71)	0.07 (-1.17, 1.30)	0.915
6 Months	157	5.15 (5.36)	150	5.19 (5.85)	0.12 (-1.22, 1.47)	0.856
12 Months	147	4.82 (5.25)	153	5.04 (6.08)	0.36 (-0.95, 1.66)	0.588
**RBANS 7-Item Z-score[b]**						*0.642*
Baseline	163	-0.01 (0.73)	176	0.01 (0.74)	N/A	N/A
3 Months	146	0.08 (0.66)	141	-0.02 (0.71)	-0.05 (-0.18, 0.08)	0.485
6 Months	142	0.34 (0.68)	135	0.28 (0.71)	-0.03 (-0.16, 0.11)	0.688

12 Months	133	0.26 (0.68)	129	0.27 (0.70)	0.02 (-0.12, 0.16)	0.728

Results presented as mean (SD) unless specified otherwise.
[a]The Medical Outcomes Study 36-Item Short-Form Health Survey (SF-36) consists of eight domains. It yields two summary scores (PCS: physical component score, and MCS: mental component score). MCS and PCS range from 0 to 100, and higher scores indicate better health status. A difference of 2 or more points is considered clinically meaningful for both the PCS and MCS subscales of SF-36. *The overall z-score is the mean of the 7-item z-scores.
[b]RBANS: Repeatable Battery for the Assessment of Neuropsychological Status

© 2023 Khan BA et al. *JAMA Network Open*.

eTable 17. Comparison of Patient Outcomes by Study Groups Over Time for m-CCRP patients With at Least 9 Contacts (Per Protocol Analysis)

Outcomes	Control		m-CCRP Intervention		Estimated Difference in Change from Baseline (95% CI)	P-value
	N	Mean (SD)	N	Mean (SD)		
**SF-36 Physical Component Summary[a]**						*0.812*
Baseline	222	28.01 (9.89)	94	27.81 (9.94)	N/A	
3 Months	164	32.40 (11.28)	92	33.04 (10.70)	0.93 (-1.84, 3.70)	0.510
6 Months	159	34.73 (12.32)	91	34.56 (11.62)	1.04 (-2.08, 4.15)	0.513
12 Months	146	35.29 (12.20)	87	36.23 (12.70)	1.56 (-1.61, 4.73)	0.333
**SF-36 Mental Component Summary[a]**						*0.225*
Baseline	222	44.85 (11.43)	94	46.84 (12.18)	N/A	
3 Months	164	49.90 (12.46)	92	52.57 (11.00)	0.40 (-3.02, 3.83)	0.817
6 Months	159	50.50 (12.05)	91	50.82 (11.79)	-0.88 (-4.49, 2.74)	0.635
12 Months	146	51.12 (10.23)	87	50.26 (11.94)	-2.35 (-5.79, 1.08)	0.178
**Patient Health Questionnaire-9**						*0.374*
Baseline	202	9.10 (6.19)	93	7.68 (6.16)	N/A	
3 Months	164	7.02 (6.38)	92	5.17 (5.07)	-0.19 (-1.80, 1.41)	0.813
6 Months	157	6.22 (5.68)	91	5.57 (5.66)	0.48 (-1.23, 2.19)	0.582
12 Months	147	5.66 (5.37)	87	5.41 (5.90)	0.74 (-0.82, 2.31)	0.352
**Generalized Anxiety Disorder-7**						*0.876*
Baseline	203	7.47 (6.24)	94	6.09 (5.98)	N/A	
3 Months	162	5.50 (5.85)	92	4.04 (4.65)	0.20 (-1.32, 1.72)	0.795
6 Months	157	5.15 (5.36)	91	4.25 (5.29)	0.34 (-1.35, 2.03)	0.690
12 Months	147	4.82 (5.25)	87	4.21 (5.64)	0.58 (-0.96, 2.12)	0.462
**RBANS 7-Item Z-score[b]**						*0.910*
Baseline	163	-0.01 (0.73)	81	0.05 (0.70)	N/A	
3 Months	146	0.08 (0.66)	85	0.05 (0.64)	0.00 (-0.16, 0.15)	0.957
6 Months	142	0.34 (0.68)	81	0.32 (0.70)	0.00 (-0.16, 0.16)	0.964

© 2023 Khan BA et al. *JAMA Network Open*.

12 Months	133	0.26 (0.68)	76	0.28 (0.71)	0.04 (-0.13, 0.21)	0.646

Results presented as mean (SD) unless specified otherwise.
[a]The Medical Outcomes Study 36-Item Short-Form Health Survey (SF-36) consists of eight domains. It yields two summary scores (PCS: physical component score, and MCS: mental component score). MCS and PCS range from 0 to 100, and higher scores indicate better health status. A difference of 2 or more points is considered clinically meaningful for both the PCS and MCS subscales of SF-36. *The RBANS overall z-score is the mean of the 7-item z-scores.
[b]RBANS: Repeatable Battery for the Assessment of Neuropsychological Status

© 2023 Khan BA et al. *JAMA Network Open*.

eFigure 1. m-CCRP Intervention Results Based on Selected Sub-Group Analyses

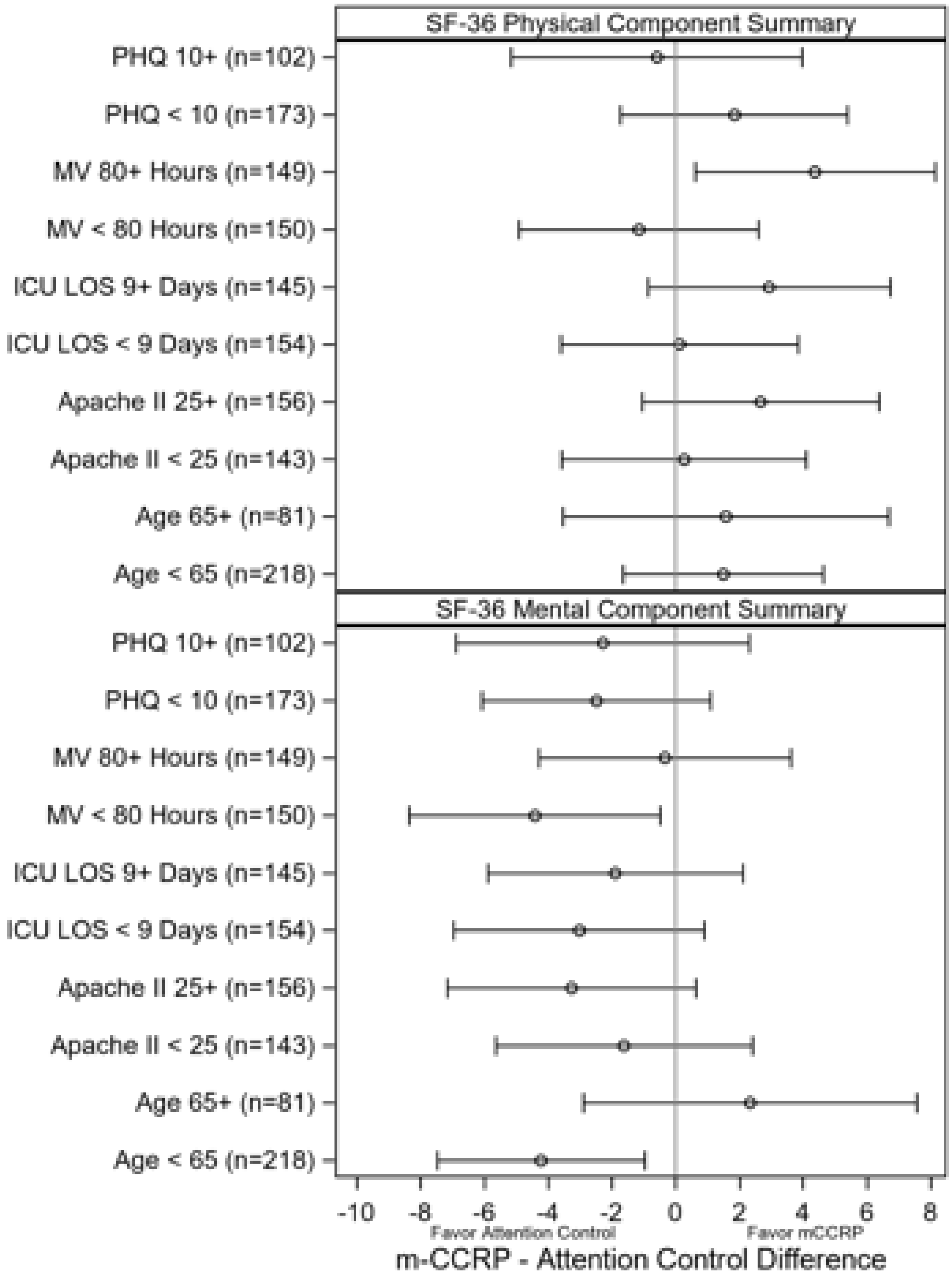

Circles represent estimated difference in change from baseline (mCCRP minus Attention Control), horizontal bars represent 95% CI.

© 2023 Khan BA et al. *JAMA Network Open*.

eFigure 2. Changes in Intervention Measures in the m-CCRP Group

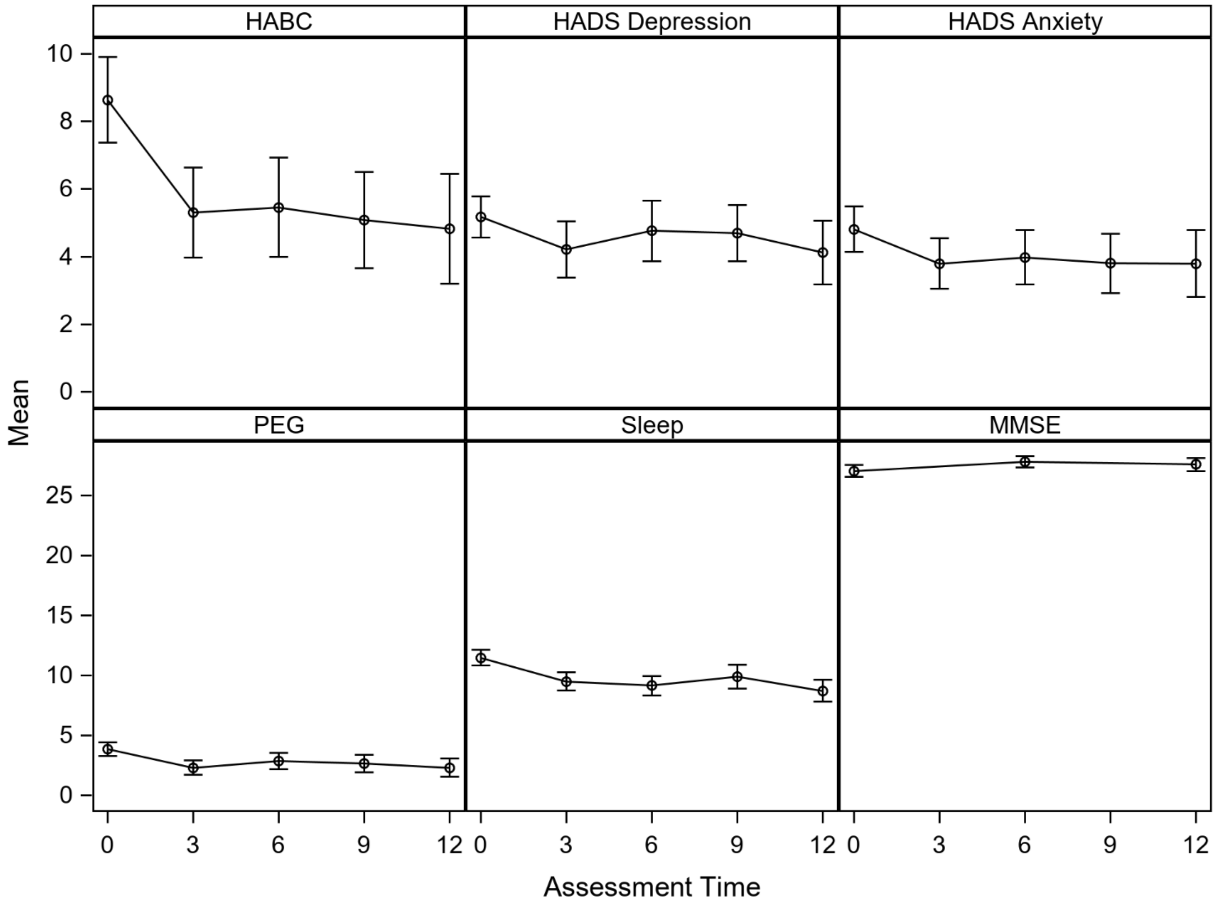

Assessment Time in Months shown. Circles represent mean scores, vertical bars represent 95% CI.

© 2023 Khan BA et al. *JAMA Network Open*.
