# Supplementary material for: Mobile Critical Care Recovery Program for Survivors of Acute Respiratory Failure: A Randomized Clinical Trial
Source: JAMA Netw Open. 2024 Jan 30;7(1):e2353158. doi: 10.1001/jamanetworkopen.2023.53158 (PMC10828910; doi:10.1001/jamanetworkopen.2023.53158)
Supplement: Supplement 3. — Data Sharing Statement [file jamanetwopen-e2353158-s003.pdf]

Data Sharing Statement

Khan. Mobile Critical Care Recovery Program for Survivors of Acute Respiratory Failure. *JAMA Netw Open*. Published January 30, 2024. doi:10.1001/jamanetworkopen.2023.53158

Data
Data available: Yes
Data types: Deidentified participant data
How to access data: Please send requests to bakhan@iu.edu
When available: beginning date: 01-01-2025, end date: 01-01-2026

Supporting Documents
Document types: None

Additional Information
Who can access the data: N/A
Types of analyses: N/A
Mechanisms of data availability: N/A
